# Hepatoma-Derived Growth Factor Secreted from Mesenchymal Stem Cells Reduces Myocardial Ischemia-Reperfusion Injury

**DOI:** 10.1155/2017/1096980

**Published:** 2017-11-14

**Authors:** Yu Zhou, Panpan Chen, Qingnian Liu, Yingchao Wang, Ling Zhang, Rongrong Wu, Jinghai Chen, Hong Yu, Wei Zhu, Xinyang Hu, Jian-An Wang

**Affiliations:** ^1^Department of Cardiology, Second Affiliated Hospital of Zhejiang University School of Medicine, 88 Jiefang Rd, Hangzhou 310009, China; ^2^Cardiovascular Key Laboratory of Zhejiang Province, 88 Jiefang Rd, Hangzhou 310009, China

## Abstract

**Objectives:**

The present study aimed to explore the major factors that account for the beneficial effects of mesenchymal stem cells (MSCs).

**Methods:**

Using isobaric tags for relative and absolute quantitation method, hepatoma-derived growth factor (HDGF) was identified as an important factor secreted by MSCs, but not by cardiac fibroblasts (CFs). The protective effects of conditioned medium (CdM) from MSCs or CFs were tested by using either H9C2 cells that were exposed by hypoxia-reoxygenation (H/R) insult or an *in vivo* mouse model of myocardial ischemia-reperfusion.

**Results:**

Compared to CF-CdM, MSC-CdM conferred protection against reperfusion injury. CdM obtained from MSCs that were treated with HDGF-targeted shRNA failed to offer any protection *in vitro*. In addition, administration of recombinant HDGF alone recapitulated the beneficial effects of MSC-CdM, which was associated with increased protein kinase C epsilon (PKC*ε*) phosphorylation, enhanced mitochondria aldehyde dehydrogenase family 2 activity, and decreased 4-hydroxy-2-nonenal accumulation. A significant decrease in infarct size and ameliorated cardiac dysfunction was achieved by administration of HDGF in wild-type mice, which was absent in PKC*ε* dominant negative mice, indicating the essential roles of PKC*ε* in HDGF-mediated protection.

**Conclusions:**

HDGF secreted from MSCs plays a key role in the protection against reperfusion injury through PKC*ε* activation.

## 1. Introduction

Ischemic heart diseases, such as myocardial infarction, continue to be one of the leading causes of mortality and morbidity worldwide [1]. Although the application of thrombolysis and vascular intervention salvages myocardium and significantly improves clinical outcomes, reperfusion results in myocardial injury. On reperfusion, the generation of reactive oxygen species (ROS), rapid reintroduction of adenosine triphosphate in the presence of elevated [Ca^2+^]_i_, and induction of the mitochondrial permeability transition lead to hypercontracture as well as apoptotic and oncotic cell death [2]. In addition, reperfusion induces accumulation of 4-hydroxy-2-nonenal (4-HNE) [3], a production of lipid peroxidation [4] that contributes to myocyte injury [5]. However, therapeutic agents to prevent these injuries remain unavailable so far. Therefore, effective cell protection after reperfusion is still an unmet clinical need.

Bone marrow-derived mesenchymal stem cells (MSCs) are one of the most rigorously studied stem cell populations [6], which are now undergoing phase II clinical trials for treating ischemic heart diseases. The low cardiac differential and retention rate of MSCs suggests that the secretion of paracrine factors [7], rather than regenerating the functional myocytes, confers cardioprotection by MSCs. Our previous work [8–11] on rodent and primate models demonstrated that MSC therapy enhanced the survival of cardiomyocytes, reduced myocardial fibrosis, and improved angiogenesis through paracrine effects, in which the factors secreted from MSCs, including leptin [12], miR-211 [8], and heparinase [9], played an important role in cardiac protection. Of note, evidence has been put forward showing that treatment using MSC secretions is sufficient to reduce reperfusion-induced myocardial apoptosis and oxidative stress in both rodent and large animal models [13, 14]. However, the factors that contribute to the beneficial effects of MSCs have not been defined.

In the present study, by isobaric tags for relative and absolute quantitation (iTRAQ) secretomic analysis of either MSCs or cardiac fibroblasts (CFs), we have identified that hepatoma-derived growth factor (HDGF) was one of those factors secreted by MSCs and can confer protection against reperfusion-induced cardiomyocyte death. Treatment of HDGF recombinant protein reduces apoptosis and oxidative stress *in vitro* which in turn can decrease myocardial infarct size in an *in vivo* mouse model in a protein kinase C epsilon- (PKC*ε*-) dependent fashion.

## 2. Materials and Methods

### 2.1. Animals

For detailed methods, please refer to the Supplementary Material available online at https://doi.org/10.1155/2017/1096980. Wild-type (WT) littermates and PKC*ε*-dominant negative (PKC*ε*-DN) mice were kindly provided from Professor Peipei Ping [15]. All animal studies were performed with the approval of the Institutional Animal Care and Use Committee, Zhejiang University and according to the Chinese Guideline for Laboratory Animal Care and Use.

### 2.2. Conditioned Medium Preparation

Mice MSCs isolated from 4- to 5-week-old wild-type (WT) mouse bone marrow were examined for the characteristic surface antigen profile by flow cytometry and cultured as described previously [12]. Cardiac fibroblasts were isolated from WT mice as described previously [11]. MSCs or fibroblasts of 80% confluence were washed with PBS and cultured in serum-free medium for 24 h. The conditioned medium was then centrifugated and concentrated 25-fold less of the original volume using 3 kDa molecular weight cutoff ultrafiltration membranes (Millipore, MA, USA). The concentrated medium was desalted and diluted to 0.5 mg/mL for tail vein injection.

### 2.3. Ischemia-Reperfusion Models and Hemodynamic Measurement

Mice were anesthetized with intraperitoneal injection of pentobarbital sodium (60 mg/kg) and then subjected to the left anterior descending (LAD) coronary artery ligation including silicon tubing on top of the coronary artery with an 8-0 Prolene suture. Fifteen minutes before occlusion, 0.2 mL of vehicle or conditioned medium derived from MSCs (MSC-CdM) or CFs (CF-CdM) was injected via tail vein. After 45 min of ischemia, the silicon tubing was removed to achieve reperfusion. The Evan's blue and 2,3,5-triphenyltetrazolium chloride staining were performed on cardiac tissue sections to identify the area at risk and the infarct area. Hemodynamic assessment was taken at 24 h reperfusion by a 1.4 F pressure catheter inserted through the right carotid artery into the left ventricle (LV). The transducer was connected to the PowerLab system (AD Instruments, Castle Hill, Australia). LV pressure and LV maximum ± dp/dt were recorded and averaged from 15 beats.

### 2.4. Western Blot Analysis

Culture media were precipitated with trichloroacetic acid (1 : 100, vol/vol, overnight incubation at −20°C). The precipitates were rinsed with acetone, prior to be resuspended into lysis buffer. Proteins extracted from cells or heart tissues (40 *μ*g protein for each sample) were electrophoresed on a SDS-PAGE and transferred onto a PVDF membranes (Bio-Rad) and incubated with primary antibodies against phosphorylated PKC*ε* (1 : 500, Santa Cruz, CA, USA), PKC*ε*, cleaved caspase-3, *β*-actin (1 : 1000, all from Cell Signaling Technology, Danvers, MA, USA), or 4-HNE (1 : 500) (both from Abcam, Cambridge, MA, USA). Then, membranes were incubated with HRP-conjugated secondary antibodies and exposed with the Chemiluminescence Detection Kit (Millipore).

### 2.5. Mitochondrial Aldehyde Dehydrogenase Family 2 (ALDH2) Activity Assay

Mitochondria were isolated using a mitochondrial protein extraction kit (Keygentec, Nanjing, China) according to the instruction supplied by the manufacturer. The ALDH2 activity of mitochondria from cardiomyocytes was measured using a mito-ALDH2 activity kit (Abcam) by a SpectraMax 340PC384 Microplate Reader (Molecular Devices, LLC., CA, USA).

### 2.6. Transferase dUTP Nick End Labeling (TUNEL) Assay and Immunofluorescence Staining

Frozen heart tissue sections were fixed and permeabilized before incubated with primary antibodies and respective secondary antibodies. The apoptosis of cells was detected in situ with TUNEL (Roche Applied Science, IN, USA). cTnI antibody (1 : 200 Abcam) was applied as a cardiomyocyte marker with DAPI counterstaining.

### 2.7. Flow Cytometry Analysis of Cell Apoptosis

The Annexin V-FITC/PI Apoptosis Detection Kit was used to evaluate apoptosis of cells. After rinsed with cold PBS, cells were resuspended in 200 *μ*l of binding buffer. Annexin V solution was added to the cells and incubated for 30 min at 4°C. The cells were then incubated with 5 ml propidium iodide (PI) and immediately analyzed with a FACScan. Ten thousand events were acquired on a FACSC-LSR (Becton-Dickinson, San Jose, CA) and analyzed with CellQuest (Becton-Dickinson) software.

### 2.8. Lentivirus Construction and Infection

Construction of the recombinant lentivirus with HDGF was performed by the Genechem Company. For MSC infection, cells were seeded at a density of 1 × 10^5^ cells in a six-well plate and infected with lentiviral vectors with 10 mg/ml polybrene (Millipore, Boston, MA, USA). At 12 hour postinfection, the medium was replaced. Forty-eight hours later, the transfected cells were cultured in a 5% CO_2_-humidified incubator at 37°C.

### 2.9. Quantitative Polymerase Chain Reaction (qPCR) Analysis

Primers for amplification of mouse HDGF genes were used to determine the expression of HDGF in fibroblasts and MSCs. The amplification program consisted of initial denaturation at 95°C for 10 min followed by 40 cycles from 92°C for 15 s, annealing at 60°C for 30 s and extension at 72°C for 15 s. The relative expression levels of each gene were normalized to *β*-actin using the 2-ΔΔCt method.

### 2.10. Protein Digestion and iTRAQ Labeling

For each sample, protein was digested and the peptide mixture was labeled using chemicals from the iTRAQ reagent kit (Applied Biosystems, California, USA). Disulfide bonds were reduced in 5 mM Tris-(2-carboxyethy) phosphine (TCEP) followed by blocking cysteine residues in 10 mM methyl methanethiosulfonate (MMTS), before digestion with sequence-grade modified trypsin (Promega, Madison, WI, USA). For labeling, each iTRAQ reagent was dissolved in isopropanol and added to the respective peptide mixture. The labeled samples were combined and dried.

### 2.11. High pH Reverse Phase Separation

The peptide mixture was fractionated by high pH separation using the AQUITY UPLC system (Waters Corporation, Milford, MA, USA) connected to a reverse phase column(ACQUITY UPLC Peptide C18 column, 2.1 mm × 150 mm, 1.7 *μ*m, 130 Å, Waters Corporation, Milford, MA, USA). High pH separation was performed using a linear gradient. The column was reequilibrated at initial conditions and the column flow rate was maintained at 600 *μ*L/min. Collected fractions were dried in a vacuum concentrator for the next step.

### 2.12. Low pH Nano-HPLC-MS/MS Analysis

The mixed peptides were separated by nano-HPLC (Eksigent Technologies, Dublin, CA, USA) on the secondary RP analytical column (Eksigent, C18, 3 *μ*m, 150 mm **×** 75 *μ*m). Peptides were subsequently eluted using the following gradient conditions with phase B (98% ACN with 0.1% formic acid) from 5 to 45% B (5–70 min), and total flow rate was maintained at 300 nL/min. Electrospray voltage of 2.3 kV versus the inlet of the mass spectrometer was used.

Triple TOF 4600 mass spectrometer was operated in the data-dependent mode to switch automatically between MS and MS/MS acquisition. MS spectra were acquired across the mass range of 350–1250 m/z in high resolution mode using 250 ms accumulation time per spectrum. Tandem mass spectral scanned from 100–1250 m/z in high sensitivity mode with rolling collision energy. The 20 most intense precursors were selected for fragmentation per cycle with dynamic exclusion time of 9 s.

### 2.13. Statistical Analysis

All data are expressed as mean ± SEM and analyzed by SPSS 17 using two-tailed Student's *t*-test between two groups or one-way analysis of variance (ANOVA) when three or more groups were compared. *P* value less than 0.05 was considered as statistical significance.

## 3. Results

### 3.1. MSC-CdM but Not CF-CdM Induced Myocardial Protection against Reperfusion Injury

**Figure 1:**
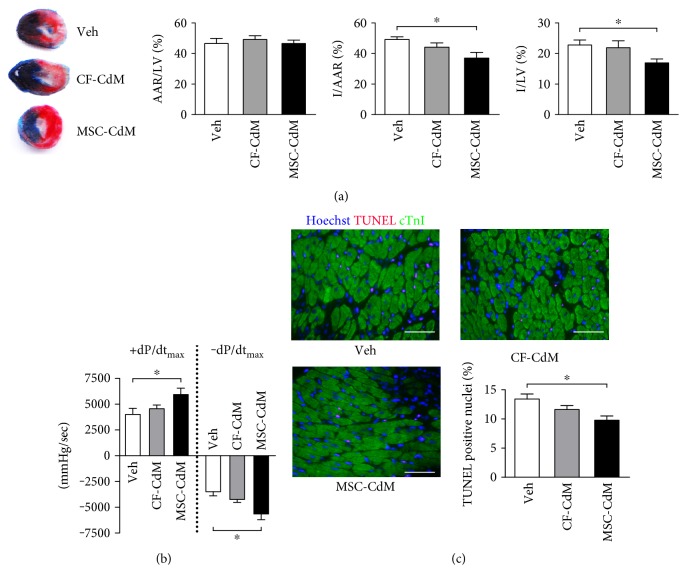
MSC-CdM reduce cardiac reperfusion injury. Wild-type mice were given 5 mg/kg CF-CdM, 5 mg/kg MSC-CdM, or vehicle i.v. 15 min before 45 min of ischemia. MSC-CdM: conditioned medium derived from MSC; CF-CdM: conditioned medium derived from cardiac fibroblasts. (a) Ratio of area at risk (AAR) to left ventricular (LV) area, ratio of infarct size (I) to AAR, and ratio of infarct size to LV after 24 h of reperfusion. Data represent mean ± standard error of mean (SEM) of values from five mice. (b) The maximum rates of rise and decline of left-ventricular pressure (+dp/dt_max_ and −dp/dt_max_) assessed at 24 h reperfusion. Data are mean ± SEM of values from six mice. (c) TUNEL staining at 24 h reperfusion; apoptotic nuclei were stained (red), and cardiomyocytes were detected by cardiac troponin I staining (green). Bar = 50 *μ*M. Data are mean ± SEM of values from three hearts per group, with at least 3000 nuclei examined per heart. ^∗^*P* < 0.05.

To compare the effects of MSC-CdM and CF-CdM on cardiac reperfusion injury, mice were intravenously treated with vehicle, MSC-CdM, or CF-CdM 15 min before occlusion of the LAD coronary artery. These mice were then subjected to 45 min of myocardial ischemia followed by 24 h of reperfusion. The area at risk was not different among the 3 experimental groups, but systemic delivery of MSC-CdM significantly reduced the infarct area/area at risk (I/AAR) and infarct area/left ventricular (I/LV) ratio by 24.6% and 25.6% (*P* < 0.05), respectively, compared with infarcted mice injected with vehicle (Figure 1(a)). In contrast, CF-CdM did not significantly affect the infarct size, showing a similar I/AAR to that in the control mice.

The decreased infarct size in the MSC-CdM group was accompanied by improved functional recovery. Compared with the vehicle group, values for maximal left-ventricular pressure (+dp/dt_max_) and minimal left-ventricular pressure (−dp/dt_max_) measured at 24 h reperfusion were both significantly improved (*P* < 0.05) in MSC-CdM treated mice, which were, however, not observed (*P* > 0.05) in CF-CdM-treated mice (Figure 1(b)). In parallel, LV end-diastolic pressure (LVEDP) was not affected by MSC-CdM, indicating that the increase of +dp/dt_max_ is not due to altered preload (Supplementary Table 1). As Tau, an index of globe left-ventricular relaxation, was not changed by MSC-CdM, the improvement of −dp/dt_max_ may have reflected the higher +dp/dt_max_ in this group.

The extent of apoptosis at 24 h reperfusion was assessed by TUNEL staining (Figure 1(c)). MSC-CdM-treated mice had significantly decreased TUNEL positive myocytes (*P* < 0.05) in the peri-infarct area of the heart compared with controls. However, CF-CdM did not decrease the percentage of apoptotic cells after reperfusion. Similarly, isolated myocytes treated with MSC-CdM exhibited enhanced contractile performance (Supplementary Fig. 1A) and upregulated calcium transient amplitude (Supplementary Fig. 1B) at 2 h reoxygenation following 4 h of hypoxia, all of which were absent in CF-CdM treated cardiomyocytes.

### 3.2. Proteomic Profiles of Secreted Protein in MSC-CdM and CF-CdM and Highly Secreted HDGF from MSCs

**Figure 2:**
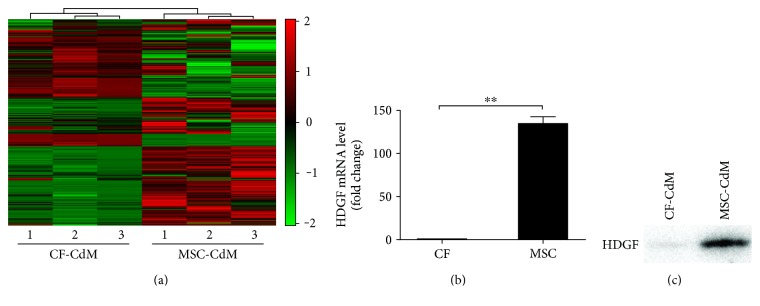
Secretome patterns and different HDGF expression between MSCs and fibroblasts. (a) iTRAQ analysis was applied and 1596 proteins were identified. Hierarchical clustering displayed as a heatmap. Red indicates an increase in expression, and green indicates decreases in expression compared with fibroblasts. (b) HDGF mRNA levels of MSCs and fibroblasts were assessed by RT-qPCR. Data represent mean ± SEM of values from three determinations. (c) Western blot assays on proteins precipitated from the MSC-CdM and CF-CdM. Representative of three independent experiments. ^∗∗^*P* < 0.01.

To identify the specific paracrine factors that were responsible for the beneficial effects of MSC-CdM, the iTRAQ-labeled conditioned media protein samples of MSCs and CFs were analyzed. A total of 1787 proteins with at least 95% confidence were identified in the conditioned medium, among which 1595 proteins had quantification information (Supplementary Table S3). The subcellular localization information of all the identified proteins was annotated by Gene Ontology. As a result, a total of 861 proteins were assigned as extracellular (Figure 2(a)), of which 55 proteins were secreted selectively at higher level in MSC-CdM (>2-fold), while other 53 proteins were found highly secreted in CF-CdM (>2-fold). Functional classification and an enrichment analysis based on molecular functions and biological processes revealed that these 108 differentially secreted proteins fell into many functional categories. We found that the number of proteins varied greatly for the different categories (Supplementary Fig. S2). Compared to CFs, MSCs secreted more proteins that were involved in the categories of “cellular process,” “metabolic process,” and “developmental process.” In addition, MSC-CdM contained more secreted proteins that were related to catalytic activity.

Among the list of 55 proteins specifically highly secreted by MSCs (Table 1), HDGF was selected for further study because this candidate could relay paracrine communication between MSCs and myocytes, as well as exhibit antiapoptosis and proliferation effects [16–19]. To validate that HDGF was secreted by MSCs, RT-qPCR was performed to detect the expression level of HDGF in MSCs and fibroblasts. The mRNA abundance of HDGF in MSCs was significantly higher compared with that in fibroblasts (*P* < 0.05) (Figure 2(b)). These data were consistent with immunoblotting results which showed that HDGF was highly selectively expressed in MSC-CdM with high density (Figure 2(c)). Thus, our data provided strong evidence that HDGF was highly secreted by MSCs.

### 3.3. HDGF Contributed to the Protective Effects of MSC-CdM

**Figure 3:**
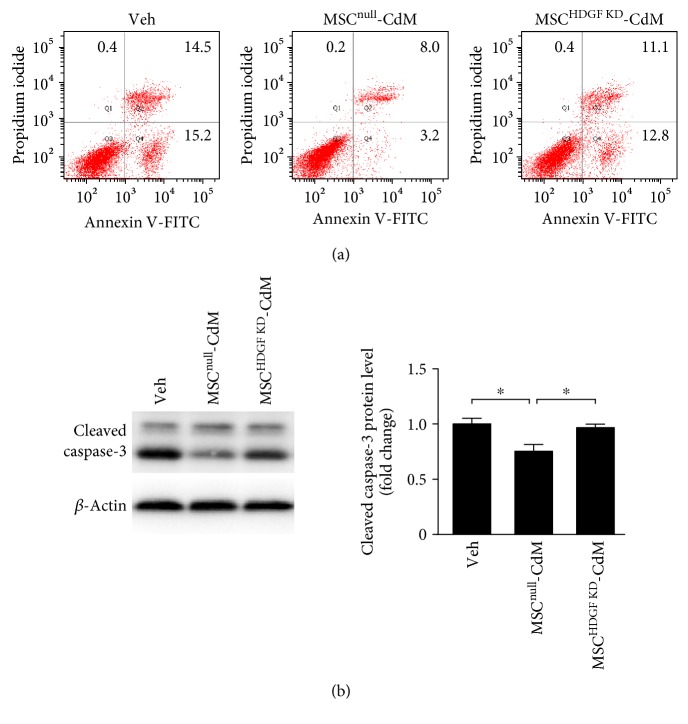
HDGF contributed to the protective effects of MSC-CdM. Conditioned medium that collected from MSCs transfected with vector (MSC^null^-CdM) or HDGF shRNA lentivirus (MSC^HDGF KD^-CdM) were treated to H9C2 cells subjected to 9 h of hypoxia followed by 4 h of reoxygenation. (a) Cell death was evaluated with flow cytometry analysis. Annexin V−/PI−: viable cells; Annexin V+/PI−: early apoptotic cells; Annexin V+/PI+: late apoptotic or necrotic cells; Annexin V−/PI+: necrotic cells. (b) Cleaved caspases-3 as detected by Western blotting. Data represent mean ± SEM of values from three determinations. ^∗^*P* < 0.05.

To test whether HDGF was involved in protective effects of MSC-CdM, we first used lentiviral shRNA to knockdown HDGF in MSCs and investigated apoptosis in heart-derived H9C2 cells subjected to 4 h of reoxygenation following 9 h of hypoxia. PI-Annexin V double staining was used to identify the prevention effects of MSC-CdM (Figure 3(a)). Conditioned medium derived from MSCs that transfected with empty vector (MSC^null^) reduced population of PI-Annexin V double positive cells by 45.7% after reoxygenation injury, which was absent when treated with the conditioned medium derived from MSCs that transfected shRNA lentivirus targeting HDGF. Being consistent with PI-Annexin V double staining, MSC^null^-CdM significantly attenuated (*P* < 0.05) reoxygenation-induced activation of caspase-3 in H9C2 cells (Figure 3(b)). In contrast, knockdown of HDGF in MSCs impaired (*P* < 0.05) the MSC-CdM-mediated inhibitive effects on caspase-3 activation.

### 3.4. HDGF Reduced Apoptosis and Activated PKC*ε* Pathway

**Figure 4:**
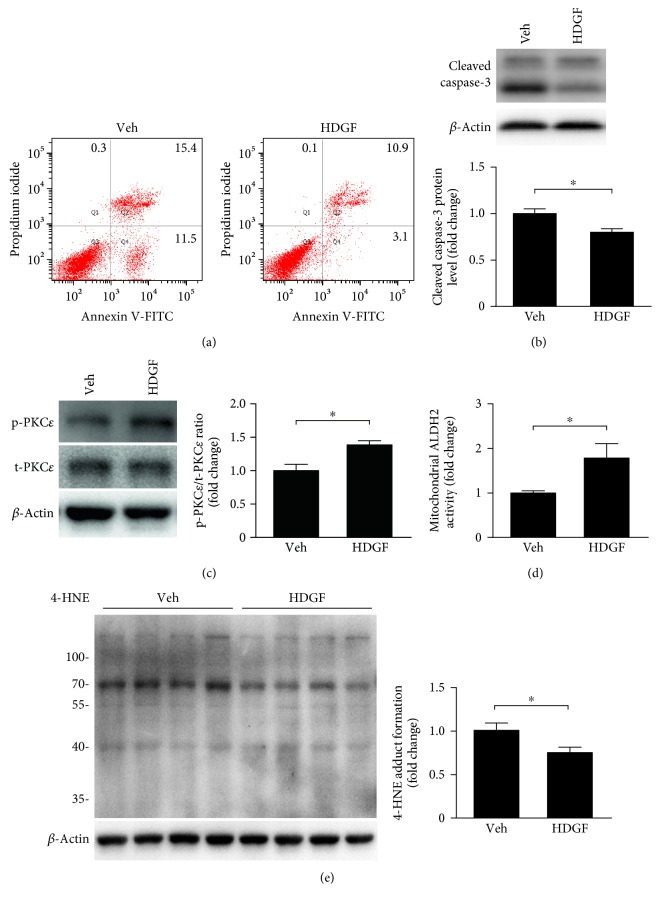
HDGF reduced apoptosis and activated PKC*ε* pathway. H9C2 cells treated by recombinant mouse HDGF (100 nmol/L) or vehicle control were subjected to 9 h of hypoxia followed by 4 h of reoxygenation. (a) Cell death was evaluated with flow cytometry analysis. (b) Cleaved caspases-3 as detected by Western blotting. Data represent mean ± SEM of values from three determinations. (c) Phosphorylation of PKC*ε* as detected by Western blotting. Data represent mean ± SEM of values from three determinations. (d) Mitochondria were isolated from H9C2 cells and activity of ALDH2 in mitochondria was measured. Data represent mean ± SEM of values from three mice. (e) 4-HNE protein adducts in H9C2 cells were assessed. Data represent mean ± SEM of values from four determinations. ^∗^*P* < 0.05.

To further validate the direct protective effects of HDGF on reperfusion injury, we applied mouse HDGF recombinant protein (100 nmol/L, Novoprotein Scientific, NJ, USA) or PBS as vehicle (Veh) to H9C2 cells that were subjected to H/R injury. PI-Annexin V double positive population was reduced by 29.2% when treated with HDGF recombinant protein (Figure 4(a)). In addition, a decrease in percentage of Annexin V positive and low PI cell population (in Q4 quadrant) was observed, suggesting that HDGF protected against reperfusion-induced early stage apoptosis. We also performed immunoblotting assay to detect the activation of caspase-3 and found that cleaved caspase-3 was significantly reduced in the HDGF group, compared to the control (Figure 4(b)).

To explore the intracellular mechanisms underlying the protective effects of HDGF, we examined PKC*ε* activity as this pathway has been proved to play essential roles in myocardial preconditioning and cytoprotection [20–23]. Our data showed that HDGF induced PKC*ε* phosphorylation (Figure 4(c)).Phosphorylated PKC*ε* has been shown to translocate into mitochondria and interacts with ALDH2 contributing to 4-HNE detoxification during reperfusion injury [24]. Therefore, we assessed ALDH2 activity in myocardial mitochondria and 4-HNE which could reflect whether PKC*ε* has been activated by HDGF. Our data showed that the delivery of recombinant HDGF significantly enhanced the activity of ALDH2 in mitochondria (*P* < 0.05) (Figure 4(d)) and prevented 4-HNE accumulation (Figure 4(e)), compared with the control group. Thus, these data support the notion that HDGF reduced reoxygenation-induced oxidative stress through PKC*ε* activation.

### 3.5. PKC*ε* Played a Key Role in HDGF-Mediated Protection against Reperfusion Injury

**Figure 5:**
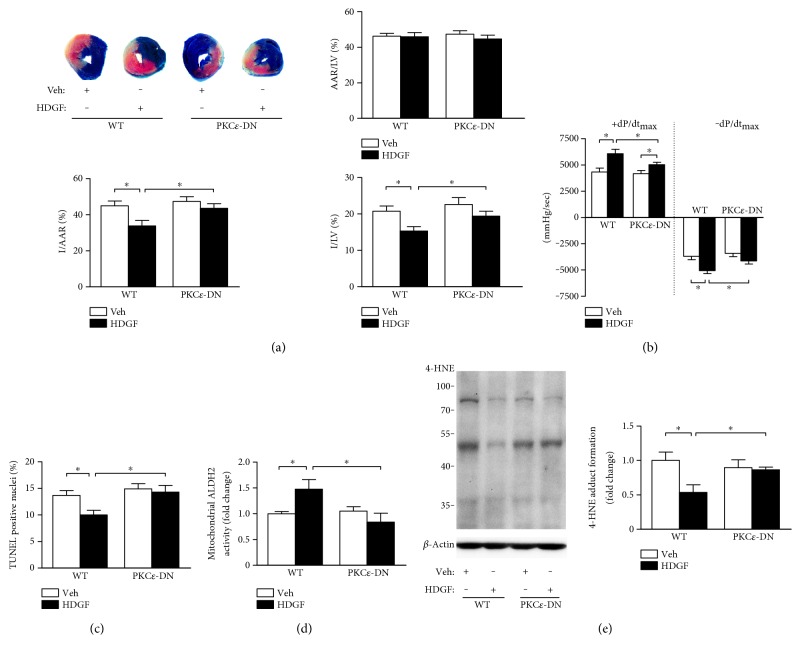
PKC*ε* contributed to HDGF-induced reduction of reperfusion injury. PKC*ε* dominant negative mice (PKC*ε*-DN) and wild-type (WT) littermates with or without 50 *μ*g/kg recombinant mouse HDGF treatment intramyocardially were subjected to 45 min of cardiac ischemia followed by 24 h reperfusion. (a) Ratio of area at risk to left ventricle area (AAR/LV), ratio infarct size to AAR ratio (I/AAR), and ratio of infarct size to LV area (I/LV) of hearts were assessed. Data represent mean ± SEM of values from five mice. (b) The maximum rates of rise and decline of left-ventricular pressure (+dp/dt_max_ and −dp/dt_max_) assessed at 24 h reperfusion. Data are mean ± SEM of values from six mice. (c) Quantitative analysis of TUNEL positive cells at 24 h reperfusion. Data are mean ± SEM of values from three hearts per group, with at least 3000 nuclei examined per heart. (d) Mitochondria were isolated from heart tissue after reperfusion injury and the activity of ALDH2 in mitochondria was measured. Data represent mean ± SEM of values from three mice. (e) 4-HNE protein adducts in heart tissues was assessed. Data represent mean ± SEM of values from three mice. ^∗^*P* < 0.05.

To further determine the role of PKC*ε* in HDGF-mediated protection *in vivo*, recombinant mouse HDGF (50 *μ*g/kg) or PBS (as vehicle) was administered intramyocardially to both PKC*ε*-DN mice and WT littermate (as controls). Administration of recombinant HDGF reduced I/AAR to 33.8 ± 3.1% (*P* < 0.05), compared to 44.9 ± 2.6% in the control group (Figure 5(a)). However, this reduction in infarct size by HDGF delivery was absent in PKC*ε*-DN mice that had similar I/AAR to those without HDGF intervention, although the I/AAR was similar between PKC*ε*-DN and WT mice, and AAR/LV in all groups had no significant difference. In addition, recombinant HDGF markedly increased +dp/dt_max_ and −dp/dt_max_ at 24 h reperfusion in WT mice (*P* < 0.05) (Figure 5(b) and Supplementary Table 2). Although the mean value of +dp/dt_max_ and −dp/dt_max_ in PKC*ε*-DN mice was increased by HDGF treatment, the HDGF-mediated improvement of hemodynamic performance was significantly limited compared to that in WT (*P* < 0.05).

Being consistent with the infarct size quantification, administration of recombinant HDGF significantly decreased TUNEL positive myocytes (*P* < 0.05) in the peri-infarct area in WT mice, which was not observed in PKC*ε*-DN mice (Figure 5(c)). Meanwhile, we detected an enhanced mitochondrial ALDH2 activity (Figure 5(d)) and a significant reduction in 4-HNE accumulation (Figure 5(e)) in reperfusion-injured myocardium of WT mice, but not in that of PKC*ε*-DN mice.

Moreover, HDGF enhanced contractile performance (Supplementary Fig. S3A, B) and upregulated calcium transient amplitude (Supplementary Fig. S3C, D) in myocytes isolated from both WT and PKC*ε*-DN mice. This set of data could account for the improved hemodynamic performance in HDGF-treated PKC*ε*-DN mice but also imply that additional mechanisms might be involved in HDGF-induced function recovery.

## 4. Discussion

In the present study, we have demonstrated that the conditioned medium derived from MSCs could exert cardioprotection. Through an iTRAQ-based proteomic analysis of the secretion from MSCs and CFs, we identified HDGF as an important component that played an essential role in the prosurvival effects offered by MSC therapy. Administration of recombinant HDGF alone recapitulated MSC-mediated protection, resulting in a reduction in infarct size, decreased apoptosis, and improved cardiac function through PKC*ε* pathway.

Although CFs secrete paracrine factors such as FGF-1, FGF-2, IL-33, and tissue inhibitory metalloproteinases (TIMPs) that are beneficial to cardioprotection [25, 26], limited effects of CF-CdM were observed in our study. MSCs have been reported to secrete some distinct cytokines compared to dermal fibroblasts, which allow MSC therapy to exhibit superiority over fibroblasts therapy in the wound healing process [27, 28]. As MSCs hold great promise for cell-based therapy, the identification of secreted factors along with the related underlying mechanisms is of great biological and therapeutic importance. We, for the first time, applied iTRAQ-based proteomics analysis to compare the secretion from MSCs and CFs. A list of secreted factors specifically highly expressed by MSCs was defined, among which HDGF was further investigated and proved to induce myocardial protection. Thus, we provided a feasible approach to identify protective factors in the secretions from MSCs. Of note, hypoxia can improve the paracrine effects of different types of cells. The conditioned medium from hypoxia-preconditioned CFs was reported to be able to induce protection for reperfusion-injured myocardium [29], which was not observed in our study when the conditioned medium was collected under normoxic condition. Our previous studies demonstrated that hypoxia preconditioning enhanced biological function and cardioprotective effects of MSCs in rodent and primate models of permanent myocardial ischemia [10, 11]. Therefore, it remains to be further investigated whether hypoxic preconditioning enhances MSC protection against reperfusion injury and triggers MSC secretome alterations.

PKC*ε* is one of the central players in protection induced by ischemic preconditioning [20, 21], which is considered as the most efficient way to prevent reperfusion injury [30]. Activation of PKC*ε* induces its translocation to the mitochondria and triggers a variety of mechanisms to induce antiapoptotic and antinecrotic effects, including enhancement of ALDH2 activity which detoxifies ROS-generated 4-HNE [20, 24], interaction with cytochrome *c* oxidase subunit IV [31], inhibition of mitochondrial permeability transition pore (mPTP) opening [32], and stabilization of mitochondrial membrane potential (Δ*ψ*m). PKC*ε* is known to be activated during reperfusion injury. However, the extent of PKC*ε* activation by reperfusion may be insufficient to induce significant cardioprotection, since there is no difference in cardiac function and infarct sizes between WT and PKC*ε* knockout mice [33, 34]. Further deterioration was neither observed in PKC*ε*-DN mice in our study. On the other hand, deletion of PKC*ε* can abolish ischemic preconditioning-mediated protection [34, 35], indicating that PKC*ε* is indispensable and might be further enhanced to take part in the protection against reperfusion injury. In this regard, identifying agents, such as HDGF that stimulates the PKC*ε* pathway, will be of therapeutic benefit. Our data indicates that HDGF activates PKC*ε* and induces a PKC*ε*-dependent protection, including suppression of apoptosis, limitation of reperfusion-induced oxidative stress, and reduction in infarct size.

It has to be born in mind though that PKC*ε* may not be the sole pathway involved in HDGF-mediated protection, since HDGF-induced effects on myocyte contractility and calcium handling were not impaired when PKC*ε* was disrupted. In addition, we utilized isolated adult cardiomyocytes to detect myocyte calcium cycling and contractility in the present study. The H9C2 cell line was also used, due to the ease of handling as well as the ethical issues of laboratory animal use without significantly compromising the mechanistic molecular experiment. However, it is a cloned embryonic cardiac myoblast cell line obtained from embryonic rat heart, which does not truly possess morphological characteristics of matured cardiomyocytes.

HDGF was originally isolated from the conditioned medium of hepatoma-derived cells as a heparin-binding growth factor, and its role in the development of cardiovascular tissues was proved afterwards [36]. Over the last two decades, HDGF has been reported to be involved in many biological processes, such as wound repair [37], angiogenesis [38], and antiapoptosis [39]. Downregulation of HDGF impairs activation of certain survival pathways, leading to the cellular apoptosis under stress [18, 39]. Therefore, HDGF may function as an antiapoptotic factor underlying the protection of MSCs. In this study, knockdown of HDGF impaired the effects of MSCs, indicating that HDGF plays an important role in MSC protection. HDGF also can improve proliferation [16] and migration [40], both of which are important processes in cardiac repair and regeneration. Administration of recombinant HDGF has been shown to induce a reduction in infarct size and improved cardiac function, suggesting that HDGF can be of great clinical benefit in the prevention of reperfusion injury.

## Supplementary Material

Supplemental Information.

## Figures and Tables

**Table 1:** Functional classification of the highly secreted protein identified in MSC-CdM.

GO classification	Gene	Protein
ADP catabolic process	NUDT9	ADP-ribose pyrophosphatase, mitochondrial

Apoptotic process	HINT1	Histidine triad nucleotide-binding protein 1
	NME1	Nucleoside diphosphate kinase A
	NME2	Nucleoside diphosphate kinase B

Biological process	OAF	Out at first protein homolog

Biosynthetic process	HRT1	Hypoxanthine-guanine phosphoribosyltransferase
	ADA	Adenosine deaminase
	EEF1A1	Elongation factor 1-alpha 1
	TPI1	Triosephosphate isomerase
	PGAM1	Phosphoglycerate mutase 1
	EIF2S3X	Eukaryotic translation initiation factor 2 subunit 3, X-linked

Catabolic process	GSTO1	Glutathione S-transferase omega-1

Cell adhesion	SPP1	Osteopontin

Cell cycle	PIN4	Peptidyl-prolyl cis-trans isomerase NIMA-interacting 4

Cell differentiation	TPT1	Translationally controlled tumor protein
	STMN1	Stathmin

Cell growth	MTPN	Myotrophin

Cell morphogenesis	SMARCA4	Transcription activator BRG1

Cell motility	BRK1	Protein BRICK1

Cell-cell signaling	HDGF	Hepatoma-derived growth factor

Cellular component morphogenesis	CFL1	Cofilin-1
	TUBA4A	Tubulin alpha-4A chain
	SAA3	Serum amyloid A-3 protein

Cellular process	SOD3	Extracellular superoxide dismutase [Cu-Zn]
	LCN2	Neutrophil gelatinase-associated lipocalin
	NAMPT	Nicotinamide phosphoribosyltransferase
	UCHL3	Ubiquitin carboxyl-terminal hydrolase isozyme L3

DNA replication	PCNA	Proliferating cell nuclear antigen

Endothelial cell proliferation	HMGB1	High mobility group protein B1

Fatty acid catabolic process	ACOT7	Cytosolic acyl coenzyme A thioester hydrolase

Glycolysis	LDHA	L-lactate dehydrogenase A chain
	PGK1	Phosphoglycerate kinase 1
	GAPDH	Glyceraldehyde-3-phosphate dehydrogenase
	ENO1	Alpha-enolase

G-protein coupled receptor signaling pathway	CXCL5	C-X-C motif chemokine 5
	CCL8	C-C motif chemokine 8

Immune system process	HMGB2	High mobility group protein B2
	MIF	Macrophage migration inhibitory factor
	PSMA1	Proteasome subunit alpha type-1

Metabolic process	PKM	Pyruvate kinase PKM

Monosaccharide metabolic process	GALM	Aldose 1-epimerase

Oxidation-reduction process	AKR1B1	Aldose reductase
	AKR1B8	Aldose reductase-related protein 2

Pentose phosphate shunt	PGLS	6-phosphogluconolactonase

Protein folding	HSP90AA1	Heat shock protein HSP 90-alpha
	HSP90AB1	Heat shock protein HSP 90-beta
	ST13	Hsc70-interacting protein

Protein metabolic process	LAP3	Cytosol aminopeptidase

Regulation of biological process	IGFBP6	Insulin-like growth factor-binding protein 6

Response to oxidative stress	PRDX5	Peroxiredoxin-5, mitochondrial

RNA splicing	PTBP1	Polypyrimidine tract-binding protein 1
	PCBP1	Poly(rC)-binding protein 1
	SFPQ	Splicing factor, proline- and glutamine-rich

Translation	RPS20	40S ribosomal protein S20

Carbohydrate metabolic process	GLO1	Lactoylglutathione lyase

GO: Gene Ontology.

## Abbreviations

HDGF: Hepatoma-derived growth factor
MSC: Mesenchymal stem cell
CF: Cardiac fibroblast
CdM: Conditioned medium
PKC*ε:*: Protein kinase C epsilon
WT: Wild type
PKC*ε*-DN: PKC*ε* dominant negative
4-HNE: 4-hydroxy-2-nonenal
ALDH2: Aldehyde dehydrogenase family 2
KD: Knockdown.

## References

[1] Roger V. L., Go A. S., Lloyd-Jones D. M. (2012). Heart disease and stroke statistics—2012 update: a report from the American Heart Association. *Circulation*.

[2] Wu L., Tan J. L., Wang Z. H. (2015). ROS generated during early reperfusion contribute to intermittent hypobaric hypoxia-afforded cardioprotection against postischemia-induced Ca^2+^ overload and contractile dysfunction via the JAK2/STAT3 pathway. *Journal of Molecular and Cellular Cardiology*.

[3] Lopez-Bernardo E., Anedda A., Sanchez-Perez P., Acosta-Iborra B., Cadenas S. (2015). 4-Hydroxynonenal induces Nrf2-mediated UCP3 upregulation in mouse cardiomyocytes. *Free Radical Biology & Medicine*.

[4] Awasthi Y. C., Ramana K. V., Chaudhary P., Srivastava S. K., Awasthi S. (2017). Regulatory roles of glutathione-S-transferases and 4-hydroxynonenal in stress-mediated signaling and toxicity. *Free Radical Biology & Medicine*.

[5] Eaton P., Li J. M., Hearse D. J., Shattock M. J. (1999). Formation of 4-hydroxy-2-nonenal-modified proteins in ischemic rat heart. *The American Journal of Physiology*.

[6] Ji S. T., Kim H., Yun J., Chung J. S., Kwon S. M. (2017). Promising therapeutic strategies for mesenchymal stem cell-based cardiovascular regeneration: from cell priming to tissue engineering. *Stem Cells International*.

[7] Tamama K., Barbeau D. J. (2012). Early growth response genes signaling supports strong paracrine capability of mesenchymal stem cells. *Stem Cells International*.

[8] Hu X., Chen P., Wu Y. (2016). MiR-211/STAT5A signaling modulates migration of mesenchymal stem cells to improve its therapeutic efficacy. *Stem Cells*.

[9] Hu X., Zhang L., Jin J. (2015). Heparanase released from mesenchymal stem cells activates integrin beta1/HIF-2alpha/Flk-1 signaling and promotes endothelial cell migration and angiogenesis. *Stem Cells*.

[10] Hu X., Xu Y., Zhong Z. (2016). A large-scale investigation of hypoxia-preconditioned allogeneic mesenchymal stem cells for myocardial repair in nonhuman primates: paracrine activity without remuscularization. *Circulation Research*.

[11] Chen P., Wu R., Zhu W. (2014). Hypoxia preconditioned mesenchymal stem cells prevent cardiac fibroblast activation and collagen production via leptin. *PLoS One*.

[12] Hu X., Wu R., Jiang Z. (2014). Leptin signaling is required for augmented therapeutic properties of mesenchymal stem cells conferred by hypoxia preconditioning. *Stem Cells*.

[13] Timmers L., Lim S. K., Hoefer I. E. (2011). Human mesenchymal stem cell-conditioned medium improves cardiac function following myocardial infarction. *Stem Cell Research*.

[14] Timmers L., Lim S. K., Arslan F. (2007). Reduction of myocardial infarct size by human mesenchymal stem cell conditioned medium. *Stem Cell Research*.

[15] Baines C. P., Zhang J., Wang G.-W. (2002). Mitochondrial PKCε and MAPK form signaling modules in the murine heart: enhanced mitochondrial PKCε-MAPK interactions and differential MAPK activation in PKCε-induced cardioprotection. *Circulation Research*.

[16] Li M., Shen J., Wu X. (2013). Downregulated expression of hepatoma-derived growth factor (HDGF) reduces gallbladder cancer cell proliferation and invasion. *Medical Oncology*.

[17] Kishima Y., Yoshida K., Enomoto H. (2002). Antisense oligonucleotides of hepatoma-derived growth factor (HDGF) suppress the proliferation of hepatoma cells. *Hepato-Gastroenterology*.

[18] Hsu S. S., Chen C. H., Liu G. S. (2012). Tumorigenesis and prognostic role of hepatoma-derived growth factor in human gliomas. *Journal of Neuro-Oncology*.

[19] Yu Y., Shen H., Yu H. (2011). Systematic proteomic analysis of human hepotacellular carcinoma cells reveals molecular pathways and networks involved in metastasis. *Molecular BioSystems*.

[20] Budas G. R., Churchill E. N., Mochly-Rosen D. (2007). Cardioprotective mechanisms of PKC isozyme-selective activators and inhibitors in the treatment of ischemia-reperfusion injury. *Pharmacological Research*.

[21] Tong H., Chen W., Steenbergen C., Murphy E. (2000). Ischemic preconditioning activates phosphatidylinositol-3-kinase upstream of protein kinase C. *Circulation Research*.

[22] Gray M. O., Karliner J. S., Mochly-Rosen D. (1997). A selective ε-protein kinase C antagonist inhibits protection of cardiac myocytes from hypoxia-induced cell death. *Journal of Biological Chemistry*.

[23] Liu G. S., Cohen M. V., Mochly-Rosen D., Downey J. M. (1999). Protein kinase C- ξ is responsible for the protection of preconditioning in rabbit cardiomyocytes. *Journal of Molecular and Cellular Cardiology*.

[24] Budas G. R., Churchill E. N., Disatnik M.-H., Sun L., Mochly-Rosen D. (2010). Mitochondrial import of PKCε is mediated by HSP90: a role in cardioprotection from ischaemia and reperfusion injury. *Cardiovascular Research*.

[25] Seki K., Sanada S., Kudinova A. Y. (2009). Interleukin-33 prevents apoptosis and improves survival after experimental myocardial infarction through ST2 signaling. *Circulation Heart Failure*.

[26] Jiang Z. S., Wen G. B., Tang Z. H., Srisakuldee W., Fandrich R. R., Kardami E. (2009). High molecular weight FGF-2 promotes postconditioning-like cardioprotection linked to activation of protein kinase C isoforms, as well as Akt and p70 S6 kinases. *Canadian Journal of Physiology and Pharmacology*.

[27] Chen L., Tredget E. E., PY W., Wu Y. (2008). Paracrine factors of mesenchymal stem cells recruit macrophages and endothelial lineage cells and enhance wound healing. *PLoS One*.

[28] Wang S., Yang H., Tang Z., Long G., Huang W. (2016). Wound dressing model of human umbilical cord mesenchymal stem cells-alginates complex promotes skin wound healing by paracrine signaling. *Stem Cells International*.

[29] Abrial M., Da Silva C. C., Pillot B. (2014). Cardiac fibroblasts protect cardiomyocytes against lethal ischemia-reperfusion injury. *Journal of Molecular and Cellular Cardiology*.

[30] Murry C. E., Jennings R. B., Reimer K. A. (1986). Preconditioning with ischemia: a delay of lethal cell injury in ischemic myocardium. *Circulation*.

[31] Ogbi M., Johnson J. A. (2006). Protein kinase Cε interacts with cytochrome c oxidase subunit IV and enhances cytochrome *c* oxidase activity in neonatal cardiac myocyte preconditioning. *The Biochemical Journal*.

[32] Baines C. P., Song C. X., Zheng Y. T. (2003). Protein kinase Cε interacts with and inhibits the permeability transition pore in cardiac mitochondria. *Circulation Research*.

[33] Gray M. O., Zhou H. Z., Schafhalter-Zoppoth I., Zhu P., Mochly-Rosen D., Messing R. O. (2004). Preservation of base-line hemodynamic function and loss of inducible cardioprotection in adult mice lacking protein kinase C*ε*. *The Journal of Biological Chemistry*.

[34] Saurin A. T., Pennington D. J., Raat N. J., Latchman D. S., Owen M. J., Marber M. S. (2002). Targeted disruption of the protein kinase C epsilon gene abolishes the infarct size reduction that follows ischaemic preconditioning of isolated buffer-perfused mouse hearts. *Cardiovascular Research*.

[35] Ping P., Takano H., Zhang J. (1999). Isoform-selective activation of protein kinase C by nitric oxide in the heart of conscious rabbits: a signaling mechanism for both nitric oxide-induced and ischemia-induced preconditioning. *Circulation Research*.

[36] Everett A. D. (2001). Identification, cloning, and developmental expression of hepatoma-derived growth factor in the developing rat heart. *Developmental Dynamics*.

[37] Everett A. D., Lobe D. R., Matsumura M. E., Nakamura H., McNamara C. A. (2000). Hepatoma-derived growth factor stimulates smooth muscle cell growth and is expressed in vascular development. *Journal of Clinical Investigation*.

[38] Everett A. D., Narron J. V., Stoops T., Nakamura H., Tucker A. (2004). Hepatoma-derived growth factor is a pulmonary endothelial cell-expressed angiogenic factor. *American Journal of Physiology Lung Cellular and Molecular Physiology*.

[39] Tsang T. Y., Tang W. Y., Tsang W. P., Co N. N., Kong S. K., Kwok T. T. (2008). Downregulation of hepatoma-derived growth factor activates the Bad-mediated apoptotic pathway in human cancer cells. *Apoptosis*.

[40] Wang C. H., Davamani F., Sue S. C. (2011). Cell surface heparan sulfates mediate internalization of the PWWP/HATH domain of HDGF via macropinocytosis to fine-tune cell signalling processes involved in fibroblast cell migration. *Biochemical Journal*.

