# Supplementary material for: Hepatoma-Derived Growth Factor Secreted from Mesenchymal Stem Cells Reduces Myocardial Ischemia-Reperfusion Injury
Source: Stem Cells Int. 2017 Nov 14;2017:1096980. doi: 10.1155/2017/1096980 (PMC5735317; doi:10.1155/2017/1096980)
Supplement: Supplementary file 1 — Supplemental Information. [file 1096980.f1.pdf]

Supplemental Information

Materials and Methods

Animal

Wild-type (WT) littermates and PKCε dominant negative (PKCε-DN) mice were obtained from Prof. Peipei Ping as a gift[1]. The animals were fed a standard laboratory diet with free access to food and water, and kept in an enviroment with controlled room temperature, humidity (65%–70%), and a 12:12 h light/dark cycle. Male mice 8-12 weeks of age were used for the study. All animal studies were performed with the approval of the Institutional Animal Care and Use Committee, Zhejiang University and according to the Chinese Guideline for Laboratory Animal Care and Use.

In Vivo Models

The mice were anesthetized with intraperitoneal injection of pentobarbital sodium (60 mg/kg). A rodent ventilator was used. Myocardium ischemia was achieved by ligating the left anterior descending (LAD) coronary artery including silicon tubing on top of the coronary artery with an 8-0 Prolene suture. Fifteen minutes before occlusion, 0.2 mL of vechile or MSC-CdM or CF-CdM was injected via tail vein. After 45 min of ischemia, silicon tubing was removed to achieve reperfusion. The mice were sacrificed at various times for different experiments.

Hypoxia/Reoxygenation

Cells were subjected to hypoxia and reoxygenation with a buffer simulating the ischemic milieu containing 128 mM NaCl, 2.2 mM $NaHCO_3$, 14.8 mM KCl, 1.2 mM $MgSO_4$, 1.2 mM K_2HPO_4, 1 mM $CaCl_2$, and 10 mM sodium-lactate (pH 6.4) in a hypoxic chamber containing 95% N_2/5% CO_2. After this, the cells were placed in a standard incubator in a normal medium.

Isolation of Mesenchymal Stem Cells

Mice MSCs were isolated from 4- to 5-week-old WT mice bone marrow, characterized for their surface antigen profiling by flow cytometry and cultured as described previously[2]. Briefly, bone marrows from three mice were plated in a 10-cm dish in DMEM/F12 (Invitrogen, CA, USA) supplemented with 20% bovine serum albumin and antibiotics. After 3-5 passages, the cells were characterized for their surface antigen profiling by flow cytometry. The cells (1×10^6 cells/100 μL) were resuspended in phosphate-buffered saline (PBS) with 1% FBS for each antibody and incubated with mouse-specific antibodies fluorescein isothiocyanate (FITC)-CD34, phycoerythrin (PE)-CD105 and PE-CD166 , APC- CD45 and PE-CD73 (all from BD Biosciences, CA, USA). After 45 min at 4°C, the cells were washed twice in PBS, and at least 10,000 events were acquired for each sample. The cells expressed the MSC markers CD105, CD73, and CD90 and were negative for the markers CD34, CD31 and CD45.

Isolation of Cardiac Fibroblasts

Cardiac fibroblasts were isolated from WT mice and characterized as described previously[3]. Briefly, the ventricles of hearts were minced and digested in 0.05% collagenase and 0.05% pancreatin (Gibco, Invitrogen, CA, USA) containing PBS solution at 37°C and waved 4–5 times at 220 rpm for 10 min. The cells were pre-plated for 1-h on uncoated culture dishes (Corning Inc., NY, USA), during which CFs rapidly adhered to the dishes. Fibroblasts up to passage three were identified by fibroblast specific protein 1 (FSP-1), vimentin, α-SMA, CD31 and Troponin T immunofluorescence staining.

Conditioned Media Preparation

MSCs or fibroblasts of 80% confluence were washed with PBS and cultured in serum-free medium for 24 h for collection. The collected conditioned medium was cleared by centrifugation, and concentrated 25-fold less of the original volume using 3 kDa molecular weight cutoff ultrafiltration membranes (Millipore, MA, USA). The concentrated medium was diluted with PBS and re-concentrated for three times to desalt the medium, and then sterilizing though 220 nm filters. The protein concentration of the final solution was measured using a protein assay kit (Bio-Rad, CA, USA) and diluted with PBS to 0.5 mg/mL for injection.

Determination of Myocardial Infarct Size

After 24 h of reperfusion, the mice were anesthetized with an intraperitoneal injection of pentobarbital sodium (60 mg/kg). Hearts were processed by LAD ligation at the same location as before and were subjected to perfusion with 2% Evan's blue dye via the aorta using a catheter. Then, 1-mm-thick sections of hearts were made along the long axis and incubated in 1% 2,3,5-triphenyltetrazolium chloride (Sigma-Aldrich) for 5 min at 37°C. Myocardial slices were assessed by a blinded observer using Image-Pro Plus (Media Cybernetics, lnc, MD, USA).

Mitochondria Isolation

Mitochondria were isolated using a mitochondria protein extraction kit (Keygentec, Nanjing, China) according to the instruction supplied from the manufacturer. Briefly, cultured cardiomyocytes or minced heart tissues were homogenated in ice-cold Lysis Buffer 1 and centrifuged at 800 *g* at 4°C. The supernatants were moved into Medium Buffer, and centrifuged at 15,000 *g* at 4°C. The pellet was mitochondria.

Western Blot Analysis

Proteins were extracted from cells or heart tissues. 40 μg protein for each sample was electrophoresed on a SDS-gel and transferred onto a PVDF membranes (Bio-Rad), blocked with 5% skim milk for 1 hour at room temperature, and incubated overnight at 4°C with primary antibodies against phosphorylated PKCε (1:500, Santa Cruz, CA, USA), PKCε, cleaved caspase-3, β-actin (1:1000, all from Cell Signaling Technology, Danvers, MA, USA), 4HNE (1:500) (both from Abcam, Cambridge, MA, USA). HRP-conjugated secondary antibodies were applied to incubate the membranes at room temperature for 1 hour. Then membranes were exposed with Chemiluminescence Detection Kit (Millipore).

Aldehyde Dehydrogenase Family 2 (ALDH2) Activity Assay

The ALDH2 activity of mitochondria from cardiomyocytes was measured using a mito-ALDH2 activity kit (Abcam) according to the instruction from the manufacturer. Briefly, mitochondria that were isolated from cardiomyocytes were pelleted and solubilized in Extraction Buffer. The samples were incubated in a 96-well plate and reacted with Activity Solution according to the reaction: acetaldehyde + NAD+ → acid + NADH. The

activity of ALDH2 was measured using SpectraMax 340PC384 Microplate Reader (Molecular Devices, LLC., CA, USA) monitoring the absorbance at 450 nm.

Transferase dUTP nick end labeling (TUNEL) Assay and Immunofluorescence Staining

Heart tissues obtained 24 h after surgery were dehydrated in 30% sucrose solution and embedded in Tissue-Tek OCT Compound and snap frozen in liquid nitrogen. For immunofluorescence, frozen tissue sections (6.0 μm thick) were fixed in 4% paraformaldehyde, permeabilized with 0.2% Triton X-100, and blocked with 5% goat serum. Incubation was performed with primary antibody and respective secondary antibodies. The apoptosis of cells was detected in situ with the use of TUNEL (Roche Applied Science, IN, USA). DAPI was used to stain nuclei, while cTnI antibody (1:200 Abcam) was applied as cardiomyocytes marker. Sections were examined under a fluorescent microscope.

Flow Cytometry Analysis of Cell Apoptosis

The Annexin V-FITC/PI Apoptosis Detection Kit was used to evaluate apoptosis of cells. After being rinsed with ice cold PBS, the cells were resuspended in 200 μl of binding buffer. Ten microliters of Annexin V stock solutions was added to the cells and incubated for 30 min at 4°C. The cells were then incubated with 5 ml propidium iodide (PI) and immediately analyzed with a FACScan. Ten thousands events were acquired on a FACSC-LSR (Becton-Dickinson, San Jose, CA) and analyzed with CellQuest (Becton-Dickinson) software.

Hemodynamics Assessment

Hemodynamic assessment was taken at24-hour reperfusion by a 1.4 F pressure catheter inserted into the left ventricle (LV) through the right carotid artery. The transducer was connected to Powerlab system (AD Instruments, Castle Hill, Australia). LV pressure and LV maximum ± dp/dt were recorded and averaged from 15 beats.

Lentivirus Construction and Infection

Construction of the recombinant lentivirus with HDGF was performed by Genechem Company. For MSCs infection, cells were seeded at a density of $1x10^5$ cells in a six-well plate and infected with lentiviral vectors

with 10 mg/ml polybrene (Millipore, Boston, MA, USA). At 12-hour post-infection, the medium was replaced. Forty-eight hours later, the transfected cells were cultured in a 5% CO_2-humidified incubator at 37°C.

Real Time Quantitative Polymerase Chain Reaction (RT-qPCR) Analysis

Primers for amplification of mouse HDGF genes were used to determine the expression of HDGF in fibroblasts and MSCs. The amplification program consisted of initial denaturation at 95 °C for 10 min followed by 40 cycles from 92 °C for 15 s, annealing at 60 °C for 30 s and extension at 72 °C for 15 s. To minimize the effects of unequal quantities of starting RNA and eliminate potential sources of inconsistency, the relative expression levels of each gene was normalized to β-actin using the 2-ΔΔCt method. Control experiments used no reverse transcriptase.

Protein Digestion and iTRAQ Labeling

For each sample, proteins were precipitated with ice-cold acetone, and then were redissolved in the dissolution buffer (0.5 M triethylammonium bicarbonate, 0.1% SDS). Then proteins were quantified by BCA protein assay, and 100 μg of protein was tryptically digested and the resultant peptide mixture was labeled using chemicals from the iTRAQ reagent kit (Applied Biosystems, California, USA). Disulfide bonds were reduced in 5 mM Tris-(2-carboxyethy) phosphine (TCEP) for1 h at 60℃, followed by blocking cysteine residues in 10 mM methylmethanethiosulfonate (MMTS) for 30 min at room temperature, before digestion with sequence-grade modified trypsin (Promega, Madison, WI, USA). For labeling, each iTRAQ reagent was dissolved in 50 μL of isopropanol and added to the respective peptide mixture. The labeled samples were combined and dried.

High pH reverse phase separation

The peptide mixture was redissovled in the buffer A (buffer A: 20mM ammonium formate in water, pH10.0, adjusted with ammonium hydroxide), and then fractionated by high pH separation using a Aquity UPLC system (Waters Corporation, Milford, MA, USA) connected to a reverse phase column (ACQUITY UPLC Peptide C18 column, 2.1mm x 150 mm, 1.7μm, 130 Å, Waters Corporation, Milford, MA, USA). High pH separation was performed using a linear gradient. Starting from 5% B to 35% B in 25 min (B: 20mM ammonium formate in 90% ACN, pH 10.0, adjusted with ammonium hydroxide). The column was re-equilibrated at initial conditions for 15 min. The column flow rate was maintained at 600μL/min and column temperature was maintained at room temperature. Collected fractions were dried in a vacuum concentrator for the next step.

Low pH nano-HPLC-MS/MS analysis

The mixed peptides were separated by nano-HPLC (Eksigent Technologies, Dublin, CA, USA) on the secondary RP analytical column (Eksigent, C18, 3µm, 150 mm x 75µm). Peptides were subsequently eluted using the following gradient conditions with phase B (98% ACN with 0.1% formic acid) from5 to 45%B (5-70 min) and total flow rate was maintained at 300 nL/min. Electrospray voltage of 2.3 kV versus the inlet of the mass spectrometer was used.

Triple TOF 4600 mass spectrometer was operated in data-dependent mode to switch automatically between MS and MS/MS acquisition. MS spectra were acquired across the mass range of 350–1250 m/z in high resolution mode using 250 ms accumulation time per spectrum. Tandem mass spectral scanned from 100-1250 m/z in high sensitivity mode with rolling collision energy. The 20 most intense precursors were selected for fragmentation per cycle with dynamic exclusion time of 9s.

Ventricular Myocyte Isolation

Myocytes were isolated using standard procedures. Briefly, the heart was connected to a plastic cannula for retrograde perfusion through the aorta and perfused with a Ca^{2+}-free solution containing (mmol/L) NaCl 117, KCl 5.7, $NaHCO_3$ 4.4, KH_2PO_4 1.5, $MgCl_2$ 17, HEPES 21.1, and glucose 11.7 (pH 7.4, equilibrated with O_2) for 5 min at 37°C. It was then perfused with the same solution containing 1 mg/mL collagenase II (283 U/mg; Worthington Biochemical, Lakewood, NJ, USA) until it became flaccid. Finally, it was perfused with Ca^{2+}-free solution for 5 min to remove enzymes. The LV was cut into small pieces and these fragments were shaken in the re-suspension medium at 37°C. Intact cells were enriched by centrifugation.

Measurement of Intracellular Calcium Transient and Cell Shortening

Isolated LV myocytes were placed in a bath on the stage of inverted microscopes for contractility and Ca^{2+} transients. The cells were washed at room temperature and continuously with a Tyrode solution containing (mmol/L): NaCl 140, KCl 5.4, $MgCl_2$ 1, HEPES 5, glucose 5.5 and $CaCl_2$ 1.0 (pH 7.4, adjusted with NaOH). Measurements were performed in field-stimulated cells at pace of 1 Hz by using IonOptix fluorescence and contractility systems (IonOptix, MA, USA) in room temperature. Contractions were elicited by rectangular depolarizing pulses, 2 ms in duration, and twice-diastolic threshold in intensity, by platinum electrodes. Cell

shortening was measured by edge-track detection. Ca^{2+} transients were measured by epifluorescence after loading the myocytes with 0.5 μM Fura-2 AM (Molecular Probes, OR, USA). The ratio of fluorescence emission intensities at 510 nm was sampled via a photo multiplier tube at 250 Hz under 340 and 380 nm excitation.

Statistical Analysis

All data are expressed as mean ± SEM and analyzed by SPSS 17. Two tailed Student's t-test was used to perform statistical analysis for comparisons between two groups. One way ANOVA with Bonnferoni multiple was applied for comparisons post-hoc test for more than two groups. A probability value less than 0.05 was considered as statistical significance.

References

1. Baines CP, J Zhang, G-W Wang, Y-T Zheng, JX Xiu, EM Cardwell, R Bolli, and P Ping. (2002). Mitochondrial PKCepsilon and MAPK form signaling modules in the murine heart: enhanced mitochondrial PKCepsilon-MAPK interactions and differential MAPK activation in PKCepsilon-induced cardioprotection. Circ Res 90:390-397.
2. Hu X, R Wu, Z Jiang, L Wang, P Chen, L Zhang, L Yang, Y Wu, H Chen, H Chen, Y Xu, Y Zhou, X Huang, KA Webster, H Yu, and Ja Wang. (2014). Leptin Signaling is Required for Augmented Therapeutic Properties of Mesenchymal Stem Cells Conferred by Hypoxia Preconditioning. Stem Cells
3. Chen P, R Wu, W Zhu, Z Jiang, Y Xu, H Chen, Z Zhang, L Zhang, H Yu, J Wang, and X Hu. (2014). Hypoxia preconditioned mesenchymal stem cells prevent cardiac fibroblast activation and collagen production via leptin. PLoS One 9:e103587.

Supplementary Figure

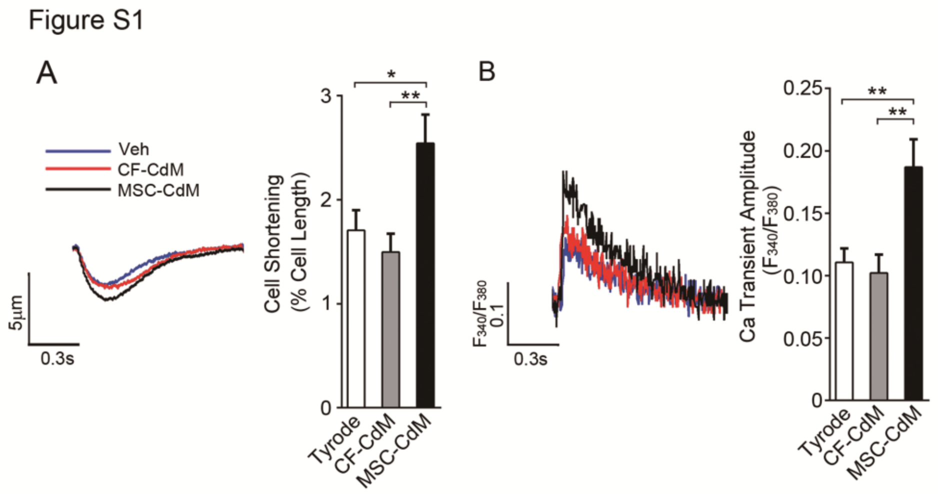

FIG. S1. Intracellular calcium transient and contractility of isolated adult myocyteswere recorded at 2-hreoxygenationfollowing 4-h hypoxia. **(A)** Typical recordings of contractility of myocytes are shown on the left panel. Contractility of myocytes was measured by video edge detection. Data repersent mean ±SEM of values from 15 cells. **(B)**Typical recordings of intracellular calcium transient (left panel) are shown. The amplitude of Intracellular calcium transient was assessed by IonOptix system. Data represent mean ± SEM of values from 15 cells.*P<0.05;**P<0.01.

Figure S2

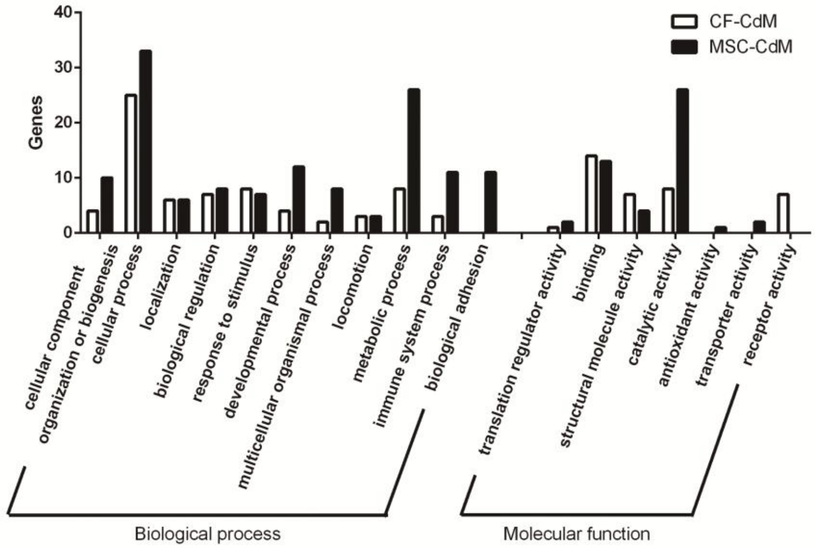

FIG. S2. Functional categorization of the proteins secreted from MSCs and CFs. Biological process and molecular function enrichment of secreted proteins.

Figure S3

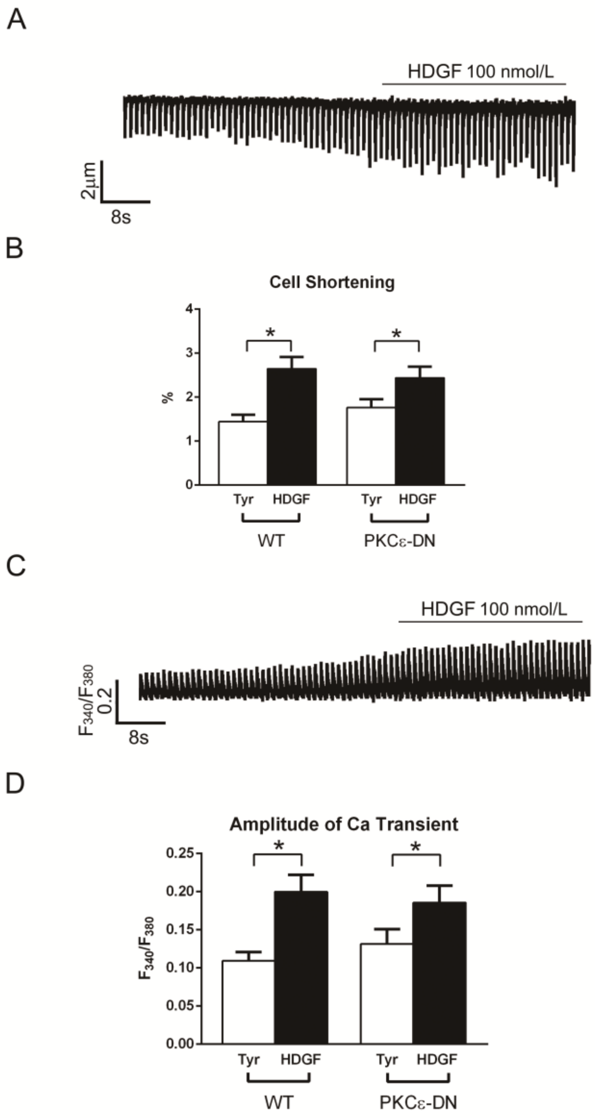

FIG. S3. HDGF enhanced calcium handling and mechanical properties despite of PKCε disruption. Adult myocytes isolated from PKCε-DN mice or WT littermate were subjected to 2 h of hypoxia followed by 4 h of reoxygenation with the treatment of vehicle or 100 nmol/L recombinant mouse HDGF. **(A)** Typical recording of myocyte contractility with the treatment of HDGF recorded by video edge detection. **(B)** Cell shortening fraction. **(C)** Typical recording of intracellular calcium transient with the treatment of HDGF recorded by the IonOptix system. **(D)** Amplitude of calcium transient. All Data represent mean± SEM of values from 15-21 cells. * P<0.05.

Table S1. MSC-CdM improved hemodynamic performance

	sham	I/R	CF-CdM	MSC-CdM
**LVP_{max} (mmHg)**	106±11.1	75.4±4.1	80.5±5.6	89.3±4.3*
**$+dP/dt_{max}$ (mmHg)**	7278±749	4010±580	4559±355	5935±627*
**$-dP/dt_{max}$ (mmHg)**	-6459±481	-3491±396	-4242±282	-5637±568*
**LVEDP (mmHg)**	3.1±0.5	6.6±0.9	5.6±0.9	4.4±0.7
**Exp Tau (ms)**	14.4±1.0	18.9±1.5	17.5±.1	16.8±1.1

Mice treated with CF-CdM, MSC-CdM or vehicle were subjected to hemodynamic measurement at 24-h reperfusion following 45 min of ischemia. Data are mean±SEM of values from six mice. LVP_{max}, maximum systolic LV pressure; $+dP/dt_{max}$, maximum positive first derivative of LVP;$-dP/dt_{max}$, maximum negative first derivative of LVP; LVEDP, Left Ventricular End-Diastolic Pressure; Exp Tau, Exponential Tau. *$P<0.05$ versus I/R.

Table S2. Recombinant HDGF improved hemodynamic performance via PKCε pathway

	WT sham	WT I/R	WT HDGF	DN sham	DN I/R	DN HDGF
**LVP_{max} (mmHg)**	109.5±4.1	75.4±3.6	86.5±3.2*	100.3±7.1	76.3±3.0	82.1±3.4
**$+dP/dt_{max}$ (mmHg)**	7255±527	4325±383	6079±412*	7413±665	4170±314	5017±209*#
**$-dP/dt_{max}$ (mmHg)**	-6617±856	-3690±332	-5062±279*	-6552±885	-3400±322	-4134±302#
**LVEDP (mmHg)**	3.3±0.7	6.0±0.9	4.4±1.3	3.7±0.8	6.4±0.8	5.7±1.0
**Exp Tau (ms)**	14.0±0.9	19.4±0.4	18.2±0.4	14.9±1.3	18.9±0.6	17.5±1.0

PKCε-DN and WT mice were subjected to hemodynamic measurement at 24-h reperfusion following 45 min of ischemia. Data are mean±SEM of values from six mice. LVP_{max}, maximum systolic LV pressure; $+dP/dt_{max}$, maximum positive first derivative of LVP;$-dP/dt_{max}$, maximum negative first derivative of LVP; LVEDP, Left Ventricular End-Diastolic Pressure; Exp Tau, Exponential Tau. *P<0.05 versus I/R; #P<0.05 versus WT HDGF.

Table S3: Proteins Identified by iTRAQ

Identified Proteins	CF-CdM1	CF-CdM2	CF-CdM3	MSC-CdM1	MSC-CdM2	MSC-CdM3
Cullin-4B OS=Mus musculus GN=Cul4b PE=1 SV=1	1.035	0.838	0.936	1.059	0.852	0.807
Receptor-type tyrosine-protein phosphatase F OS=Mus musculus GN=Ptprf PE=1 SV=1	1.000	1.244	1.122	1.370	0.631	1.501
Protein SZT2 OS=Mus musculus GN=Szt2 PE=1 SV=1	1.000	1.100	1.050	1.512	0.990	1.174
Obscurin OS=Mus musculus GN=Obscn PE=1 SV=2	1.000	0.996	0.998	0.699	1.230	0.726
Cytoskeleton-associated protein 5 OS=Mus musculus GN=Ckap5 PE=1 SV=1	1.000	0.770	0.885	0.602	0.790	0.623
Leucine zipper putative tumor suppressor 3 OS=Mus musculus GN=Lzts3 PE=1 SV=1	1.000	0.870	0.935	0.509	0.798	0.521
Protein virilizer homolog OS=Mus musculus GN=Kiaa1429 PE=1 SV=1	1.000	1.361	1.181	1.128	1.034	1.278
Tubulin beta-1 chain OS=Mus musculus GN=Tubb1 PE=1 SV=1	1.000	0.899	0.949	0.976	0.885	1.043
Agrin OS=Mus musculus GN=Agrn PE=1 SV=1	1.000	0.926	0.963	0.459	0.377	0.503
Titin OS=Mus musculus GN=Ttn PE=1 SV=1	1.000	0.975	0.987	1.171	0.893	1.495
Sushi, von Willebrand factor type A, EGF and pentraxin domain-containing protein 1 OS=Mus musculus GN=Svep1 PE=1 SV=1	1.000	0.934	0.967	0.347	0.377	0.441
AT-rich interactive domain-containing protein 1A OS=Mus musculus GN=Arid1a PE=1 SV=1	1.000	0.817	0.908	0.621	0.773	0.946
Inter alpha-trypsin inhibitor, heavy chain 4 OS=Mus musculus GN=Itih4 PE=1 SV=2	1.000	1.041	1.021	1.204	1.062	1.644
Metalloprotease TIKI2 OS=Mus musculus GN=Trabd2b PE=2 SV=1	1.000	1.042	1.021	0.962	0.986	0.981
N-acetyllactosaminide beta-1,3-N-acetylglucosaminyltransferase OS=Mus musculus GN=B3gnt1 PE=2 SV=1	1.000	1.051	1.026	0.911	0.967	1.216
Scaffold attachment factor B1 OS=Mus musculus GN=Safb PE=1 SV=2	1.126	0.957	1.041	1.399	1.465	1.306
Bifunctional ATP-dependent dihydroxyacetone kinase/FAD-AMP lyase (cyclizing) OS=Mus musculus GN=Dak PE=2 SV=1	1.000	0.823	0.911	1.221	1.323	1.348
Coiled-coil domain-containing protein 62 OS=Mus musculus GN=Ccdc62 PE=3 SV=1	1.000	1.120	1.060	1.009	0.559	1.000
Ryanodine receptor 1 OS=Mus musculus GN=Ryr1 PE=1 SV=1	1.000	1.070	1.035	0.655	0.382	0.704
Interferon regulatory factor 2-binding protein 2 OS=Mus musculus GN=Irf2bp2 PE=1 SV=1	1.000	1.541	1.270	1.825	1.308	1.086
Apolipoprotein B-100 OS=Mus musculus GN=Apob PE=1 SV=1	0.936	1.106	1.021	0.917	0.997	1.002
Nucleoprotein TPR OS=Mus musculus GN=Tpr PE=1 SV=1	1.000	0.947	0.973	1.128	0.898	1.140
FERM, RhoGEF and pleckstrin domain-containing protein 1 OS=Mus musculus GN=Farp1 PE=1 SV=1	1.000	0.921	0.961	0.920	0.715	0.856
Mitochondrial inner membrane protein OS=Mus musculus GN=Immt PE=1 SV=1	1.000	0.952	0.976	0.945	0.577	0.863
Serine/threonine-protein kinase MST4 OS=Mus musculus GN=Mst4 PE=2 SV=1	0.945	1.137	1.041	1.510	1.666	0.869
Alpha-tectorin OS=Mus musculus GN=Tecta PE=1 SV=2	1.000	0.938	0.969	1.102	0.618	1.025
Calpain-2 catalytic subunit OS=Mus musculus GN=Capn2 PE=1 SV=4	1.000	0.917	0.958	0.732	0.727	0.716
Myc box-dependent-interacting protein 1 OS=Mus musculus GN=Bin1 PE=1 SV=1	1.070	1.168	1.119	1.455	1.630	1.647
Vesicle-trafficking protein SEC22b OS=Mus musculus GN=Sec22b PE=1 SV=3	1.000	1.057	1.029	0.819	0.857	0.992

**Dihydropyrimidinase-related protein 2 OS=Mus musculus GN=Dpysl2 PE=1 SV=2**	1.042	1.028	1.035	1.108	1.131	1.166
**THO complex subunit 4 OS=Mus musculus GN=Alyref PE=1 SV=3**	0.992	1.065	1.029	1.419	1.399	1.725
**Clathrin light chain A OS=Mus musculus GN=Clta PE=1 SV=2**	1.000	1.032	1.016	0.811	0.903	1.083
**Methionine aminopeptidase 2 OS=Mus musculus GN=Metap2 PE=1 SV=1**	1.054	0.984	1.019	1.517	1.478	1.617
**Peroxiredoxin-6 OS=Mus musculus GN=Prdx6 PE=1 SV=3**	1.055	1.025	1.040	1.386	1.394	1.276
**Dihydrolipoyl dehydrogenase, mitochondrial OS=Mus musculus GN=Dld PE=1 SV=2**	1.000	0.919	0.960	0.771	0.881	0.696
**Ubiquitin-protein ligase E3A OS=Mus musculus GN=Ube3a PE=1 SV=2**	1.000	0.990	0.995	1.597	0.868	1.943
**Treacle protein OS=Mus musculus GN=Tcof1 PE=1 SV=1**	0.983	1.575	1.279	3.300	4.498	5.857
**Dynactin subunit 1 OS=Mus musculus GN=Dctn1 PE=1 SV=3**	1.087	0.979	1.033	0.726	0.752	0.750
**Glucosidase 2 subunit beta OS=Mus musculus GN=Prkcsh PE=1 SV=1**	1.036	1.057	1.047	0.867	1.180	0.837
**Peroxiredoxin-4 OS=Mus musculus GN=Prdx4 PE=1 SV=1**	0.998	1.115	1.057	0.776	0.867	0.965
**Protein diaphanous homolog 1 OS=Mus musculus GN=Diaph1 PE=1 SV=1**	1.000	0.792	0.896	0.340	0.480	0.711
**116 kDa U5 small nuclear ribonucleoprotein component OS=Mus musculus GN=Eftud2 PE=1 SV=1**	1.000	1.064	1.032	1.080	0.926	0.892
**AH receptor-interacting protein OS=Mus musculus GN=Aip PE=1 SV=1**	0.997	0.928	0.963	1.292	1.094	1.095
**Syntenin-1 OS=Mus musculus GN=Sdcbp PE=1 SV=1**	1.261	1.316	1.288	1.691	1.672	2.069
**Copper transport protein ATOX1 OS=Mus musculus GN=Atox1 PE=1 SV=1**	0.934	0.902	0.918	1.463	1.509	1.474
**Latent-transforming growth factor beta-binding protein 2 OS=Mus musculus GN=Ltbp2 PE=1 SV=2**	0.993	0.928	0.961	1.048	0.982	1.077
**Proteasome subunit beta type-1 OS=Mus musculus GN=Psmb1 PE=1 SV=1**	1.174	1.320	1.247	2.135	2.076	1.896
**Histone deacetylase 1 OS=Mus musculus GN=Hdac1 PE=1 SV=1**	1.000	1.035	1.017	0.782	0.826	1.250
**Glutathione S-transferase omega-1 OS=Mus musculus GN=Gsto1 PE=1 SV=2**	1.084	1.050	1.067	2.248	2.506	2.221
**Lysosomal alpha-mannosidase OS=Mus musculus GN=Man2b1 PE=1 SV=4**	1.089	1.128	1.109	0.908	0.888	0.991
**Calsequestrin-2 OS=Mus musculus GN=Casq2 PE=1 SV=3**	1.000	1.301	1.151	0.321	0.581	0.350
**Extracellular superoxide dismutase [Cu-Zn] OS=Mus musculus GN=Sod3 PE=1 SV=1**	1.068	1.181	1.125	2.540	2.909	2.113
**Glutamate--cysteine ligase regulatory subunit OS=Mus musculus GN=Gclm PE=1 SV=1**	0.957	0.941	0.949	1.042	1.243	1.345
**Dihydropyrimidinase-related protein 4 OS=Mus musculus GN=Dpysl4 PE=1 SV=1**	1.000	1.285	1.142	1.604	1.467	2.139
**Prohibitin-2 OS=Mus musculus GN=Phb2 PE=1 SV=1**	1.000	1.161	1.080	0.460	0.436	1.356
**D-dopachrome decarboxylase OS=Mus musculus GN=Ddt PE=1 SV=3**	0.996	1.131	1.064	1.054	0.869	1.429
**26S proteasome non-ATPase regulatory subunit 4 OS=Mus musculus GN=Psmd4 PE=1 SV=1**	1.000	1.106	1.053	1.266	1.899	1.300
**Pre-mRNA-splicing factor ATP-dependent RNA helicase DHX15 OS=Mus musculus GN=Dhx15 PE=1 SV=2**	1.000	0.970	0.985	1.150	0.952	1.036
**Serine/arginine-rich splicing factor 5 OS=Mus**	1.000	1.005	1.002	0.510	0.757	1.091

**musculus GN=Srsf5 PE=1 SV=2**						
**Importin subunit alpha-3 OS=Mus musculus GN=Kpna4 PE=1 SV=1**	1.018	1.104	1.061	1.392	1.390	1.406
**Importin subunit alpha-4 OS=Mus musculus GN=Kpna3 PE=1 SV=1**	1.000	1.036	1.018	0.840	1.051	1.111
**Calpain-1 catalytic subunit OS=Mus musculus GN=Capn1 PE=1 SV=1**	1.000	1.286	1.143	0.843	0.813	1.269
**Acidic leucine-rich nuclear phosphoprotein 32 family member A OS=Mus musculus GN=Anp32a PE=1 SV=1**	1.061	1.073	1.067	1.564	1.724	1.654
**26S proteasome non-ATPase regulatory subunit 14 OS=Mus musculus GN=Psmd14 PE=1 SV=2**	1.000	1.026	1.013	0.995	0.797	1.080
**Cohesin subunit SA-2 OS=Mus musculus GN=Stag2 PE=1 SV=3**	1.000	1.015	1.008	1.177	0.705	1.169
**Annexin A3 OS=Mus musculus GN=Anxa3 PE=1 SV=4**	1.002	0.938	0.970	0.822	0.867	0.839
**AP-1 complex subunit beta-1 OS=Mus musculus GN=Ap1b1 PE=1 SV=2**	1.000	0.866	0.933	1.027	0.995	1.047
**Interferon alpha/beta receptor 2 OS=Mus musculus GN=Ifnar2 PE=1 SV=2**	0.975	1.124	1.049	0.918	0.710	0.968
**Myeloid-associated differentiation marker OS=Mus musculus GN=Myadm PE=1 SV=2**	1.000	0.812	0.906	0.507	0.493	1.011
**Nuclear migration protein nudC OS=Mus musculus GN=Nudc PE=1 SV=1**	1.001	1.069	1.035	1.482	1.562	1.562
**Heterogeneous nuclear ribonucleoprotein H OS=Mus musculus GN=Hnrnph1 PE=1 SV=3**	1.000	0.973	0.987	1.340	1.552	1.255
**Apoptosis inhibitor 5 OS=Mus musculus GN=Api5 PE=1 SV=2**	1.127	1.160	1.143	1.773	1.589	1.855
**Calumenin OS=Mus musculus GN=Calu PE=1 SV=1**	1.024	1.014	1.019	0.604	0.729	0.558
**Frataxin, mitochondrial OS=Mus musculus GN=Fxn PE=1 SV=1**	1.000	1.099	1.050	1.608	1.555	2.333
**Syndecan-4 OS=Mus musculus GN=Sdc4 PE=1 SV=1**	1.067	1.269	1.168	0.591	0.728	0.663
**Polymerase I and transcript release factor OS=Mus musculus GN=Ptrf PE=1 SV=1**	1.034	1.038	1.036	0.765	0.886	0.777
**AP-3 complex subunit delta-1 OS=Mus musculus GN=Ap3d1 PE=1 SV=1**	1.000	1.264	1.132	1.644	0.746	0.929
**WNT1-inducible-signaling pathway protein 1 OS=Mus musculus GN=Wisp1 PE=2 SV=1**	1.000	1.048	1.024	0.667	0.762	1.052
**Bystin OS=Mus musculus GN=Bysl PE=1 SV=3**	0.967	1.011	0.989	1.204	0.336	1.455
**High mobility group protein B3 OS=Mus musculus GN=Hmgb3 PE=1 SV=3**	1.000	1.165	1.082	2.483	2.728	2.626
**A-kinase anchor protein 2 OS=Mus musculus GN=Akap2 PE=1 SV=3**	1.000	1.093	1.047	0.836	0.792	0.865
**Barrier-to-autointegration factor OS=Mus musculus GN=Banf1 PE=1 SV=1**	1.042	0.944	0.993	0.980	1.221	1.239
**STE20-like serine/threonine-protein kinase OS=Mus musculus GN=Slk PE=1 SV=2**	1.030	1.054	1.042	1.271	1.631	1.260
**Inositol monophosphatase 1 OS=Mus musculus GN=Impa1 PE=1 SV=1**	1.066	0.960	1.013	1.112	1.172	1.121
**Coatomer subunit beta' OS=Mus musculus GN=Copb2 PE=1 SV=2**	1.023	0.969	0.996	1.029	1.030	1.014
**Septin-7 OS=Mus musculus GN=Sept7 PE=1 SV=1**	1.036	1.158	1.097	0.995	1.388	1.174
**Eukaryotic translation initiation factor 6 OS=Mus musculus GN=Eif6 PE=1 SV=2**	0.973	1.019	0.996	1.129	1.251	1.125
**Stanniocalcin-1 OS=Mus musculus GN=Stc1 PE=2 SV=1**	1.112	1.071	1.091	1.034	0.805	1.185
**Transcription elongation factor SPT5 OS=Mus musculus GN=Supt5h PE=1 SV=1**	1.000	0.859	0.930	1.106	0.778	1.205
**Proteasome subunit beta type-5 OS=Mus musculus GN=Psmb5 PE=1 SV=3**	0.971	0.962	0.966	1.090	1.116	1.323

**ATP-dependent RNA helicase A OS=Mus musculus GN=Dhx9 PE=1 SV=2**	1.000	1.196	1.098	1.401	1.231	1.148
**Eukaryotic translation initiation factor 3 subunit D OS=Mus musculus GN=Eif3d PE=1 SV=2**	1.000	0.891	0.945	0.758	0.816	0.972
**Elongation factor 1-beta OS=Mus musculus GN=Eef1b PE=1 SV=5**	0.981	0.965	0.973	1.366	1.681	1.334
**Glycylpeptide N-tetradecanoyltransferase 1 OS=Mus musculus GN=Nmt1 PE=1 SV=1**	1.000	1.375	1.188	1.142	1.174	0.817
**Gremlin-1 OS=Mus musculus GN=Grem1 PE=2 SV=1**	1.000	0.983	0.991	1.267	1.376	0.928
**PDZ and LIM domain protein 1 OS=Mus musculus GN=Pdlim1 PE=1 SV=4**	1.000	1.034	1.017	1.410	1.216	1.305
**Proteasome subunit alpha type-3 OS=Mus musculus GN=Psma3 PE=1 SV=3**	0.992	0.994	0.993	1.494	1.891	1.501
**Syntaxin-7 OS=Mus musculus GN=Stx7 PE=1 SV=3**	1.000	1.120	1.060	1.586	1.372	1.337
**Oncostatin-M-specific receptor subunit beta OS=Mus musculus GN=Osmr PE=1 SV=1**	1.000	0.976	0.988	0.885	0.621	1.113
**UDP-glucose 6-dehydrogenase OS=Mus musculus GN=Ugdh PE=1 SV=1**	1.089	1.112	1.100	1.280	1.307	1.317
**Sorting nexin-3 OS=Mus musculus GN=Snx3 PE=1 SV=3**	1.000	0.921	0.960	1.284	1.036	1.143
**Sorting nexin-12 OS=Mus musculus GN=Snx12 PE=1 SV=1**	1.000	0.912	0.956	0.839	0.713	0.705
**Prefoldin subunit 2 OS=Mus musculus GN=Pfdn2 PE=1 SV=2**	1.079	1.000	1.040	1.137	1.076	1.201
**E3 ubiquitin-protein ligase TTC3 OS=Mus musculus GN=Ttc3 PE=1 SV=2**	1.042	1.004	1.023	0.975	0.787	1.206
**Collagen alpha-1(V) chain OS=Mus musculus GN=Col5a1 PE=1 SV=2**	1.019	0.991	1.005	0.407	0.351	0.422
**Nidogen-2 OS=Mus musculus GN=Nid2 PE=1 SV=2**	1.089	1.068	1.078	0.631	0.603	0.693
**WD repeat-containing protein 1 OS=Mus musculus GN=Wdr1 PE=1 SV=3**	1.018	1.018	1.018	1.066	1.107	0.976
**Transforming growth factor beta receptor type 3 OS=Mus musculus GN=Tgfbr3 PE=1 SV=1**	0.963	0.986	0.975	1.252	1.197	1.509
**Calpain small subunit 1 OS=Mus musculus GN=Capns1 PE=1 SV=1**	1.141	1.277	1.209	1.133	1.121	1.519
**Cytoplasmic dynein 1 intermediate chain 2 OS=Mus musculus GN=Dync1i2 PE=1 SV=1**	0.997	1.209	1.103	2.183	1.690	1.891
**AP-1 complex subunit gamma-like 2 OS=Mus musculus GN=Ap1g2 PE=1 SV=2**	1.000	1.114	1.057	0.447	0.392	0.488
**Palmitoyl-protein thioesterase 1 OS=Mus musculus GN=Ppt1 PE=1 SV=2**	1.000	1.125	1.062	0.965	0.991	0.951
**COP9 signalosome complex subunit 3 OS=Mus musculus GN=Cops3 PE=1 SV=3**	1.000	1.046	1.023	1.127	1.231	1.325
**COP9 signalosome complex subunit 4 OS=Mus musculus GN=Cops4 PE=1 SV=1**	0.966	1.093	1.030	1.067	1.292	1.171
**Heterogeneous nuclear ribonucleoproteins A2/B1 OS=Mus musculus GN=Hnrnpa2b1 PE=1 SV=2**	1.090	1.081	1.085	1.825	1.922	1.673
**Serine/threonine-protein kinase PAK 1 OS=Mus musculus GN=Pak1 PE=1 SV=1**	1.000	0.787	0.894	0.726	0.503	1.038
**26S protease regulatory subunit 6A OS=Mus musculus GN=Psmc3 PE=1 SV=2**	0.976	1.002	0.989	1.216	1.114	1.211
**ATP-dependent Clp protease proteolytic subunit, mitochondrial OS=Mus musculus GN=Clpp PE=1 SV=1**	1.000	1.062	1.031	1.048	1.159	1.199
**Coagulation factor V OS=Mus musculus GN=F5 PE=1 SV=1**	1.000	1.114	1.057	1.064	0.928	1.164
**Disintegrin and metalloproteinase domain-containing protein 15 OS=Mus musculus GN=Adam15 PE=1 SV=2**	1.000	0.859	0.930	0.916	0.575	0.793
**Isocitrate dehydrogenase [NADP] cytoplasmic**	1.008	0.913	0.960	1.330	1.252	1.241

**OS=Mus musculus GN=Idh1 PE=1 SV=2**						
**Coagulation factor X OS=Mus musculus GN=F10 PE=1 SV=1**	1.000	1.024	1.012	0.855	0.748	0.973
**Protein lin-7 homolog C OS=Mus musculus GN=Lin7c PE=1 SV=2**	1.000	0.962	0.981	0.946	1.654	1.508
**Glucosamine-6-phosphate isomerase 1 OS=Mus musculus GN=Gnpda1 PE=1 SV=3**	1.000	0.980	0.990	1.065	1.249	1.544
**Transcobalamin-2 OS=Mus musculus GN=Tcn2 PE=1 SV=1**	1.000	0.916	0.958	0.595	0.491	0.541
**Alpha-actinin-3 OS=Mus musculus GN=Actn3 PE=2 SV=1**	1.000	0.992	0.996	0.773	0.867	1.185
**Legumain OS=Mus musculus GN=Lgmn PE=1 SV=1**	0.865	0.942	0.903	1.108	1.258	1.205
**Afamin OS=Mus musculus GN=Afm PE=1 SV=2**	1.000	0.872	0.936	1.001	0.615	1.322
**Tripeptidyl-peptidase 1 OS=Mus musculus GN=Tpp1 PE=1 SV=2**	1.071	1.097	1.084	0.704	0.702	0.709
**Integral membrane protein 2B OS=Mus musculus GN=Itm2b PE=1 SV=1**	1.000	0.813	0.906	0.874	0.820	0.660
**Coatomer subunit epsilon OS=Mus musculus GN=Cope PE=1 SV=3**	0.975	0.933	0.954	1.357	0.967	1.140
**RNA-binding protein 3 OS=Mus musculus GN=Rbm3 PE=1 SV=1**	1.026	0.887	0.956	1.258	1.284	1.160
**Cytochrome b OS=Mus musculus GN=Mt-Cyb PE=1 SV=1**	1.000	1.373	1.186	1.935	0.912	0.703
**Dihydrofolate reductase OS=Mus musculus GN=Dhfr PE=1 SV=3**	1.000	1.348	1.174	3.270	4.792	2.876
**Hypoxanthine-guanine phosphoribosyltransferase OS=Mus musculus GN=Hprt1 PE=1 SV=3**	1.028	0.957	0.993	2.147	2.088	1.812
**Complement C3 OS=Mus musculus GN=C3 PE=1 SV=3**	0.998	0.994	0.996	1.154	1.097	1.184
**Complement C4-B OS=Mus musculus GN=C4b PE=1 SV=3**	1.004	0.980	0.992	0.860	0.663	0.967
**Beta-2-microglobulin OS=Mus musculus GN=B2m PE=1 SV=2**	1.031	1.011	1.021	0.939	0.672	0.885
**H-2 class I histocompatibility antigen, alpha chain (Fragment) OS=Mus musculus GN=H2-D1 PE=1 SV=1**	1.000	1.109	1.055	3.434	1.776	2.107
**H-2 class I histocompatibility antigen, D-B alpha chain OS=Mus musculus GN=H2-D1 PE=1 SV=2**	0.975	0.961	0.968	0.452	0.329	0.487
**H-2 class I histocompatibility antigen, K-B alpha chain OS=Mus musculus GN=H2-K1 PE=1 SV=1**	1.040	1.161	1.101	0.817	0.714	0.958
**H-2 class I histocompatibility antigen, K-D alpha chain OS=Mus musculus GN=H2-K1 PE=1 SV=1**	1.008	0.924	0.966	1.185	1.183	1.132
**Hemoglobin subunit alpha OS=Mus musculus GN=Hba PE=1 SV=2**	1.026	1.164	1.095	1.253	1.272	1.293
**Hemoglobin subunit beta-1 OS=Mus musculus GN=Hbb-b1 PE=1 SV=2**	0.704	0.787	0.745	0.606	0.508	0.605
**Collagen alpha-1(IV) chain OS=Mus musculus GN=Col4a1 PE=1 SV=4**	0.937	0.994	0.965	0.358	0.292	0.473
**Laminin subunit gamma-1 OS=Mus musculus GN=Lamc1 PE=1 SV=2**	1.009	1.037	1.023	0.417	0.325	0.422
**Laminin subunit beta-1 OS=Mus musculus GN=Lamb1 PE=1 SV=3**	0.998	1.053	1.026	0.480	0.399	0.518
**Keratin, type I cytoskeletal 10 OS=Mus musculus GN=Krt10 PE=1 SV=3**	0.925	0.733	0.829	0.459	0.446	0.469
**Adenosine deaminase OS=Mus musculus GN=Ada PE=1 SV=3**	1.000	1.483	1.241	2.980	2.023	2.923
**Keratin, type II cytoskeletal 1 OS=Mus musculus GN=Krt1 PE=1 SV=4**	0.975	0.725	0.850	0.370	0.301	0.423
**Fatty acid-binding protein, adipocyte OS=Mus musculus GN=Fabp4 PE=1 SV=3**	1.000	1.028	1.014	1.004	0.902	1.006
**Complement factor B OS=Mus musculus GN=Cfb**	1.064	1.099	1.082	0.816	0.628	0.870

**PE=1 SV=2**						
**Myoglobin OS=Mus musculus GN=Mb PE=1 SV=3**	1.000	1.569	1.284	0.669	0.370	0.899
**Prolactin-2C3 OS=Mus musculus GN=Prl2c3 PE=1 SV=2**	0.954	0.961	0.958	1.468	1.947	1.596
**Serum amyloid A-3 protein OS=Mus musculus GN=Saa3 PE=1 SV=1**	1.255	1.264	1.260	3.199	4.324	2.834
**Major prion protein OS=Mus musculus GN=Prnp PE=1 SV=2**	1.000	1.099	1.049	1.056	0.601	0.847
**Fructose-bisphosphate aldolase A OS=Mus musculus GN=Aldoa PE=1 SV=2**	1.008	0.986	0.997	1.569	1.484	1.423
**Natriuretic peptides A OS=Mus musculus GN=Nppa PE=1 SV=2**	1.000	0.935	0.967	0.170	0.082	0.162
**cAMP-dependent protein kinase catalytic subunit alpha OS=Mus musculus GN=Prkaca PE=1 SV=3**	1.000	1.000	1.000	1.392	1.421	1.360
**Aspartate aminotransferase, cytoplasmic OS=Mus musculus GN=Got1 PE=1 SV=3**	0.934	1.103	1.019	1.218	1.142	1.195
**Aspartate aminotransferase, mitochondrial OS=Mus musculus GN=Got2 PE=1 SV=1**	1.044	1.071	1.057	0.740	1.193	0.692
**Tubulin alpha-1B chain OS=Mus musculus GN=Tuba1b PE=1 SV=2**	0.913	0.949	0.931	1.194	1.421	1.320
**L-lactate dehydrogenase A chain OS=Mus musculus GN=Ldha PE=1 SV=3**	1.089	1.162	1.126	2.834	3.610	2.566
**Glucose-6-phosphate isomerase OS=Mus musculus GN=Gpi PE=1 SV=4**	1.149	1.182	1.166	1.719	1.888	1.459
**Cathepsin L1 OS=Mus musculus GN=Ctsl PE=1 SV=2**	1.017	0.953	0.985	0.661	0.595	0.616
**NADP-dependent malic enzyme OS=Mus musculus GN=Me1 PE=1 SV=2**	1.012	1.090	1.051	1.346	1.566	1.420
**Urokinase-type plasminogen activator OS=Mus musculus GN=Plau PE=1 SV=1**	1.000	0.844	0.922	0.923	0.837	0.875
**Complement factor H OS=Mus musculus GN=Cfh PE=1 SV=2**	1.065	1.142	1.104	0.736	0.703	0.779
**Protein S100-A4 OS=Mus musculus GN=S100a4 PE=1 SV=1**	1.000	1.052	1.026	1.568	2.223	1.371
**Macrophage colony-stimulating factor 1 OS=Mus musculus GN=Csf1 PE=1 SV=2**	1.141	1.147	1.144	0.276	0.313	0.263
**SPARC OS=Mus musculus GN=Sparc PE=1 SV=1**	1.051	0.978	1.014	0.370	0.326	0.375
**Annexin A2 OS=Mus musculus GN=Anxa2 PE=1 SV=2**	1.090	1.122	1.106	0.797	1.078	0.945
**Thymidylate synthase OS=Mus musculus GN=Tyms PE=1 SV=1**	1.000	0.942	0.971	1.255	1.256	1.047
**Serum albumin OS=Mus musculus GN=Alb PE=1 SV=3**	0.894	0.827	0.860	0.869	0.511	0.926
**Heat shock protein HSP 90-alpha OS=Mus musculus GN=Hsp90aa1 PE=1 SV=4**	1.001	1.028	1.014	1.962	2.588	2.188
**Phosphorylase b kinase gamma catalytic chain, skeletal muscle/heart isoform OS=Mus musculus GN=Phkg1 PE=1 SV=3**	1.000	1.053	1.026	1.038	1.013	1.240
**Protein disulfide-isomerase A4 OS=Mus musculus GN=Pdia4 PE=1 SV=3**	0.996	0.917	0.957	1.051	0.872	1.111
**Endoplasmin OS=Mus musculus GN=Hsp90b1 PE=1 SV=2**	1.070	1.043	1.056	1.007	0.840	0.971
**Collagen alpha-1(III) chain OS=Mus musculus GN=Col3a1 PE=1 SV=4**	1.000	1.022	1.011	0.555	0.469	0.524
**Collagen alpha-2(IV) chain OS=Mus musculus GN=Col4a2 PE=1 SV=4**	1.017	1.011	1.014	0.311	0.248	0.338
**Protein S100-A10 OS=Mus musculus GN=S100a10 PE=1 SV=2**	1.000	1.095	1.047	0.901	1.151	1.037
**Apolipoprotein E OS=Mus musculus GN=Apoe PE=1 SV=2**	1.000	1.059	1.030	0.713	0.584	0.692
**Superoxide dismutase [Cu-Zn] OS=Mus musculus**	0.952	0.984	0.968	1.510	1.530	1.285

GN=Sod1 PE=1 SV=2						
Malate dehydrogenase, mitochondrial OS=Mus musculus GN=Mdh2 PE=1 SV=3	1.016	0.985	1.001	0.713	0.889	0.635
Interleukin-6 OS=Mus musculus GN=Il6 PE=1 SV=1	1.000	0.948	0.974	0.935	0.936	0.844
Guanine nucleotide-binding protein G(i) subunit alpha-2 OS=Mus musculus GN=Gnai2 PE=1 SV=5	1.000	1.460	1.230	1.025	1.264	1.241
Lysozyme C-2 OS=Mus musculus GN=Lyz2 PE=1 SV=2	1.000	0.878	0.939	0.222	0.156	0.197
Integrin beta-1 OS=Mus musculus GN=Itgb1 PE=1 SV=1	1.000	0.920	0.960	0.814	0.872	0.694
Leukemia inhibitory factor OS=Mus musculus GN=Lif PE=1 SV=1	1.000	0.878	0.939	0.678	0.495	1.029
Protein disulfide-isomerase OS=Mus musculus GN=P4hb PE=1 SV=2	1.083	1.111	1.097	1.000	1.185	0.894
Nucleolin OS=Mus musculus GN=Ncl PE=1 SV=2	1.084	1.003	1.043	1.655	1.844	1.548
Phosphoglycerate kinase 1 OS=Mus musculus GN=Pgk1 PE=1 SV=4	0.986	1.012	0.999	1.934	2.334	1.927
Ferritin heavy chain OS=Mus musculus GN=Fth1 PE=1 SV=2	1.030	1.131	1.081	1.500	2.035	1.858
Insulin-like growth factor II OS=Mus musculus GN=Igf2 PE=1 SV=1	1.000	0.855	0.928	0.460	0.268	0.467
Superoxide dismutase [Mn], mitochondrial OS=Mus musculus GN=Sod2 PE=1 SV=3	0.994	0.985	0.989	0.760	1.016	0.672
Diphosphoinositol polyphosphate phosphohydrolase 3-alpha OS=Mus musculus GN=Nudt10 PE=1 SV=1	1.000	1.433	1.216	1.389	1.601	2.350
Uncharacterized protein C14orf142 homolog OS=Mus musculus PE=3 SV=1	1.000	0.839	0.919	0.962	0.541	0.692
Annexin A1 OS=Mus musculus GN=Anxa1 PE=1 SV=2	1.126	1.238	1.182	0.586	0.748	0.653
Elongation factor 1-alpha 1 OS=Mus musculus GN=Eef1a1 PE=1 SV=3	1.023	1.030	1.027	2.305	2.697	2.590
C-C motif chemokine 2 OS=Mus musculus GN=Ccl2 PE=1 SV=1	0.993	1.040	1.017	0.389	0.382	0.394
MLV-related proviral Env polyprotein OS=Mus musculus PE=1 SV=3	1.000	0.888	0.944	0.941	1.130	0.932
Nidogen-1 OS=Mus musculus GN=Nid1 PE=1 SV=2	1.132	1.060	1.096	0.522	0.497	0.533
Delta-aminolevulinic acid dehydratase OS=Mus musculus GN=Alad PE=1 SV=1	1.000	1.512	1.256	0.711	0.577	0.896
Cathepsin B OS=Mus musculus GN=Ctsb PE=1 SV=2	1.023	0.998	1.010	1.182	1.132	1.279
Eukaryotic initiation factor 4A-II OS=Mus musculus GN=Eif4a2 PE=1 SV=2	1.000	1.142	1.071	1.795	1.640	1.647
Thioredoxin OS=Mus musculus GN=Txn PE=1 SV=3	1.016	0.983	0.999	1.675	1.823	1.605
Glutathione S-transferase Mu 1 OS=Mus musculus GN=Gstm1 PE=1 SV=2	1.000	1.027	1.013	1.083	0.751	0.914
Transcription elongation factor A protein 1 OS=Mus musculus GN=Tcea1 PE=1 SV=2	0.992	1.025	1.009	1.662	1.633	1.498
Monocyte differentiation antigen CD14 OS=Mus musculus GN=Cd14 PE=1 SV=1	1.000	1.008	1.004	0.504	0.712	0.955
4F2 cell-surface antigen heavy chain OS=Mus musculus GN=Slc3a2 PE=1 SV=1	1.000	1.008	1.004	0.595	0.950	0.699
C-X-C motif chemokine 2 OS=Mus musculus GN=Cxcl2 PE=1 SV=2	1.000	1.015	1.008	0.212	0.145	0.186
Osteopontin OS=Mus musculus GN=Spp1 PE=1 SV=1	1.068	0.995	1.031	4.581	4.873	4.190
Activated RNA polymerase II transcriptional coactivator p15 OS=Mus musculus GN=Sub1 PE=1 SV=3	1.098	0.992	1.045	1.017	1.134	1.079
Collagen alpha-1(I) chain OS=Mus musculus GN=Col1a1 PE=1 SV=4	0.920	0.910	0.915	0.558	0.491	0.506

**Poly [ADP-ribose] polymerase 1 OS=Mus musculus GN=Parp1 PE=1 SV=3**	0.932	0.872	0.902	0.851	0.838	0.794
**Lipoprotein lipase OS=Mus musculus GN=Lpl PE=1 SV=3**	0.992	1.090	1.041	0.951	0.782	0.981
**Tissue-type plasminogen activator OS=Mus musculus GN=Plat PE=1 SV=3**	1.000	0.860	0.930	0.495	0.528	0.647
**Fibronectin OS=Mus musculus GN=Fn1 PE=1 SV=4**	0.964	0.958	0.961	0.356	0.356	0.332
**Fatty acid-binding protein, heart OS=Mus musculus GN=Fabp3 PE=1 SV=5**	1.000	1.209	1.104	1.210	0.580	0.833
**Lysosome-associated membrane glycoprotein 1 OS=Mus musculus GN=Lamp1 PE=1 SV=2**	1.224	1.335	1.280	1.003	1.451	1.214
**Cyclin-dependent kinase 1 OS=Mus musculus GN=Cdk1 PE=1 SV=3**	1.000	0.924	0.962	1.364	1.187	1.142
**Heat shock protein HSP 90-beta OS=Mus musculus GN=Hsp90ab1 PE=1 SV=3**	0.971	1.002	0.987	2.297	2.457	1.916
**Neutrophil gelatinase-associated lipocalin OS=Mus musculus GN=Lcn2 PE=1 SV=1**	0.958	0.969	0.963	2.798	1.897	2.618
**T-complex protein 1 subunit alpha OS=Mus musculus GN=Tcp1 PE=1 SV=3**	1.079	1.089	1.084	1.324	1.200	1.219
**Amyloid beta A4 protein OS=Mus musculus GN=App PE=1 SV=3**	1.037	1.033	1.035	0.523	0.459	0.511
**Metalloproteinase inhibitor 1 OS=Mus musculus GN=Timp1 PE=1 SV=2**	1.062	1.080	1.071	1.315	1.336	1.097
**ATP-dependent 6-phosphofructokinase, liver type OS=Mus musculus GN=Pfkl PE=1 SV=4**	1.000	0.857	0.929	0.755	0.675	0.844
**Plasminogen activator inhibitor 2, macrophage OS=Mus musculus GN=Serpinb2 PE=1 SV=1**	0.959	0.882	0.921	1.082	1.480	1.155
**cAMP-dependent protein kinase type I-beta regulatory subunit OS=Mus musculus GN=Prkar1b PE=1 SV=2**	1.000	0.893	0.947	1.179	1.101	1.634
**Growth-regulated alpha protein OS=Mus musculus GN=Cxcl1 PE=1 SV=1**	1.105	1.292	1.199	0.893	0.922	0.807
**Gelsolin OS=Mus musculus GN=Gsn PE=1 SV=3**	0.973	0.972	0.973	0.936	0.923	0.999
**Neural cell adhesion molecule 1 OS=Mus musculus GN=Ncam1 PE=1 SV=3**	1.037	0.923	0.980	0.943	1.253	1.217
**Intercellular adhesion molecule 1 OS=Mus musculus GN=Icam1 PE=1 SV=1**	1.000	1.005	1.003	0.302	0.313	0.208
**Protein S100-A6 OS=Mus musculus GN=S100a6 PE=1 SV=3**	1.005	0.991	0.998	1.383	1.286	1.281
**40S ribosomal protein S16 OS=Mus musculus GN=Rps16 PE=1 SV=4**	1.000	9.121	5.060	8.968	13.442	11.963
**Malate dehydrogenase, cytoplasmic OS=Mus musculus GN=Mdh1 PE=1 SV=3**	0.999	1.032	1.015	1.244	1.353	1.120
**40S ribosomal protein SA OS=Mus musculus GN=Rpsa PE=1 SV=4**	1.017	1.101	1.059	1.419	1.419	1.442
**Calreticulin OS=Mus musculus GN=Calr PE=1 SV=1**	1.177	1.109	1.143	1.167	1.268	1.156
**H-2 class I histocompatibility antigen, D-K alpha chain OS=Mus musculus GN=H2-D1 PE=1 SV=1**	1.000	0.887	0.944	0.642	0.524	0.503
**H-2 class I histocompatibility antigen, Q8 alpha chain OS=Mus musculus GN=H2-Q8 PE=3 SV=1**	1.000	0.992	0.996	0.381	0.356	0.355
**26S proteasome non-ATPase regulatory subunit 3 OS=Mus musculus GN=Psmd3 PE=1 SV=3**	0.992	1.166	1.079	1.305	1.336	1.416
**Interleukin-1 receptor-like 1 OS=Mus musculus GN=Il1rl1 PE=1 SV=2**	1.010	0.898	0.954	1.317	1.309	1.217
**Lamin-B1 OS=Mus musculus GN=Lmnb1 PE=1 SV=3**	0.957	1.048	1.002	1.611	1.519	1.506
**Annexin A6 OS=Mus musculus GN=Anxa6 PE=1 SV=3**	1.000	1.013	1.006	0.711	0.789	0.721
**60S acidic ribosomal protein P0 OS=Mus musculus GN=Rplp0 PE=1 SV=3**	0.982	1.040	1.011	1.646	1.687	1.704
**Heme oxygenase 1 OS=Mus musculus GN=Hmox1**	0.999	1.134	1.066	1.015	0.946	1.149

**PE=1 SV=1**						
**Interferon-activable protein 204 OS=Mus musculus GN=Ifi204 PE=1 SV=2**	1.000	0.801	0.900	0.360	0.748	1.011
**Cadherin-2 OS=Mus musculus GN=Cdh2 PE=1 SV=2**	0.828	0.809	0.819	0.632	0.588	0.603
**CD44 antigen OS=Mus musculus GN=Cd44 PE=1 SV=3**	1.005	1.011	1.008	1.363	1.644	1.374
**Nucleoside diphosphate kinase A OS=Mus musculus GN=Nme1 PE=1 SV=1**	1.046	0.874	0.960	1.864	2.291	1.643
**Glutathione S-transferase Mu 2 OS=Mus musculus GN=Gstm2 PE=1 SV=2**	1.057	0.976	1.017	0.888	0.966	1.084
**Galectin-1 OS=Mus musculus GN=Lgals1 PE=1 SV=3**	1.003	1.017	1.010	1.749	1.726	1.550
**Galectin-3 OS=Mus musculus GN=Lgals3 PE=1 SV=3**	0.997	1.047	1.022	1.589	1.833	1.429
**L-lactate dehydrogenase B chain OS=Mus musculus GN=Ldhb PE=1 SV=2**	1.007	0.953	0.980	0.935	1.028	0.795
**Putative ATP-dependent RNA helicase Pl10 OS=Mus musculus GN=D1Pas1 PE=1 SV=1**	1.000	0.937	0.969	0.912	0.971	1.122
**Spectrin alpha chain, non-erythrocytic 1 OS=Mus musculus GN=Sptan1 PE=1 SV=4**	1.169	1.220	1.195	1.076	1.059	1.130
**Heat shock 70 kDa protein 1-like OS=Mus musculus GN=Hspa1l PE=1 SV=4**	1.000	1.086	1.043	1.147	1.065	1.106
**Lysosomal protective protein OS=Mus musculus GN=Ctsa PE=1 SV=1**	0.958	1.078	1.018	0.793	0.762	0.913
**Glyceraldehyde-3-phosphate dehydrogenase OS=Mus musculus GN=Gapdh PE=1 SV=2**	0.934	1.036	0.985	2.565	2.690	2.495
**Lysosome-associated membrane glycoprotein 2 OS=Mus musculus GN=Lamp2 PE=1 SV=2**	1.024	1.041	1.033	0.641	1.101	0.710
**High mobility group protein HMG-I/HMG-Y OS=Mus musculus GN=Hmga1 PE=1 SV=4**	0.830	0.864	0.847	5.460	6.793	4.577
**Alpha-enolase OS=Mus musculus GN=Eno1 PE=1 SV=3**	1.054	0.985	1.020	2.065	2.200	2.103
**Gamma-enolase OS=Mus musculus GN=Eno2 PE=1 SV=2**	1.038	0.943	0.991	1.155	0.819	1.021
**Polypyrimidine tract-binding protein 1 OS=Mus musculus GN=Ptbp1 PE=1 SV=2**	0.928	0.956	0.942	1.861	2.256	1.705
**AP-2 complex subunit alpha-1 OS=Mus musculus GN=Ap2a1 PE=1 SV=1**	1.000	1.073	1.036	1.639	2.577	2.667
**AP-2 complex subunit alpha-2 OS=Mus musculus GN=Ap2a2 PE=1 SV=2**	1.000	1.088	1.044	1.065	1.075	1.088
**Glucosylceramidase OS=Mus musculus GN=Gba PE=1 SV=1**	0.996	0.965	0.980	1.234	1.482	1.292
**C-X-C motif chemokine 10 OS=Mus musculus GN=Cxcl10 PE=1 SV=1**	1.091	1.144	1.117	0.392	0.351	0.418
**Hexokinase-1 OS=Mus musculus GN=Hk1 PE=1 SV=3**	1.000	0.930	0.965	1.054	0.911	1.196
**Peptidyl-prolyl cis-trans isomerase A OS=Mus musculus GN=Ppia PE=1 SV=2**	1.139	1.084	1.112	1.975	2.008	1.768
**Triosephosphate isomerase OS=Mus musculus GN=Tpi1 PE=1 SV=4**	0.994	0.981	0.987	2.128	2.545	1.749
**Heat shock 70 kDa protein 1B OS=Mus musculus GN=Hspa1b PE=1 SV=3**	1.016	1.036	1.026	1.099	1.352	1.154
**Proliferating cell nuclear antigen OS=Mus musculus GN=Pcna PE=1 SV=2**	0.870	0.895	0.882	1.795	2.136	1.680
**Cathepsin D OS=Mus musculus GN=Ctsd PE=1 SV=1**	1.027	0.997	1.012	0.779	0.640	0.783
**Protein CYR61 OS=Mus musculus GN=Cyr61 PE=1 SV=1**	1.024	1.053	1.038	0.865	1.060	1.040
**Cofilin-1 OS=Mus musculus GN=Cfl1 PE=1 SV=3**	0.954	0.921	0.937	2.032	2.550	1.913
**Fatty acid synthase OS=Mus musculus GN=Fasn**	0.995	0.948	0.972	1.184	1.164	1.219

**PE=1 SV=2**						
**Troponin C, slow skeletal and cardiac muscles OS=Mus musculus GN=Tnnc1 PE=1 SV=1**	1.000	2.028	1.514	0.774	0.540	0.463
**Glutathione S-transferase P 1 OS=Mus musculus GN=Gstp1 PE=1 SV=2**	0.940	0.984	0.962	1.523	1.327	1.452
**Serpin H1 OS=Mus musculus GN=Serpinh1 PE=1 SV=3**	0.975	1.137	1.056	0.788	0.773	0.696
**Negative elongation factor E OS=Mus musculus GN=Nelfe PE=1 SV=2**	1.000	1.588	1.294	2.807	1.911	1.527
**78 kDa glucose-regulated protein OS=Mus musculus GN=Hspa5 PE=1 SV=3**	1.098	1.032	1.065	0.971	0.868	0.933
**Beta-hexosaminidase subunit beta OS=Mus musculus GN=Hexb PE=1 SV=2**	1.000	0.934	0.967	0.693	0.886	0.968
**Thymosin beta-4 OS=Mus musculus GN=Tmsb4x PE=1 SV=1**	1.000	1.380	1.190	2.132	0.958	2.251
**Vimentin OS=Mus musculus GN=Vim PE=1 SV=3**	0.956	1.073	1.015	1.039	1.466	1.047
**Tropomyosin alpha-3 chain OS=Mus musculus GN=Tpm3 PE=1 SV=3**	1.010	1.005	1.008	1.275	1.425	1.173
**Ubiquitin-like protein 4A OS=Mus musculus GN=Ubl4a PE=1 SV=1**	1.000	0.755	0.877	0.571	1.011	0.916
**Cystatin-C OS=Mus musculus GN=Cst3 PE=1 SV=2**	1.111	1.076	1.093	1.044	0.938	1.109
**Beta-enolase OS=Mus musculus GN=Eno3 PE=1 SV=3**	0.996	0.893	0.945	1.130	1.314	1.018
**Lamin-B2 OS=Mus musculus GN=Lmnb2 PE=1 SV=2**	1.000	0.909	0.955	0.700	1.069	0.762
**Lactadherin OS=Mus musculus GN=Mfge8 PE=1 SV=3**	0.951	1.014	0.983	0.676	0.662	0.613
**Histone H2A type 1 OS=Mus musculus GN=Hist1h2ab PE=1 SV=3**	1.000	0.995	0.998	0.354	0.603	0.333
**Plasminogen activator inhibitor 1 OS=Mus musculus GN=Serpine1 PE=1 SV=1**	1.055	1.174	1.115	0.381	0.338	0.363
**AP-1 complex subunit gamma-1 OS=Mus musculus GN=Ap1g1 PE=1 SV=3**	1.000	0.864	0.932	1.080	1.041	0.744
**Eukaryotic translation initiation factor 3 subunit A OS=Mus musculus GN=Eif3a PE=1 SV=5**	1.018	1.108	1.063	1.532	1.400	1.510
**Chromobox protein homolog 3 OS=Mus musculus GN=Cbx3 PE=1 SV=2**	0.959	0.924	0.942	2.006	2.205	1.797
**Putative helicase MOV-10 OS=Mus musculus GN=Mov10 PE=1 SV=2**	0.878	1.129	1.003	1.414	1.448	1.480
**Purine nucleoside phosphorylase OS=Mus musculus GN=Pnp PE=1 SV=2**	1.091	0.921	1.006	0.893	0.625	0.703
**Protein-L-isoaspartate(D-aspartate) O-methyltransferase OS=Mus musculus GN=Pcmt1 PE=1 SV=3**	1.051	1.107	1.079	1.084	1.118	1.347
**GDP-L-fucose synthase OS=Mus musculus GN=Tsta3 PE=1 SV=3**	1.000	0.846	0.923	0.822	0.763	0.866
**Beta-galactosidase OS=Mus musculus GN=Glb1 PE=1 SV=1**	1.000	1.044	1.022	0.975	0.797	0.962
**Branched-chain-amino-acid aminotransferase, cytosolic OS=Mus musculus GN=Bcat1 PE=1 SV=2**	0.941	0.888	0.915	1.659	1.317	1.302
**Peptidyl-prolyl cis-trans isomerase B OS=Mus musculus GN=Ppib PE=1 SV=2**	1.109	1.150	1.130	1.568	1.422	1.440
**Macrophage-capping protein OS=Mus musculus GN=Capg PE=1 SV=2**	1.234	1.285	1.260	1.910	1.798	1.829
**Glutathione S-transferase A4 OS=Mus musculus GN=Gsta4 PE=1 SV=3**	1.000	0.918	0.959	1.578	1.038	1.638
**Leukotriene A-4 hydrolase OS=Mus musculus GN=Lta4h PE=1 SV=4**	0.939	0.879	0.909	1.041	1.145	1.013
**Inosine-5'-monophosphate dehydrogenase 2 OS=Mus musculus GN=Impdh2 PE=1 SV=2**	1.000	1.033	1.016	1.447	1.554	1.624
**Cyclin-dependent kinase 11B OS=Mus musculus**	1.035	0.833	0.934	0.994	1.053	0.938

**GN=Cdk11b PE=1 SV=2**						
**DNA replication licensing factor MCM3 OS=Mus musculus GN=Mcm3 PE=1 SV=2**	1.000	1.007	1.003	0.827	0.834	1.060
**40S ribosomal protein S2 OS=Mus musculus GN=Rps2 PE=1 SV=3**	1.000	1.105	1.053	0.875	0.856	1.049
**Metalloproteinase inhibitor 2 OS=Mus musculus GN=Timp2 PE=1 SV=2**	1.034	0.977	1.005	1.726	1.221	1.210
**Talin-1 OS=Mus musculus GN=Tln1 PE=1 SV=2**	1.117	1.097	1.107	1.528	1.584	1.443
**Ezrin OS=Mus musculus GN=Ezr PE=1 SV=3**	0.981	0.932	0.957	1.200	1.121	1.139
**Moesin OS=Mus musculus GN=Msn PE=1 SV=3**	1.202	1.197	1.200	1.917	2.202	1.704
**Radixin OS=Mus musculus GN=Rdx PE=1 SV=3**	0.975	0.945	0.960	1.223	1.191	1.321
**Catenin alpha-1 OS=Mus musculus GN=Ctnna1 PE=1 SV=1**	0.891	1.361	1.126	2.151	2.090	2.963
**Prothymosin alpha OS=Mus musculus GN=Ptma PE=1 SV=2**	1.000	1.052	1.026	0.588	0.843	0.658
**Splicing factor U2AF 65 kDa subunit OS=Mus musculus GN=U2af2 PE=1 SV=3**	0.997	0.913	0.955	1.254	1.273	1.274
**Glutamate dehydrogenase 1, mitochondrial OS=Mus musculus GN=Glud1 PE=1 SV=1**	1.044	1.077	1.061	0.794	0.973	0.737
**26S proteasome non-ATPase regulatory subunit 7 OS=Mus musculus GN=Psmd7 PE=1 SV=2**	0.994	1.006	1.000	1.110	0.956	1.173
**Platelet-derived growth factor receptor alpha OS=Mus musculus GN=Pdgfra PE=1 SV=3**	1.000	0.830	0.915	0.917	0.894	0.948
**Serine--tRNA ligase, cytoplasmic OS=Mus musculus GN=Sars PE=1 SV=3**	1.016	0.956	0.986	1.206	1.341	1.179
**Myristoylated alanine-rich C-kinase substrate OS=Mus musculus GN=Marcks PE=1 SV=2**	1.089	1.110	1.099	0.825	1.001	0.787
**Peptidyl-prolyl cis-trans isomerase FKBP1A OS=Mus musculus GN=Fkbp1a PE=1 SV=2**	1.018	1.042	1.030	1.346	1.242	1.416
**Alpha-mannosidase 2 OS=Mus musculus GN=Man2a1 PE=1 SV=2**	1.129	1.087	1.108	0.818	0.774	0.879
**Microtubule-associated protein 4 OS=Mus musculus GN=Map4 PE=1 SV=3**	0.952	0.942	0.947	0.893	1.000	1.056
**Phospholipase A-2-activating protein OS=Mus musculus GN=Plaa PE=1 SV=4**	0.987	0.912	0.949	1.171	1.123	1.292
**60S ribosomal protein L3 OS=Mus musculus GN=Rpl3 PE=1 SV=3**	1.000	0.917	0.959	0.715	0.935	0.631
**Protein disulfide-isomerase A3 OS=Mus musculus GN=Pdia3 PE=1 SV=2**	1.116	1.006	1.061	1.131	1.049	0.977
**Protein-lysine 6-oxidase OS=Mus musculus GN=Lox PE=1 SV=1**	1.009	1.398	1.203	1.177	1.014	1.093
**DNA-(apurinic or apyrimidinic site) lyase OS=Mus musculus GN=Apex1 PE=1 SV=2**	1.005	1.127	1.066	2.410	2.652	2.299
**Alcohol dehydrogenase class-3 OS=Mus musculus GN=Adh5 PE=1 SV=3**	1.077	1.202	1.139	1.715	2.157	1.887
**Collagen alpha-1(II) chain OS=Mus musculus GN=Col2a1 PE=1 SV=2**	1.000	0.996	0.998	0.811	0.780	0.596
**Biglycan OS=Mus musculus GN=Bgn PE=1 SV=1**	0.977	0.939	0.958	0.702	0.644	0.667
**Decorin OS=Mus musculus GN=Dcn PE=1 SV=1**	0.991	0.997	0.994	1.199	1.329	1.174
**Nucleosome assembly protein 1-like 1 OS=Mus musculus GN=Nap1l1 PE=1 SV=2**	0.862	0.981	0.921	1.881	2.159	1.544
**MARCKS-related protein OS=Mus musculus GN=Marcksl1 PE=1 SV=2**	1.153	1.379	1.266	1.620	1.940	2.062
**Granulins OS=Mus musculus GN=Grn PE=1 SV=2**	1.006	1.030	1.018	0.554	0.479	0.558
**Stromelysin-1 OS=Mus musculus GN=Mmp3 PE=2 SV=2**	1.049	1.216	1.132	0.557	0.473	0.622
**Connective tissue growth factor OS=Mus musculus GN=Ctgf PE=2 SV=3**	1.000	1.017	1.009	0.555	0.465	0.508
**Polyadenylate-binding protein 1 OS=Mus musculus GN=Pabpc1 PE=1 SV=2**	0.961	0.952	0.956	1.180	1.228	1.144

**Ferritin light chain 1 OS=Mus musculus GN=Ftl1 PE=1 SV=2**	0.975	0.884	0.930	0.803	0.891	0.674
**Beta-hexosaminidase subunit alpha OS=Mus musculus GN=Hexa PE=1 SV=2**	1.037	1.089	1.063	1.046	1.101	0.928
**Vascular cell adhesion protein 1 OS=Mus musculus GN=Vcam1 PE=1 SV=1**	1.038	1.042	1.040	0.242	0.191	0.245
**NEDD8 OS=Mus musculus GN=Nedd8 PE=1 SV=2**	1.000	0.956	0.978	1.302	1.221	1.748
**Alpha-2-HS-glycoprotein OS=Mus musculus GN=Ahsg PE=1 SV=1**	1.030	1.031	1.031	1.327	0.976	1.513
**Ornithine aminotransferase, mitochondrial OS=Mus musculus GN=Oat PE=1 SV=1**	1.000	1.010	1.005	0.577	0.962	0.623
**Cyclin-dependent kinase 4 OS=Mus musculus GN=Cdk4 PE=1 SV=1**	1.000	1.017	1.009	0.799	0.798	0.913
**Peptidyl-prolyl cis-trans isomerase C OS=Mus musculus GN=Ppic PE=1 SV=1**	0.975	0.993	0.984	1.054	0.983	1.101
**Peptidyl-prolyl cis-trans isomerase FKBP4 OS=Mus musculus GN=Fkbp4 PE=1 SV=5**	0.965	1.218	1.091	1.567	1.571	1.426
**High mobility group protein B2 OS=Mus musculus GN=Hmgb2 PE=1 SV=3**	1.002	1.125	1.064	3.373	4.234	3.479
**C-C motif chemokine 5 OS=Mus musculus GN=Ccl5 PE=2 SV=2**	1.000	0.894	0.947	0.850	0.787	0.411
**Aminoacyl tRNA synthase complex-interacting multifunctional protein 1 OS=Mus musculus GN=Aimp1 PE=1 SV=2**	1.000	0.966	0.983	1.229	1.018	1.188
**cAMP-dependent protein kinase type II-beta regulatory subunit OS=Mus musculus GN=Prkar2b PE=1 SV=3**	0.922	0.912	0.917	1.267	1.058	1.248
**Acyl-CoA-binding protein OS=Mus musculus GN=Dbi PE=1 SV=2**	0.946	0.985	0.965	1.078	1.292	0.991
**Lupus La protein homolog OS=Mus musculus GN=Ssb PE=1 SV=1**	1.000	1.125	1.062	0.731	1.101	0.819
**Antithrombin-III OS=Mus musculus GN=Serpinc1 PE=1 SV=1**	0.984	0.939	0.962	0.930	0.833	0.877
**Nectin-2 OS=Mus musculus GN=Pvrl2 PE=1 SV=2**	1.000	1.077	1.038	0.834	0.744	0.799
**Cytochrome P450 2F2 OS=Mus musculus GN=Cyp2f2 PE=1 SV=1**	1.000	0.942	0.971	0.966	0.581	0.972
**72 kDa type IV collagenase OS=Mus musculus GN=Mmp2 PE=1 SV=1**	0.940	0.914	0.927	0.320	0.222	0.297
**Ran-specific GTPase-activating protein OS=Mus musculus GN=Ranbp1 PE=1 SV=2**	0.963	0.918	0.941	1.491	1.773	1.347
**Focal adhesion kinase 1 OS=Mus musculus GN=Ptk2 PE=1 SV=3**	1.000	1.515	1.257	1.648	1.528	1.698
**Macrophage migration inhibitory factor OS=Mus musculus GN=Mif PE=1 SV=2**	0.976	1.011	0.994	2.518	2.393	1.630
**Tyrosine-protein phosphatase non-receptor type 11 OS=Mus musculus GN=Ptpn11 PE=1 SV=2**	1.000	1.166	1.083	3.171	2.540	2.442
**Ras-related protein Rab-5C OS=Mus musculus GN=Rab5c PE=1 SV=2**	1.000	0.881	0.940	0.856	0.704	0.861
**Thrombospondin-1 OS=Mus musculus GN=Thbs1 PE=1 SV=1**	0.957	0.945	0.951	0.731	0.772	0.712
**rRNA 2'-O-methyltransferase fibrillarin OS=Mus musculus GN=Fbl PE=1 SV=2**	1.000	1.036	1.018	0.812	0.838	1.511
**Calnexin OS=Mus musculus GN=Canx PE=1 SV=1**	1.055	1.086	1.071	0.559	0.643	0.644
**AP-1 complex subunit mu-1 OS=Mus musculus GN=Ap1m1 PE=1 SV=3**	1.000	0.982	0.991	0.751	0.906	0.814
**Peroxiredoxin-1 OS=Mus musculus GN=Prdx1 PE=1 SV=1**	1.058	1.122	1.090	1.798	1.872	1.631
**60S ribosomal protein L12 OS=Mus musculus GN=Rpl12 PE=1 SV=2**	1.043	1.015	1.029	1.093	1.068	1.278
**Transgelin OS=Mus musculus GN=Tagln PE=1 SV=3**	0.995	1.118	1.056	0.680	0.825	0.667

**Fibulin-2 OS=Mus musculus GN=Fbln2 PE=1 SV=2**	0.990	1.031	1.011	0.669	0.739	0.726
**Stress-70 protein, mitochondrial OS=Mus musculus GN=Hspa9 PE=1 SV=3**	1.029	0.990	1.009	0.841	0.981	0.799
**Dynamin-2 OS=Mus musculus GN=Dnm2 PE=1 SV=2**	1.000	1.058	1.029	1.083	0.989	1.505
**Tight junction protein ZO-1 OS=Mus musculus GN=Tjp1 PE=1 SV=2**	1.000	0.858	0.929	0.598	0.950	0.997
**Adenylyl cyclase-associated protein 1 OS=Mus musculus GN=Cap1 PE=1 SV=4**	0.944	0.911	0.927	0.917	0.989	1.043
**Transketolase OS=Mus musculus GN=Tkt PE=1 SV=1**	1.113	1.214	1.163	1.713	2.434	1.737
**Vacuolar protein sorting-associated protein 26A OS=Mus musculus GN=Vps26a PE=1 SV=1**	1.000	1.035	1.018	1.309	1.048	1.061
**DNA-binding protein SMUBP-2 OS=Mus musculus GN=Ighmbp2 PE=1 SV=1**	1.000	0.872	0.936	1.195	1.016	2.075
**CD63 antigen OS=Mus musculus GN=Cd63 PE=1 SV=2**	1.000	0.967	0.984	0.636	0.736	0.710
**Enoyl-CoA delta isomerase 1, mitochondrial OS=Mus musculus GN=Eci1 PE=1 SV=2**	0.966	0.825	0.895	0.457	0.688	0.490
**Signal transducer and activator of transcription 3 OS=Mus musculus GN=Stat3 PE=1 SV=2**	1.000	0.890	0.945	1.087	0.807	1.066
**Epidermal growth factor receptor substrate 15 OS=Mus musculus GN=Eps15 PE=1 SV=1**	1.000	1.173	1.086	0.899	0.831	1.290
**Transcriptional activator protein Pur-alpha OS=Mus musculus GN=Pura PE=1 SV=1**	1.000	0.794	0.897	0.996	0.699	0.748
**T-complex protein 1 subunit theta OS=Mus musculus GN=Cct8 PE=1 SV=3**	1.044	1.046	1.045	1.289	1.315	1.384
**Growth/differentiation factor 6 OS=Mus musculus GN=Gdf6 PE=2 SV=2**	1.000	1.011	1.006	0.289	0.211	0.364
**Aldose reductase OS=Mus musculus GN=Akr1b1 PE=1 SV=3**	0.910	0.927	0.919	2.234	2.847	2.271
**Aldose reductase-related protein 2 OS=Mus musculus GN=Akr1b8 PE=1 SV=2**	1.011	1.191	1.101	3.652	3.352	2.832
**Cofilin-2 OS=Mus musculus GN=Cfl2 PE=1 SV=1**	0.983	0.952	0.967	1.150	1.226	0.962
**Mannosyl-oligosaccharide 1,2-alpha-mannosidase IA OS=Mus musculus GN=Man1a1 PE=1 SV=1**	1.118	0.931	1.024	0.442	0.416	0.509
**Peptidyl-prolyl cis-trans isomerase FKBP2 OS=Mus musculus GN=Fkbp2 PE=1 SV=1**	0.952	0.885	0.918	0.792	0.850	0.826
**Medium-chain specific acyl-CoA dehydrogenase, mitochondrial OS=Mus musculus GN=Acadm PE=1 SV=1**	1.000	0.927	0.964	0.690	0.527	1.087
**Ran GTPase-activating protein 1 OS=Mus musculus GN=Rangap1 PE=1 SV=2**	0.997	1.060	1.029	1.516	1.351	1.370
**26S protease regulatory subunit 7 OS=Mus musculus GN=Psmc2 PE=1 SV=5**	1.122	1.079	1.101	1.384	1.463	1.280
**Ras-related protein Rab-11B OS=Mus musculus GN=Rab11b PE=1 SV=3**	1.000	0.981	0.991	1.035	0.981	1.303
**Adenylosuccinate synthetase isozyme 2 OS=Mus musculus GN=Adss PE=1 SV=2**	0.849	0.896	0.872	1.013	0.991	1.095
**E3 ubiquitin-protein ligase NEDD4 OS=Mus musculus GN=Nedd4 PE=1 SV=3**	0.994	1.117	1.056	1.068	0.953	0.961
**Quinone oxidoreductase OS=Mus musculus GN=Cryz PE=1 SV=1**	1.000	1.036	1.018	0.906	1.209	1.027
**Aldehyde dehydrogenase, mitochondrial OS=Mus musculus GN=Aldh2 PE=1 SV=1**	1.000	1.089	1.045	0.936	0.900	0.851
**Aldehyde dehydrogenase, dimeric NADP-preferring OS=Mus musculus GN=Aldh3a1 PE=1 SV=2**	0.947	0.906	0.927	1.457	1.520	1.327
**F-actin-capping protein subunit alpha-1 OS=Mus musculus GN=Capza1 PE=1 SV=4**	1.016	1.041	1.029	1.592	1.448	1.753
**F-actin-capping protein subunit alpha-2 OS=Mus**	0.938	0.902	0.920	0.991	0.942	0.892

musculus GN=Capza2 PE=1 SV=3						
F-actin-capping protein subunit beta OS=Mus musculus GN=Capzb PE=1 SV=3	1.038	1.101	1.069	1.187	1.625	1.207
Glutathione reductase, mitochondrial OS=Mus musculus GN=Gsr PE=1 SV=3	0.954	1.398	1.176	1.870	1.717	1.781
Insulin-like growth factor-binding protein 2 OS=Mus musculus GN=Igfbp2 PE=2 SV=2	0.969	0.920	0.945	0.228	0.247	0.338
Insulin-like growth factor-binding protein 3 OS=Mus musculus GN=Igfbp3 PE=2 SV=2	0.981	1.014	0.998	0.632	0.511	0.659
Insulin-like growth factor-binding protein 4 OS=Mus musculus GN=Igfbp4 PE=1 SV=2	0.971	1.066	1.018	1.866	1.468	1.574
Insulin-like growth factor-binding protein 6 OS=Mus musculus GN=Igfbp6 PE=2 SV=2	0.926	0.934	0.930	2.379	2.703	1.598
Crk-like protein OS=Mus musculus GN=Crkl PE=1 SV=2	1.000	0.956	0.978	1.246	1.269	1.008
60S acidic ribosomal protein P1 OS=Mus musculus GN=Rplp1 PE=1 SV=1	0.992	1.058	1.025	1.666	1.769	1.709
60S ribosomal protein L5 OS=Mus musculus GN=Rpl5 PE=1 SV=3	1.000	1.021	1.011	0.762	0.810	0.735
Eukaryotic translation initiation factor 1 OS=Mus musculus GN=Eif1 PE=1 SV=2	1.000	0.835	0.918	0.727	0.914	0.680
Annexin A5 OS=Mus musculus GN=Anxa5 PE=1 SV=1	1.120	1.312	1.216	1.251	1.655	1.098
Protein 4.1 OS=Mus musculus GN=Epb41 PE=1 SV=2	1.000	0.692	0.846	1.278	0.404	0.605
Tubulin-specific chaperone A OS=Mus musculus GN=Tbca PE=1 SV=3	1.000	1.309	1.154	1.941	1.727	1.764
Prelamin-A/C OS=Mus musculus GN=Lmna PE=1 SV=2	1.018	1.011	1.014	1.311	1.431	1.292
Heat shock 70 kDa protein 4L OS=Mus musculus GN=Hspa4l PE=1 SV=2	1.000	0.976	0.988	0.568	0.559	0.920
Carbonyl reductase [NADPH] 1 OS=Mus musculus GN=Cbr1 PE=1 SV=3	1.000	0.903	0.951	0.929	0.730	0.672
Pentraxin-related protein PTX3 OS=Mus musculus GN=Ptx3 PE=1 SV=2	1.035	1.045	1.040	0.342	0.266	0.302
Glutathione S-transferase Mu 5 OS=Mus musculus GN=Gstm5 PE=1 SV=1	1.000	0.959	0.980	1.342	1.023	1.705
Heterogeneous nuclear ribonucleoprotein A1 OS=Mus musculus GN=Hnrnpa1 PE=1 SV=2	1.203	1.187	1.195	1.863	1.986	1.714
Protein phosphatase 1A OS=Mus musculus GN=Ppm1a PE=1 SV=1	0.936	0.845	0.891	0.857	0.840	1.109
DNA replication licensing factor MCM4 OS=Mus musculus GN=Mcm4 PE=1 SV=1	1.000	0.884	0.942	0.851	0.874	0.812
DNA replication licensing factor MCM5 OS=Mus musculus GN=Mcm5 PE=1 SV=1	1.000	1.010	1.005	0.719	0.685	0.817
Proteasome subunit alpha type-2 OS=Mus musculus GN=Psma2 PE=1 SV=3	0.928	0.905	0.916	1.145	1.370	1.367
C-X-C motif chemokine 5 OS=Mus musculus GN=Cxcl5 PE=1 SV=2	1.029	1.094	1.061	2.457	2.365	1.926
Adenosylhomocysteinase OS=Mus musculus GN=Ahcy PE=1 SV=3	1.124	1.160	1.142	1.539	2.048	1.748
Rab GDP dissociation inhibitor alpha OS=Mus musculus GN=Gdi1 PE=1 SV=3	1.000	0.946	0.973	1.204	1.403	1.109
Arylsulfatase A OS=Mus musculus GN=Arsa PE=1 SV=2	1.000	0.971	0.985	0.890	0.858	0.873
Keratin, type II cytoskeletal 6A OS=Mus musculus GN=Krt6a PE=1 SV=3	1.030	0.873	0.952	0.531	0.644	0.625
V-type proton ATPase catalytic subunit A OS=Mus musculus GN=Atp6v1a PE=1 SV=2	0.906	1.014	0.960	0.871	1.109	0.944
Protein S100-A11 OS=Mus musculus GN=S100a11 PE=1 SV=1	1.294	1.656	1.475	1.948	2.012	1.658

**Proliferation-associated protein 2G4 OS=Mus musculus GN=Pa2g4 PE=1 SV=3**	0.871	0.845	0.858	1.286	1.666	1.326
**Fibromodulin OS=Mus musculus GN=Fmod PE=2 SV=1**	1.000	0.948	0.974	0.526	0.590	1.140
**Calpastatin OS=Mus musculus GN=Cast PE=1 SV=2**	1.073	1.026	1.049	1.345	1.193	1.042
**Ras-related protein Rab-7a OS=Mus musculus GN=Rab7a PE=1 SV=2**	0.991	1.058	1.024	1.079	1.070	1.156
**Alpha-galactosidase A OS=Mus musculus GN=Gla PE=1 SV=1**	1.000	1.055	1.027	1.252	1.121	1.413
**Glypican-4 OS=Mus musculus GN=Gpc4 PE=1 SV=2**	1.000	0.996	0.998	0.550	0.373	0.614
**Glutathione synthetase OS=Mus musculus GN=Gss PE=1 SV=1**	1.000	1.010	1.005	1.374	1.061	1.567
**Hepatoma-derived growth factor OS=Mus musculus GN=Hdgf PE=1 SV=2**	1.034	0.968	1.001	1.976	2.249	1.793
**Lumican OS=Mus musculus GN=Lum PE=1 SV=2**	0.955	0.929	0.942	0.903	0.698	1.023
**Importin subunit alpha-1 OS=Mus musculus GN=Kpna2 PE=1 SV=2**	0.992	1.000	0.996	1.250	1.357	1.498
**Pyruvate kinase PKM OS=Mus musculus GN=Pkm PE=1 SV=4**	1.145	1.122	1.133	2.471	2.610	2.304
**Ribonuclease UK114 OS=Mus musculus GN=Hrsp12 PE=1 SV=3**	1.000	0.807	0.904	0.966	0.786	1.079
**Nucleolysin TIA-1 OS=Mus musculus GN=Tia1 PE=1 SV=1**	1.000	0.828	0.914	0.558	0.451	0.498
**High mobility group protein HMGI-C OS=Mus musculus GN=Hmga2 PE=1 SV=1**	0.700	0.623	0.662	5.286	6.072	3.564
**60S ribosomal protein L10a OS=Mus musculus GN=Rpl10a PE=1 SV=3**	1.070	1.140	1.105	1.033	1.363	1.224
**Matrix metalloproteinase-14 OS=Mus musculus GN=Mmp14 PE=2 SV=3**	1.000	1.066	1.033	0.930	0.520	1.098
**Phosphatidylinositol transfer protein alpha isoform OS=Mus musculus GN=Pitpna PE=1 SV=2**	0.906	0.913	0.910	1.471	1.642	1.355
**Phosphatidylinositol transfer protein beta isoform OS=Mus musculus GN=Pitpnb PE=1 SV=2**	1.000	0.936	0.968	0.798	1.002	0.855
**Ras-related protein Rab-2A OS=Mus musculus GN=Rab2a PE=1 SV=1**	1.000	0.860	0.930	0.414	0.468	0.583
**Cellular nucleic acid-binding protein OS=Mus musculus GN=Cnbp PE=1 SV=2**	1.000	0.930	0.965	1.662	1.946	1.414
**Stathmin OS=Mus musculus GN=Stmn1 PE=1 SV=2**	1.000	0.835	0.917	2.007	2.341	2.035
**Elastin OS=Mus musculus GN=Eln PE=1 SV=2**	0.942	1.083	1.012	0.360	0.299	0.417
**UV excision repair protein RAD23 homolog A OS=Mus musculus GN=Rad23a PE=1 SV=2**	0.951	2.176	1.563	4.032	3.879	3.498
**UV excision repair protein RAD23 homolog B OS=Mus musculus GN=Rad23b PE=1 SV=2**	1.058	1.061	1.059	1.514	1.687	1.470
**26S protease regulatory subunit 6B OS=Mus musculus GN=Psmc4 PE=1 SV=2**	1.000	0.805	0.903	0.602	1.103	0.808
**Adenylosuccinate lyase OS=Mus musculus GN=Adsl PE=1 SV=2**	1.000	0.900	0.950	1.094	1.032	1.014
**Probable ATP-dependent RNA helicase DDX6 OS=Mus musculus GN=Ddx6 PE=1 SV=1**	1.000	0.994	0.997	1.649	1.450	1.568
**[Protein ADP-ribosylarginine] hydrolase OS=Mus musculus GN=Adprh PE=1 SV=1**	1.000	0.970	0.985	0.917	0.759	1.063
**Microfibrillar-associated protein 2 OS=Mus musculus GN=Mfap2 PE=2 SV=1**	1.000	0.746	0.873	1.207	0.585	0.719
**Phospholipid transfer protein OS=Mus musculus GN=Pltp PE=1 SV=1**	1.000	1.158	1.079	1.049	0.745	0.883
**Adenosine kinase OS=Mus musculus GN=Adk PE=1 SV=2**	1.000	1.164	1.082	1.467	1.426	1.620
**Cadherin-11 OS=Mus musculus GN=Cdh11 PE=1 SV=1**	1.000	0.896	0.948	1.125	0.960	0.953
**Acylphosphatase-2 OS=Mus musculus GN=Acyp2 PE=1 SV=2**	1.059	1.178	1.119	1.149	1.443	1.258

**Acylphosphatase-1 OS=Mus musculus GN=Acyp1 PE=1 SV=2**	1.000	1.146	1.073	1.399	1.788	1.528
**Cytochrome c oxidase subunit 6B1 OS=Mus musculus GN=Cox6b1 PE=1 SV=2**	1.000	0.900	0.950	1.155	0.833	0.939
**Cytochrome b5 OS=Mus musculus GN=Cyb5a PE=1 SV=2**	1.000	1.143	1.072	1.254	1.440	0.767
**Ubiquitin carboxyl-terminal hydrolase 5 OS=Mus musculus GN=Usp5 PE=1 SV=1**	1.060	0.962	1.011	1.328	1.330	1.216
**ATP synthase subunit beta, mitochondrial OS=Mus musculus GN=Atp5b PE=1 SV=2**	0.954	0.993	0.974	0.632	0.722	0.677
**Protein S100-A1 OS=Mus musculus GN=S100a1 PE=1 SV=2**	0.035	15.223	7.629	16.190	14.642	15.007
**Programmed cell death protein 5 OS=Mus musculus GN=Pdcd5 PE=1 SV=3**	0.984	0.988	0.986	1.682	1.689	1.372
**Sjoegren syndrome/scleroderma autoantigen 1 homolog OS=Mus musculus GN=Sssca1 PE=1 SV=1**	1.000	1.029	1.015	1.502	2.811	2.157
**RNA-binding protein FUS OS=Mus musculus GN=Fus PE=1 SV=1**	1.000	1.025	1.013	1.069	1.450	0.975
**Endoplasmic reticulum resident protein 29 OS=Mus musculus GN=Erp29 PE=1 SV=2**	1.000	0.911	0.956	0.628	0.827	0.852
**Elongation factor 1-delta OS=Mus musculus GN=Eef1d PE=1 SV=3**	1.032	0.987	1.010	1.363	1.550	1.344
**Alpha-actinin-4 OS=Mus musculus GN=Actn4 PE=1 SV=1**	1.016	1.047	1.031	1.402	1.429	1.193
**Lysyl oxidase homolog 2 OS=Mus musculus GN=Loxl2 PE=1 SV=2**	0.967	0.886	0.926	0.515	0.383	0.490
**Elongation factor 2 OS=Mus musculus GN=Eef2 PE=1 SV=2**	1.042	1.114	1.078	1.891	1.787	1.738
**Serine/threonine-protein phosphatase 2A activator OS=Mus musculus GN=Ppp2r4 PE=1 SV=1**	0.996	0.939	0.968	0.946	0.864	0.907
**Tropomyosin alpha-1 chain OS=Mus musculus GN=Tpm1 PE=1 SV=1**	0.869	0.922	0.895	1.185	1.196	1.103
**Tropomyosin beta chain OS=Mus musculus GN=Tpm2 PE=1 SV=1**	0.943	1.049	0.996	1.376	1.452	1.327
**182 kDa tankyrase-1-binding protein OS=Mus musculus GN=Tnks1bp1 PE=1 SV=2**	1.016	1.027	1.022	0.924	1.130	1.021
**Eukaryotic translation initiation factor 5 OS=Mus musculus GN=Eif5 PE=1 SV=1**	1.028	0.919	0.974	0.829	0.950	0.949
**Actin-related protein 2/3 complex subunit 4 OS=Mus musculus GN=Arpc4 PE=1 SV=3**	1.000	1.165	1.082	1.253	1.015	1.249
**RuvB-like 1 OS=Mus musculus GN=Ruvbl1 PE=1 SV=1**	1.037	0.913	0.975	0.992	1.116	1.001
**Eukaryotic translation initiation factor 3 subunit E OS=Mus musculus GN=Eif3e PE=1 SV=1**	1.000	1.036	1.018	0.803	0.829	1.060
**Poly(rC)-binding protein 1 OS=Mus musculus GN=Pcbp1 PE=1 SV=1**	0.963	1.157	1.060	2.250	2.236	2.093
**Gamma-aminobutyric acid receptor-associated protein-like 2 OS=Mus musculus GN=Gabarapl2 PE=1 SV=1**	1.000	0.993	0.996	0.621	0.731	0.669
**Nuclear protein localization protein 4 homolog OS=Mus musculus GN=Nploc4 PE=1 SV=3**	1.000	0.926	0.963	0.901	0.802	1.173
**Actin, cytoplasmic 1 OS=Mus musculus GN=Actb PE=1 SV=1**	1.027	1.008	1.018	1.223	1.019	1.174
**Cell division control protein 42 homolog OS=Mus musculus GN=Cdc42 PE=1 SV=2**	0.962	0.935	0.949	0.810	0.902	1.088
**Cold-inducible RNA-binding protein OS=Mus musculus GN=Cirbp PE=1 SV=1**	1.000	1.164	1.082	1.909	1.781	1.417
**Eukaryotic initiation factor 4A-I OS=Mus musculus GN=Eif4a1 PE=1 SV=1**	1.059	1.111	1.085	1.324	1.434	1.372
**40S ribosomal protein S20 OS=Mus musculus**	1.000	1.076	1.038	2.349	1.978	2.453

**GN=Rps20 PE=1 SV=1**						
**Ubiquitin-conjugating enzyme E2 D3 OS=Mus musculus GN=Ube2d3 PE=1 SV=1**	1.000	0.971	0.985	2.801	2.468	2.508
**Ubiquitin-conjugating enzyme E2 K OS=Mus musculus GN=Ube2k PE=1 SV=3**	1.213	1.036	1.125	1.276	1.162	1.600
**Ubiquitin-conjugating enzyme E2 N OS=Mus musculus GN=Ube2n PE=1 SV=1**	1.007	1.013	1.010	1.302	1.463	1.415
**Actin-related protein 2 OS=Mus musculus GN=Actr2 PE=1 SV=1**	1.000	0.959	0.979	1.048	1.166	1.070
**Alpha-centractin OS=Mus musculus GN=Actr1a PE=1 SV=1**	1.015	1.385	1.200	1.513	1.246	1.221
**COP9 signalosome complex subunit 2 OS=Mus musculus GN=Cops2 PE=1 SV=1**	1.000	0.897	0.949	1.696	0.700	0.768
**ADP-ribosylation factor 3 OS=Mus musculus GN=Arf3 PE=2 SV=2**	0.961	1.035	0.998	1.102	1.163	1.256
**ATP-binding cassette sub-family E member 1 OS=Mus musculus GN=Abce1 PE=1 SV=1**	1.000	0.884	0.942	0.355	0.575	0.378
**Ras-related protein Rap-2b OS=Mus musculus GN=Rap2b PE=1 SV=1**	1.000	0.998	0.999	1.109	0.797	1.158
**Proteasome activator complex subunit 3 OS=Mus musculus GN=Psme3 PE=1 SV=1**	0.922	0.990	0.956	1.113	1.027	1.045
**N-alpha-acetyltransferase 20 OS=Mus musculus GN=Naa20 PE=1 SV=1**	1.000	0.909	0.954	0.858	0.586	1.080
**Prefoldin subunit 3 OS=Mus musculus GN=Vbp1 PE=1 SV=2**	1.008	0.914	0.961	1.157	1.299	1.121
**Coatomer subunit zeta-1 OS=Mus musculus GN=Copz1 PE=1 SV=1**	1.000	1.011	1.006	0.973	1.074	1.408
**Small ubiquitin-related modifier 2 OS=Mus musculus GN=Sumo2 PE=1 SV=1**	1.000	0.894	0.947	1.454	1.689	1.619
**Ubiquitin-fold modifier 1 OS=Mus musculus GN=Ufm1 PE=1 SV=1**	1.000	1.088	1.044	1.136	1.260	1.197
**Nuclear transport factor 2 OS=Mus musculus GN=Nutf2 PE=1 SV=1**	1.111	1.172	1.142	1.462	1.782	1.333
**Heterogeneous nuclear ribonucleoprotein K OS=Mus musculus GN=Hnrnpk PE=1 SV=1**	1.147	1.159	1.153	1.702	1.730	1.400
**14-3-3 protein gamma OS=Mus musculus GN=Ywhag PE=1 SV=2**	0.991	1.016	1.003	1.084	1.125	1.101
**Mitochondrial import inner membrane translocase subunit Tim13 OS=Mus musculus GN=Timm13 PE=1 SV=1**	1.036	0.992	1.014	1.275	1.176	1.298
**40S ribosomal protein S7 OS=Mus musculus GN=Rps7 PE=2 SV=1**	2.699	2.910	2.804	2.029	2.809	2.686
**Serine/threonine-protein phosphatase PP1-alpha catalytic subunit OS=Mus musculus GN=Ppp1ca PE=1 SV=1**	1.012	1.096	1.054	1.598	1.864	1.582
**26S protease regulatory subunit 4 OS=Mus musculus GN=Psmc1 PE=1 SV=1**	0.947	0.982	0.964	1.195	1.231	1.232
**26S protease regulatory subunit 8 OS=Mus musculus GN=Psmc5 PE=1 SV=1**	1.000	0.923	0.961	0.742	0.938	0.823
**Calmodulin OS=Mus musculus GN=Calm1 PE=1 SV=2**	0.913	0.940	0.927	1.265	1.541	1.151
**40S ribosomal protein S8 OS=Mus musculus GN=Rps8 PE=1 SV=2**	1.000	0.809	0.905	0.444	1.179	0.568
**Ubiquitin-conjugating enzyme E2 H OS=Mus musculus GN=Ube2h PE=1 SV=1**	1.000	0.912	0.956	0.878	1.173	1.443
**14-3-3 protein epsilon OS=Mus musculus GN=Ywhae PE=1 SV=1**	1.046	1.086	1.066	1.153	1.174	1.113
**40S ribosomal protein S14 OS=Mus musculus GN=Rps14 PE=1 SV=3**	1.003	0.924	0.964	0.781	0.747	0.655
**Small nuclear ribonucleoprotein E OS=Mus musculus GN=Snrpe PE=1 SV=1**	0.998	0.830	0.914	0.617	0.729	0.847

**Small nuclear ribonucleoprotein F OS=Mus musculus GN=Snrpf PE=1 SV=1**	1.000	0.839	0.920	0.909	0.815	0.705
**Small nuclear ribonucleoprotein G OS=Mus musculus GN=Snrpg PE=1 SV=1**	1.000	0.717	0.859	0.459	0.548	0.782
**U6 snRNA-associated Sm-like protein LSm3 OS=Mus musculus GN=Lsm3 PE=1 SV=2**	1.000	0.861	0.930	1.026	1.077	0.953
**Small nuclear ribonucleoprotein Sm D1 OS=Mus musculus GN=Snrpd1 PE=1 SV=1**	1.000	1.036	1.018	1.508	1.096	1.250
**Small nuclear ribonucleoprotein Sm D2 OS=Mus musculus GN=Snrpd2 PE=1 SV=1**	1.009	0.970	0.989	0.740	0.743	0.775
**Small nuclear ribonucleoprotein Sm D3 OS=Mus musculus GN=Snrpd3 PE=1 SV=1**	1.000	0.979	0.989	1.058	1.041	0.872
**26S protease regulatory subunit 10B OS=Mus musculus GN=Psmc6 PE=1 SV=1**	0.942	0.811	0.877	0.837	0.788	0.837
**TSC22 domain family protein 1 OS=Mus musculus GN=Tsc22d1 PE=1 SV=2**	1.000	1.394	1.197	1.392	1.150	1.523
**Dynein light chain roadblock-type 1 OS=Mus musculus GN=Dynlrb1 PE=1 SV=3**	0.953	0.981	0.967	0.972	0.955	0.964
**40S ribosomal protein S4, X isoform OS=Mus musculus GN=Rps4x PE=1 SV=2**	1.000	0.992	0.996	0.465	0.979	0.960
**AP-2 complex subunit sigma OS=Mus musculus GN=Ap2s1 PE=1 SV=1**	1.000	1.112	1.056	1.427	1.354	1.337
**60S ribosomal protein L23a OS=Mus musculus GN=Rpl23a PE=1 SV=1**	1.000	0.952	0.976	1.093	2.344	1.459
**40S ribosomal protein S6 OS=Mus musculus GN=Rps6 PE=1 SV=1**	1.000	0.868	0.934	0.556	1.017	1.030
**Myotrophin OS=Mus musculus GN=Mtpn PE=1 SV=2**	1.000	0.941	0.971	1.980	2.335	1.676
**Histone H4 OS=Mus musculus GN=Hist1h4a PE=1 SV=2**	1.000	0.906	0.953	0.490	0.824	0.611
**V-type proton ATPase subunit B, brain isoform OS=Mus musculus GN=Atp6v1b2 PE=1 SV=1**	1.011	0.939	0.975	0.679	0.849	0.810
**Ras-related protein Rab-1A OS=Mus musculus GN=Rab1A PE=1 SV=3**	1.000	0.879	0.939	0.993	1.198	0.983
**GTP-binding nuclear protein Ran OS=Mus musculus GN=Ran PE=1 SV=3**	0.918	0.882	0.900	1.330	1.563	1.333
**60S ribosomal protein L23 OS=Mus musculus GN=Rpl23 PE=1 SV=1**	1.000	0.762	0.881	0.759	0.743	0.921
**40S ribosomal protein S24 OS=Mus musculus GN=Rps24 PE=1 SV=1**	1.000	0.761	0.881	0.409	0.418	0.359
**40S ribosomal protein S28 OS=Mus musculus GN=Rps28 PE=1 SV=1**	1.004	0.945	0.974	1.479	1.770	1.338
**Transcription elongation factor B polypeptide 2 OS=Mus musculus GN=Tceb2 PE=1 SV=1**	0.961	1.019	0.990	1.918	2.930	2.701
**Guanine nucleotide-binding protein G(I)/G(S)/G(T) subunit beta-2 OS=Mus musculus GN=Gnb2 PE=1 SV=3**	1.636	1.739	1.687	2.077	2.179	1.763
**Cytochrome c, somatic OS=Mus musculus GN=Cycs PE=1 SV=2**	1.120	1.084	1.102	1.670	2.063	1.633
**60S ribosomal protein L31 OS=Mus musculus GN=Rpl31 PE=1 SV=1**	1.000	1.001	1.001	0.862	1.051	0.756
**40S ribosomal protein S3 OS=Mus musculus GN=Rps3 PE=1 SV=1**	0.991	1.065	1.028	1.124	1.095	1.274
**Nuclease-sensitive element-binding protein 1 OS=Mus musculus GN=Ybx1 PE=1 SV=3**	0.992	0.948	0.970	0.796	1.004	0.842
**Profilin-1 OS=Mus musculus GN=Pfn1 PE=1 SV=2**	1.016	1.033	1.024	1.233	1.165	1.110
**Ubiquitin-60S ribosomal protein L40 OS=Mus musculus GN=Uba52 PE=1 SV=2**	1.092	1.119	1.105	1.424	1.848	1.274
**Platelet-activating factor acetylhydrolase IB subunit alpha OS=Mus musculus GN=Pafah1b1 PE=1 SV=2**	1.032	1.158	1.095	1.248	1.559	1.196

**Heat shock cognate 71 kDa protein OS=Mus musculus GN=Hspa8 PE=1 SV=1**	1.026	1.046	1.036	1.623	1.786	1.529
**Translationally-controlled tumor protein OS=Mus musculus GN=Tpt1 PE=1 SV=1**	0.897	0.862	0.880	1.856	2.120	1.665
**DnaJ homolog subfamily A member 1 OS=Mus musculus GN=Dnaja1 PE=1 SV=1**	0.954	1.021	0.988	0.776	0.821	0.984
**60 kDa heat shock protein, mitochondrial OS=Mus musculus GN=Hspd1 PE=1 SV=1**	1.165	1.143	1.154	0.873	1.308	0.834
**Eukaryotic translation initiation factor 4E OS=Mus musculus GN=Eif4e PE=1 SV=1**	1.056	1.018	1.037	1.546	1.791	1.734
**14-3-3 protein zeta/delta OS=Mus musculus GN=Ywhaz PE=1 SV=1**	0.993	1.045	1.019	1.544	1.565	1.415
**High mobility group protein B1 OS=Mus musculus GN=Hmgb1 PE=1 SV=2**	0.909	0.877	0.893	2.586	3.213	2.427
**Dynein light chain 1, cytoplasmic OS=Mus musculus GN=Dynll1 PE=1 SV=1**	1.000	0.997	0.999	1.392	1.346	1.080
**Eukaryotic translation initiation factor 5A-1 OS=Mus musculus GN=Eif5a PE=1 SV=2**	1.044	1.018	1.031	1.822	1.872	1.534
**40S ribosomal protein S17 OS=Mus musculus GN=Rps17 PE=1 SV=2**	1.029	0.962	0.995	0.790	1.383	0.633
**SUMO-conjugating enzyme UBC9 OS=Mus musculus GN=Ube2i PE=1 SV=1**	0.990	1.000	0.995	1.562	1.806	1.678
**40S ribosomal protein S12 OS=Mus musculus GN=Rps12 PE=1 SV=2**	0.913	0.943	0.928	1.873	1.963	1.720
**40S ribosomal protein S10 OS=Mus musculus GN=Rps10 PE=1 SV=1**	0.969	0.881	0.925	0.829	1.137	0.727
**Serine/threonine-protein phosphatase 2B catalytic subunit alpha isoform OS=Mus musculus GN=Ppp3ca PE=1 SV=1**	1.000	0.921	0.960	0.855	0.877	1.448
**Serine/threonine-protein phosphatase 2A catalytic subunit alpha isoform OS=Mus musculus GN=Ppp2ca PE=1 SV=1**	1.049	0.949	0.999	0.944	0.895	0.938
**60S ribosomal protein L22 OS=Mus musculus GN=Rpl22 PE=1 SV=2**	0.969	1.019	0.994	1.136	1.229	1.265
**Actin, alpha cardiac muscle 1 OS=Mus musculus GN=Actc1 PE=1 SV=1**	0.902	0.995	0.948	1.148	1.130	1.104
**Ubiquitin-conjugating enzyme E2 L3 OS=Mus musculus GN=Ube2l3 PE=1 SV=1**	1.065	1.068	1.066	1.414	1.776	1.568
**Guanine nucleotide-binding protein subunit beta-2-like 1 OS=Mus musculus GN=Gnb2l1 PE=1 SV=3**	1.143	1.098	1.121	1.127	1.381	1.206
**14-3-3 protein theta OS=Mus musculus GN=Ywhaq PE=1 SV=1**	1.041	0.976	1.009	1.475	1.547	1.361
**Tubulin alpha-4A chain OS=Mus musculus GN=Tuba4a PE=1 SV=1**	1.000	1.005	1.003	5.666	2.975	4.718
**Tubulin beta-4B chain OS=Mus musculus GN=Tubb4b PE=1 SV=1**	0.967	0.993	0.980	1.194	1.170	1.173
**14-3-3 protein eta OS=Mus musculus GN=Ywhah PE=1 SV=2**	1.037	1.041	1.039	1.262	1.284	1.165
**Importin subunit beta-1 OS=Mus musculus GN=Kpnb1 PE=1 SV=2**	0.869	0.945	0.907	1.412	1.479	1.367
**Proteasome subunit beta type-7 OS=Mus musculus GN=Psmb7 PE=1 SV=1**	1.154	1.184	1.169	1.829	2.324	1.966
**Phosphatidylethanolamine-binding protein 1 OS=Mus musculus GN=Pebp1 PE=1 SV=3**	1.037	1.033	1.035	1.612	1.716	1.439
**Histidine triad nucleotide-binding protein 1 OS=Mus musculus GN=Hint1 PE=1 SV=3**	1.000	1.102	1.051	2.247	2.434	1.947
**Ubiquitin fusion degradation protein 1 homolog OS=Mus musculus GN=Ufd1l PE=1 SV=2**	0.987	0.967	0.977	1.037	0.929	1.114
**ELAV-like protein 1 OS=Mus musculus GN=Elavl1 PE=1 SV=2**	1.090	0.936	1.013	0.970	0.931	1.154
**Tumor suppressor p53-binding protein 1 OS=Mus**	1.000	1.314	1.157	1.508	1.149	1.718

musculus GN=Tp53bp1 PE=1 SV=2						
Exostosin-2 OS=Mus musculus GN=Ext2 PE=1 SV=2	1.013	1.056	1.034	1.076	0.955	0.944
Na(+)/H(+) exchange regulatory cofactor NHE-RF1 OS=Mus musculus GN=Slc9a3r1 PE=1 SV=3	1.128	1.028	1.078	1.166	1.208	1.227
Vasodilator-stimulated phosphoprotein OS=Mus musculus GN=Vasp PE=1 SV=4	1.000	0.853	0.926	1.024	0.989	1.016
Nascent polypeptide-associated complex subunit alpha, muscle-specific form OS=Mus musculus GN=Naca PE=1 SV=2	1.120	1.109	1.114	1.494	1.775	1.404
Caspase-3 OS=Mus musculus GN=Casp3 PE=1 SV=1	1.000	1.263	1.131	0.934	1.143	0.984
CTP synthase 1 OS=Mus musculus GN=Ctps1 PE=1 SV=2	1.000	0.879	0.940	0.920	0.781	0.878
Lysosomal alpha-glucosidase OS=Mus musculus GN=Gaa PE=1 SV=2	1.000	0.902	0.951	0.807	0.722	0.946
T-complex protein 1 subunit eta OS=Mus musculus GN=Cct7 PE=1 SV=1	1.020	1.102	1.061	1.455	1.602	1.297
T-complex protein 1 subunit beta OS=Mus musculus GN=Cct2 PE=1 SV=4	1.092	1.018	1.055	1.256	1.388	1.135
T-complex protein 1 subunit delta OS=Mus musculus GN=Cct4 PE=1 SV=3	1.057	1.136	1.096	1.207	1.244	1.331
T-complex protein 1 subunit epsilon OS=Mus musculus GN=Cct5 PE=1 SV=1	1.000	0.979	0.989	1.161	1.381	1.147
T-complex protein 1 subunit zeta OS=Mus musculus GN=Cct6a PE=1 SV=3	1.008	0.937	0.972	1.154	1.308	1.271
T-complex protein 1 subunit gamma OS=Mus musculus GN=Cct3 PE=1 SV=1	0.954	1.078	1.016	1.397	1.511	1.314
Nucleobindin-2 OS=Mus musculus GN=Nucb2 PE=1 SV=2	1.041	1.066	1.053	1.145	0.870	1.267
Transforming growth factor-beta-induced protein ig-h3 OS=Mus musculus GN=Tgfbi PE=1 SV=1	1.008	0.933	0.970	0.643	0.716	0.712
Stromal interaction molecule 2 OS=Mus musculus GN=Stim2 PE=1 SV=2	1.000	0.846	0.923	0.888	0.655	0.904
Chromobox protein homolog 1 OS=Mus musculus GN=Cbx1 PE=1 SV=1	1.000	1.161	1.080	1.007	1.481	1.303
Transcription elongation factor B polypeptide 1 OS=Mus musculus GN=Tceb1 PE=1 SV=1	1.032	1.010	1.021	1.133	1.210	1.090
Enhancer of rudimentary homolog OS=Mus musculus GN=Erh PE=1 SV=1	1.000	0.994	0.997	2.852	2.044	2.262
AP-2 complex subunit mu OS=Mus musculus GN=Ap2m1 PE=1 SV=1	1.108	1.081	1.095	1.077	1.191	1.233
Plasma protease C1 inhibitor OS=Mus musculus GN=Serping1 PE=1 SV=3	0.988	1.079	1.033	0.246	0.192	0.281
Pigment epithelium-derived factor OS=Mus musculus GN=Serpinf1 PE=1 SV=2	0.977	0.967	0.972	0.859	0.680	0.853
Secreted frizzled-related protein 2 OS=Mus musculus GN=Sfrp2 PE=2 SV=2	1.000	0.866	0.933	0.700	0.745	0.635
Neuroplastin OS=Mus musculus GN=Nptn PE=1 SV=3	1.000	0.675	0.837	0.487	0.444	0.347
DNA replication licensing factor MCM2 OS=Mus musculus GN=Mcm2 PE=1 SV=3	1.000	0.919	0.959	1.277	1.080	1.146
DNA replication licensing factor MCM6 OS=Mus musculus GN=Mcm6 PE=1 SV=1	0.975	0.921	0.948	1.014	1.327	1.225
Cysteine and glycine-rich protein 1 OS=Mus musculus GN=Csrp1 PE=1 SV=3	1.070	1.086	1.078	0.929	0.982	0.967
Nucleoredoxin OS=Mus musculus GN=Nxn PE=1 SV=1	0.034	0.517	0.276	33.651	24.646	25.586
40S ribosomal protein S3a OS=Mus musculus GN=Rps3a PE=1 SV=3	1.032	0.823	0.927	0.880	0.710	0.971
Proteasome activator complex subunit 1 OS=Mus musculus GN=Psme1 PE=1 SV=2	0.969	0.933	0.951	1.323	1.303	1.209
Ras GTPase-activating protein-binding protein 2	1.000	0.832	0.916	1.318	0.864	1.323

OS=Mus musculus GN=G3bp2 PE=1 SV=2						
Potassium voltage-gated channel subfamily KQT member 1 OS=Mus musculus GN=Kcnq1 PE=1 SV=3	1.000	0.621	0.811	0.202	0.207	0.298
Annexin A4 OS=Mus musculus GN=Anxa4 PE=1 SV=4	1.000	0.647	0.823	0.213	0.226	0.254
Myosin phosphatase Rho-interacting protein OS=Mus musculus GN=Mprip PE=1 SV=2	1.000	1.024	1.012	1.215	1.206	1.306
Peptidyl-glycine alpha-amidating monooxygenase OS=Mus musculus GN=Pam PE=1 SV=2	1.045	1.059	1.052	0.637	0.554	0.694
Glutamate--cysteine ligase catalytic subunit OS=Mus musculus GN=Gclc PE=1 SV=4	1.000	1.158	1.079	1.270	1.284	1.249
WW domain-binding protein 2 OS=Mus musculus GN=Wbp2 PE=1 SV=1	1.000	0.846	0.923	0.688	0.950	0.907
Neogenin OS=Mus musculus GN=Neo1 PE=1 SV=1	1.003	0.976	0.990	1.247	0.994	1.280
Fumarate hydratase, mitochondrial OS=Mus musculus GN=Fh PE=1 SV=3	1.075	1.086	1.081	0.946	1.070	1.031
Acidic leucine-rich nuclear phosphoprotein 32 family member E OS=Mus musculus GN=Anp32e PE=1 SV=2	1.000	0.859	0.930	1.452	1.660	1.319
Hematological and neurological expressed 1 protein OS=Mus musculus GN=Hn1 PE=1 SV=3	0.976	1.044	1.010	1.559	1.646	1.550
Ras GTPase-activating protein-binding protein 1 OS=Mus musculus GN=G3bp1 PE=1 SV=1	0.945	0.847	0.896	1.305	1.525	1.319
A disintegrin and metalloproteinase with thrombospondin motifs 1 OS=Mus musculus GN=Adamts1 PE=1 SV=4	1.000	0.908	0.954	0.282	0.163	0.287
Lysyl oxidase homolog 1 OS=Mus musculus GN=Loxl1 PE=2 SV=3	1.000	0.890	0.945	0.733	0.606	0.889
Laminin subunit alpha-4 OS=Mus musculus GN=Lama4 PE=1 SV=2	1.062	1.128	1.095	0.481	0.465	0.606
Vascular endothelial growth factor D OS=Mus musculus GN=Figf PE=2 SV=1	1.000	0.775	0.887	0.582	0.620	0.814
Bone morphogenetic protein 1 OS=Mus musculus GN=Bmp1 PE=1 SV=2	0.922	0.828	0.875	0.765	0.660	0.760
Mannan-binding lectin serine protease 1 OS=Mus musculus GN=Masp1 PE=1 SV=2	0.970	0.996	0.983	0.902	0.995	0.901
Very low-density lipoprotein receptor OS=Mus musculus GN=Vldlr PE=1 SV=1	1.000	0.869	0.935	0.708	0.415	0.410
Tubulin beta-5 chain OS=Mus musculus GN=Tubb5 PE=1 SV=1	1.000	1.044	1.022	1.302	1.686	1.147
Proteasome subunit beta type-4 OS=Mus musculus GN=Psmb4 PE=1 SV=1	1.015	1.048	1.032	1.466	1.478	1.585
60S acidic ribosomal protein P2 OS=Mus musculus GN=Rplp2 PE=1 SV=3	1.008	1.029	1.018	1.626	1.602	1.467
Peroxiredoxin-5, mitochondrial OS=Mus musculus GN=Prdx5 PE=1 SV=2	0.978	1.076	1.027	2.097	2.361	1.930
Carboxypeptidase E OS=Mus musculus GN=Cpe PE=1 SV=2	1.029	0.989	1.009	0.903	0.817	0.942
Xanthine dehydrogenase/oxidase OS=Mus musculus GN=Xdh PE=1 SV=5	1.180	1.139	1.160	1.549	1.346	1.513
Glucose-6-phosphate 1-dehydrogenase X OS=Mus musculus GN=G6pdx PE=1 SV=3	0.992	0.964	0.978	1.334	1.237	1.238
Apolipoprotein A-I OS=Mus musculus GN=Apoa1 PE=1 SV=2	1.000	0.989	0.994	0.964	1.010	1.518
Collagen alpha-1(VIII) chain OS=Mus musculus GN=Col8a1 PE=1 SV=3	1.000	0.988	0.994	0.503	0.455	0.558
Retinol-binding protein 1 OS=Mus musculus GN=Rbp1 PE=1 SV=2	1.000	0.962	0.981	0.353	0.360	0.349
Tyrosine-protein kinase receptor UFO OS=Mus musculus GN=Axl PE=1 SV=2	0.965	0.993	0.979	0.868	0.787	0.706

**Heterogeneous nuclear ribonucleoprotein U-like protein 2 OS=Mus musculus GN=Hnrnpul2 PE=1 SV=2**	1.000	0.876	0.938	1.081	1.253	1.421
**Collagen alpha-2(I) chain OS=Mus musculus GN=Col1a2 PE=1 SV=2**	1.111	1.032	1.071	0.992	0.860	0.865
**Epidermal growth factor receptor OS=Mus musculus GN=Egfr PE=1 SV=1**	1.000	1.034	1.017	0.989	1.062	1.462
**DNA topoisomerase 2-alpha OS=Mus musculus GN=Top2a PE=1 SV=2**	1.000	1.036	1.018	1.509	1.299	1.544
**Protein transport protein Sec23A OS=Mus musculus GN=Sec23a PE=1 SV=2**	0.925	1.066	0.995	1.271	1.167	1.199
**Guanylate-binding protein 1 OS=Mus musculus GN=Gbp1 PE=1 SV=1**	1.000	0.824	0.912	0.586	0.641	1.106
**Growth arrest-specific protein 1 OS=Mus musculus GN=Gas1 PE=2 SV=2**	0.998	1.080	1.039	1.246	1.545	1.511
**Ras suppressor protein 1 OS=Mus musculus GN=Rsu1 PE=1 SV=3**	1.000	1.086	1.043	1.154	1.329	1.183
**Nucleoside diphosphate kinase B OS=Mus musculus GN=Nme2 PE=1 SV=1**	0.709	0.782	0.745	1.452	1.947	1.455
**Transitional endoplasmic reticulum ATPase OS=Mus musculus GN=Vcp PE=1 SV=4**	1.047	1.147	1.097	0.671	0.770	0.649
**Ubiquitin-like modifier-activating enzyme 1 OS=Mus musculus GN=Uba1 PE=1 SV=1**	1.016	1.021	1.019	1.523	1.499	1.383
**Catenin beta-1 OS=Mus musculus GN=Ctnnb1 PE=1 SV=1**	1.000	0.995	0.997	0.963	0.846	0.952
**Myosin-6 OS=Mus musculus GN=Myh6 PE=1 SV=2**	1.000	1.096	1.048	0.280	0.353	0.527
**Collagen alpha-2(VI) chain OS=Mus musculus GN=Col6a2 PE=1 SV=3**	1.000	0.970	0.985	0.898	0.796	1.007
**Nucleobindin-1 OS=Mus musculus GN=Nucb1 PE=1 SV=2**	1.215	1.214	1.215	1.445	1.327	1.291
**ATP synthase subunit alpha, mitochondrial OS=Mus musculus GN=Atp5a1 PE=1 SV=1**	1.000	0.902	0.951	0.703	0.458	0.810
**Thrombospondin-2 OS=Mus musculus GN=Thbs2 PE=1 SV=2**	1.008	1.039	1.023	0.871	0.926	0.757
**C-C motif chemokine 7 OS=Mus musculus GN=Ccl7 PE=3 SV=1**	1.010	0.996	1.003	0.654	0.732	0.640
**Secretogranin-2 OS=Mus musculus GN=Scg2 PE=1 SV=1**	1.000	0.779	0.890	0.784	0.614	0.638
**Potassium voltage-gated channel subfamily B member 1 OS=Mus musculus GN=Kcnb1 PE=1 SV=2**	1.000	0.945	0.972	0.653	0.672	0.713
**Prefoldin subunit 6 OS=Mus musculus GN=Pfdn6 PE=1 SV=1**	1.058	1.015	1.037	1.019	1.293	1.143
**Creatine kinase B-type OS=Mus musculus GN=Ckb PE=1 SV=1**	0.878	0.932	0.905	0.450	0.465	0.663
**Sphingomyelin phosphodiesterase OS=Mus musculus GN=Smpd1 PE=2 SV=2**	1.000	0.896	0.948	0.662	0.390	0.689
**Collagen alpha-1(VI) chain OS=Mus musculus GN=Col6a1 PE=1 SV=1**	1.085	1.170	1.128	1.055	0.884	1.020
**Inhibin beta A chain OS=Mus musculus GN=Inhba PE=1 SV=1**	1.000	0.906	0.953	0.806	0.679	0.685
**Reticulocalbin-1 OS=Mus musculus GN=Rcn1 PE=1 SV=1**	0.995	0.846	0.920	0.663	0.679	0.626
**Basement membrane-specific heparan sulfate proteoglycan core protein OS=Mus musculus GN=Hspg2 PE=1 SV=1**	1.021	1.008	1.015	0.499	0.506	0.518
**Fatty acid-binding protein, epidermal OS=Mus musculus GN=Fabp5 PE=1 SV=3**	0.938	0.949	0.943	1.210	1.210	1.158
**Thrombospondin-3 OS=Mus musculus GN=Thbs3 PE=1 SV=2**	1.022	1.035	1.028	1.148	0.902	1.095
**La-related protein 7 OS=Mus musculus GN=Larp7**	1.000	1.924	1.462	1.527	1.100	1.700

**PE=1 SV=2**						
**Eukaryotic translation initiation factor 5B OS=Mus musculus GN=Eif5b PE=1 SV=2**	1.000	1.059	1.029	1.651	1.639	1.589
**Calcium-binding protein 39 OS=Mus musculus GN=Cab39 PE=1 SV=2**	1.000	0.915	0.958	1.585	1.751	1.431
**Amyloid-like protein 2 OS=Mus musculus GN=Aplp2 PE=1 SV=4**	1.000	1.081	1.041	0.599	0.510	0.737
**Clusterin OS=Mus musculus GN=Clu PE=1 SV=1**	1.081	1.123	1.102	0.417	0.307	0.379
**Glia-derived nexin OS=Mus musculus GN=Serpine2 PE=1 SV=2**	1.004	1.132	1.068	1.373	1.228	1.341
**Galectin-3-binding protein OS=Mus musculus GN=Lgals3bp PE=1 SV=1**	1.036	1.040	1.038	0.563	0.445	0.564
**Apoptosis regulator BAX OS=Mus musculus GN=Bax PE=1 SV=1**	1.000	1.145	1.072	1.182	0.995	1.044
**Core-binding factor subunit beta OS=Mus musculus GN=Cbfb PE=1 SV=1**	1.000	0.838	0.919	0.803	0.851	0.896
**Hepatocyte growth factor OS=Mus musculus GN=Hgf PE=1 SV=1**	1.000	1.055	1.028	0.889	0.914	0.848
**Calponin-1 OS=Mus musculus GN=Cnn1 PE=1 SV=1**	0.956	0.872	0.914	1.172	1.550	1.300
**Calponin-2 OS=Mus musculus GN=Cnn2 PE=1 SV=1**	0.976	1.109	1.043	1.241	1.391	1.562
**Cell growth-regulating nucleolar protein OS=Mus musculus GN=Lyar PE=1 SV=2**	0.984	1.511	1.248	1.935	1.702	2.342
**Vitamin K-dependent protein S OS=Mus musculus GN=Pros1 PE=2 SV=1**	1.000	0.768	0.884	0.639	0.603	0.543
**Fibulin-1 OS=Mus musculus GN=Fbln1 PE=1 SV=2**	0.953	0.948	0.950	0.883	0.775	0.762
**FACT complex subunit SSRP1 OS=Mus musculus GN=Ssrp1 PE=1 SV=2**	1.000	0.952	0.976	1.354	1.144	1.781
**Inverted formin-2 OS=Mus musculus GN=Inf2 PE=1 SV=1**	1.000	0.873	0.937	0.846	0.572	0.925
**Serine/threonine-protein phosphatase 4 regulatory subunit 2 OS=Mus musculus GN=Ppp4r2 PE=1 SV=1**	1.000	0.893	0.947	1.115	0.685	0.958
**Regulator of telomere elongation helicase 1 OS=Mus musculus GN=Rtel1 PE=1 SV=2**	1.000	0.784	0.892	0.996	0.668	0.851
**Puromycin-sensitive aminopeptidase OS=Mus musculus GN=Npepps PE=1 SV=2**	0.876	0.827	0.852	0.841	0.866	0.914
**Ubiquilin-like protein OS=Mus musculus GN=Ubqlnl PE=1 SV=2**	1.000	0.894	0.947	2.334	2.735	2.019
**tRNA (cytosine(34)-C(5))-methyltransferase OS=Mus musculus GN=Nsun2 PE=1 SV=2**	1.000	0.912	0.956	0.949	0.823	0.820
**Transmembrane channel-like protein 5 OS=Mus musculus GN=Tmc5 PE=2 SV=1**	1.000	0.979	0.990	0.984	0.700	0.949
**Putative phospholipase B-like 2 OS=Mus musculus GN=Plbd2 PE=1 SV=2**	1.019	1.002	1.010	0.849	0.865	0.868
**Myosin regulatory light chain 12B OS=Mus musculus GN=Myl12b PE=1 SV=2**	1.032	1.158	1.095	1.238	1.319	1.044
**General transcription factor IIF subunit 1 OS=Mus musculus GN=Gtf2f1 PE=1 SV=2**	1.000	0.773	0.887	1.259	1.209	1.017
**GMP synthase [glutamine-hydrolyzing] OS=Mus musculus GN=Gmps PE=1 SV=2**	1.000	0.814	0.907	0.840	0.923	0.883
**S-adenosylmethionine synthase isoform type-2 OS=Mus musculus GN=Mat2a PE=1 SV=2**	1.000	1.023	1.011	1.213	1.110	1.245
**PDZ and LIM domain protein 7 OS=Mus musculus GN=Pdlim7 PE=1 SV=1**	1.000	1.010	1.005	1.207	1.054	1.250
**Protein FAM98A OS=Mus musculus GN=Fam98a PE=1 SV=1**	1.000	1.192	1.096	1.314	1.618	0.795
**Transcription activator BRG1 OS=Mus musculus GN=Smarca4 PE=1 SV=1**	1.000	11.385	6.192	76.718	92.420	89.855
**Keratin, type II cytoskeletal 2 epidermal OS=Mus musculus GN=Krt2 PE=1 SV=1**	1.070	0.928	0.999	0.641	0.700	0.681
**UDP-N-acetylhexosamine pyrophosphorylase-like**	1.023	1.212	1.117	1.292	1.112	1.546

**protein 1 OS=Mus musculus GN=Uap1l1 PE=1 SV=1**						
**26S proteasome non-ATPase regulatory subunit 1 OS=Mus musculus GN=Psmd1 PE=1 SV=1**	0.963	1.075	1.019	1.215	1.170	1.316
**Far upstream element-binding protein 2 OS=Mus musculus GN=Khsrp PE=1 SV=2**	0.987	0.900	0.944	1.065	1.102	1.053
**DNA damage-binding protein 1 OS=Mus musculus GN=Ddb1 PE=1 SV=2**	1.000	0.979	0.990	1.112	1.304	1.139
**Valine--tRNA ligase, mitochondrial OS=Mus musculus GN=Vars2 PE=1 SV=2**	1.000	0.939	0.969	0.899	0.736	1.246
**Protein transport protein Sec24A OS=Mus musculus GN=Sec24a PE=1 SV=1**	1.000	0.861	0.930	0.613	0.620	1.234
**OTU domain-containing protein 5 OS=Mus musculus GN=Otud5 PE=1 SV=2**	1.000	0.928	0.964	0.727	0.816	1.055
**E3 ubiquitin-protein ligase HECTD3 OS=Mus musculus GN=Hectd3 PE=1 SV=2**	1.000	1.786	1.393	3.076	1.731	2.792
**Collagen alpha-2(V) chain OS=Mus musculus GN=Col5a2 PE=1 SV=1**	0.987	0.975	0.981	0.465	0.364	0.480
**Tubulin--tyrosine ligase-like protein 12 OS=Mus musculus GN=Ttll12 PE=1 SV=1**	1.000	1.177	1.088	1.010	1.196	1.118
**Ubiquitin-conjugating enzyme E2 Z OS=Mus musculus GN=Ube2z PE=1 SV=2**	1.000	1.045	1.022	1.312	1.094	1.378
**Poly(U)-binding-splicing factor PUF60 OS=Mus musculus GN=Puf60 PE=1 SV=2**	0.931	0.800	0.865	0.806	0.749	0.761
**RING finger and transmembrane domain-containing protein 2 OS=Mus musculus GN=Rnft2 PE=2 SV=2**	1.000	1.079	1.039	1.220	0.695	1.251
**Eukaryotic translation initiation factor 3 subunit J-A OS=Mus musculus GN=Eif3j1 PE=2 SV=1**	1.000	1.080	1.040	1.581	1.618	1.654
**AP2-associated protein kinase 1 OS=Mus musculus GN=Aak1 PE=1 SV=2**	1.000	0.829	0.914	1.654	1.089	0.932
**28 kDa heat- and acid-stable phosphoprotein OS=Mus musculus GN=Pdap1 PE=1 SV=1**	0.999	0.947	0.973	1.146	1.375	1.010
**BRISC and BRCA1-A complex member 1 OS=Mus musculus GN=Babam1 PE=1 SV=1**	1.000	0.906	0.953	0.881	0.862	0.632
**Rho GTPase-activating protein 17 OS=Mus musculus GN=Arhgap17 PE=1 SV=1**	1.000	0.840	0.920	0.832	0.932	0.416
**Enhancer of mRNA-decapping protein 4 OS=Mus musculus GN=Edc4 PE=1 SV=2**	1.000	1.177	1.089	0.963	1.013	1.170
**Protein C8orf37 homolog OS=Mus musculus PE=1 SV=1**	1.000	1.229	1.114	1.087	0.794	1.287
**Protein phosphatase 1 regulatory subunit 7 OS=Mus musculus GN=Ppp1r7 PE=1 SV=2**	1.000	1.075	1.037	0.892	0.893	1.565
**Protein PRRC1 OS=Mus musculus GN=Prrc1 PE=1 SV=1**	1.000	0.820	0.910	0.714	0.684	1.045
**Protein transport protein Sec31A OS=Mus musculus GN=Sec31a PE=1 SV=2**	1.018	0.996	1.007	1.054	1.165	1.249
**Peroxidasin homolog OS=Mus musculus GN=Pxdn PE=1 SV=2**	1.000	1.075	1.038	0.785	0.746	0.781
**Transforming growth factor-beta receptor-associated protein 1 OS=Mus musculus GN=Tgfbrap1 PE=1 SV=1**	1.000	1.179	1.090	2.458	2.441	2.121
**Keratin, type II cytoskeletal 2 oral OS=Mus musculus GN=Krt76 PE=1 SV=1**	1.000	0.776	0.888	0.237	0.231	0.347
**Leucine-rich repeat flightless-interacting protein 1 OS=Mus musculus GN=Lrrfip1 PE=1 SV=2**	1.000	1.094	1.047	0.878	0.760	0.985
**Protein sel-1 homolog 2 OS=Mus musculus GN=Sel1l2 PE=2 SV=1**	1.000	1.088	1.044	1.401	0.951	1.985
**Olfactomedin-like protein 2B OS=Mus musculus GN=Olfml2b PE=1 SV=2**	1.000	0.948	0.974	0.839	0.595	0.940
**Immunoglobulin superfamily member 10 OS=Mus**	1.000	0.948	0.974	0.943	0.874	1.063

**musculus GN=Igsf10 PE=2 SV=2**						
**Cytoplasmic dynein 2 heavy chain 1 OS=Mus musculus GN=Dync2h1 PE=1 SV=1**	1.000	1.227	1.114	1.039	1.060	0.962
**Costars family protein ABRACL OS=Mus musculus GN=Abracl PE=1 SV=1**	1.000	1.054	1.027	1.477	1.458	1.351
**1-phosphatidylinositol 4,5-bisphosphate phosphodiesterase eta-1 OS=Mus musculus GN=Plch1 PE=2 SV=1**	1.000	1.427	1.214	1.361	1.158	1.268
**Coiled-coil domain-containing protein 88B OS=Mus musculus GN=Ccdc88b PE=1 SV=2**	1.000	0.872	0.936	0.673	0.600	0.655
**Sister chromatid cohesion protein PDS5 homolog B OS=Mus musculus GN=Pds5b PE=1 SV=1**	1.000	0.861	0.931	0.789	0.554	0.867
**Protein CDV3 OS=Mus musculus GN=Cdv3 PE=1 SV=2**	0.937	0.888	0.912	1.079	0.914	0.939
**Probable ATP-dependent RNA helicase DDX17 OS=Mus musculus GN=Ddx17 PE=1 SV=1**	1.000	0.820	0.910	0.875	0.842	0.817
**Nuclear receptor subfamily 2 group C member 1 OS=Mus musculus GN=Nr2c1 PE=1 SV=3**	1.000	1.545	1.273	1.450	1.661	1.576
**Leucine-rich repeat-containing protein 47 OS=Mus musculus GN=Lrrc47 PE=1 SV=1**	1.000	1.114	1.057	0.899	0.966	1.376
**Thyroid hormone receptor-associated protein 3 OS=Mus musculus GN=Thrap3 PE=1 SV=1**	1.000	1.454	1.227	1.745	1.585	2.221
**N-acetylgalactosamine-6-sulfatase OS=Mus musculus GN=Galns PE=1 SV=2**	1.000	0.764	0.882	0.829	0.781	0.625
**Aldehyde dehydrogenase family 16 member A1 OS=Mus musculus GN=Aldh16a1 PE=1 SV=2**	1.000	0.837	0.918	0.695	0.450	0.687
**C-Jun-amino-terminal kinase-interacting protein 4 OS=Mus musculus GN=Spag9 PE=1 SV=2**	1.000	1.255	1.128	1.289	1.473	1.869
**Rho GTPase-activating protein 1 OS=Mus musculus GN=Arhgap1 PE=1 SV=1**	1.000	1.241	1.121	0.764	0.865	0.922
**Vacuolar protein sorting-associated protein 13A OS=Mus musculus GN=Vps13a PE=1 SV=1**	1.000	0.884	0.942	0.420	0.442	0.588
**Serine protease inhibitor A3G OS=Mus musculus GN=Serpina3g PE=1 SV=2**	1.000	0.882	0.941	0.814	0.854	1.034
**Mediator of DNA damage checkpoint protein 1 OS=Mus musculus GN=Mdc1 PE=1 SV=1**	1.000	0.925	0.963	1.046	0.695	0.824
**Phosphoribosylformylglycinamidine synthase OS=Mus musculus GN=Pfas PE=1 SV=1**	1.028	1.284	1.156	1.193	1.046	1.221
**Clustered mitochondria protein homolog OS=Mus musculus GN=Cluh PE=1 SV=2**	0.893	1.061	0.977	0.455	0.608	0.612
**Coatomer subunit delta OS=Mus musculus GN=Arcn1 PE=1 SV=2**	1.000	0.935	0.967	1.554	1.138	1.345
**Myosin light polypeptide 6 OS=Mus musculus GN=Myl6 PE=1 SV=3**	1.009	1.101	1.055	1.073	1.156	1.030
**Growth factor receptor-bound protein 2 OS=Mus musculus GN=Grb2 PE=1 SV=1**	0.974	0.992	0.983	1.100	1.110	0.995
**Ganglioside GM2 activator OS=Mus musculus GN=Gm2a PE=1 SV=2**	1.020	0.975	0.998	0.948	0.930	0.842
**Heterogeneous nuclear ribonucleoprotein D0 OS=Mus musculus GN=Hnrnpd PE=1 SV=2**	0.971	0.902	0.937	1.396	1.626	1.350
**Laminin subunit alpha-2 OS=Mus musculus GN=Lama2 PE=1 SV=2**	1.000	1.024	1.012	0.422	0.329	0.422
**Serine/threonine-protein phosphatase 5 OS=Mus musculus GN=Ppp5c PE=1 SV=3**	1.000	0.934	0.967	1.108	1.103	1.693
**Proteasome subunit beta type-6 OS=Mus musculus GN=Psmb6 PE=1 SV=3**	1.232	1.284	1.258	2.029	1.481	1.409
**Deoxynucleoside triphosphate triphosphohydrolase SAMHD1 OS=Mus musculus GN=Samhd1 PE=1 SV=2**	1.000	0.922	0.961	1.230	1.069	1.067
**Prolyl 4-hydroxylase subunit alpha-1 OS=Mus musculus GN=P4ha1 PE=1 SV=2**	1.000	0.979	0.989	0.591	0.998	0.646

**BAG family molecular chaperone regulator 1 OS=Mus musculus GN=Bag1 PE=1 SV=3**	1.000	0.884	0.942	0.900	0.893	0.755
**KH domain-containing, RNA-binding, signal transduction-associated protein 1 OS=Mus musculus GN=Khdrbs1 PE=1 SV=2**	1.000	1.338	1.169	1.498	1.689	1.637
**Growth arrest-specific protein 8 OS=Mus musculus GN=Gas8 PE=1 SV=2**	1.000	0.902	0.951	2.580	3.636	2.620
**Collagen alpha-1(XII) chain OS=Mus musculus GN=Col12a1 PE=2 SV=3**	0.965	0.980	0.973	0.549	0.602	0.591
**Serpin B6 OS=Mus musculus GN=Serpinb6 PE=1 SV=1**	0.921	0.944	0.932	1.139	1.058	1.041
**Stress-induced-phosphoprotein 1 OS=Mus musculus GN=Stip1 PE=1 SV=1**	0.941	0.898	0.920	1.449	1.817	1.453
**Caprin-1 OS=Mus musculus GN=Caprin1 PE=1 SV=2**	0.993	0.921	0.957	1.209	1.250	1.204
**Eukaryotic translation initiation factor 4E-binding protein 1 OS=Mus musculus GN=Eif4ebp1 PE=1 SV=3**	0.948	0.996	0.972	1.019	0.835	1.000
**Epidermal growth factor receptor substrate 15-like 1 OS=Mus musculus GN=Eps15l1 PE=1 SV=3**	1.000	1.281	1.141	1.927	1.971	1.940
**Centrosome-associated protein CEP250 OS=Mus musculus GN=Cep250 PE=1 SV=4**	1.000	0.958	0.979	0.761	0.850	1.032
**Histone-binding protein RBBP4 OS=Mus musculus GN=Rbbp4 PE=1 SV=5**	0.993	1.064	1.029	1.000	1.060	1.176
**Histone-binding protein RBBP7 OS=Mus musculus GN=Rbbp7 PE=1 SV=1**	1.000	0.968	0.984	1.373	1.629	1.496
**Laminin subunit alpha-5 OS=Mus musculus GN=Lama5 PE=1 SV=4**	1.000	1.042	1.021	0.507	0.437	0.644
**Asparagine synthetase [glutamine-hydrolyzing] OS=Mus musculus GN=Asns PE=1 SV=3**	0.773	0.988	0.880	1.240	1.597	1.356
**Lamina-associated polypeptide 2, isoforms beta/delta/epsilon/gamma OS=Mus musculus GN=Tmpo PE=1 SV=4**	1.029	1.108	1.069	1.597	1.388	1.672
**Histidine--tRNA ligase, cytoplasmic OS=Mus musculus GN=Hars PE=1 SV=2**	1.003	0.909	0.956	1.500	1.612	1.499
**Hsp90 co-chaperone Cdc37 OS=Mus musculus GN=Cdc37 PE=1 SV=1**	0.918	0.859	0.889	1.323	1.521	1.440
**45 kDa calcium-binding protein OS=Mus musculus GN=Sdf4 PE=1 SV=1**	1.000	0.983	0.992	0.747	0.831	0.967
**Ceruloplasmin OS=Mus musculus GN=Cp PE=1 SV=2**	1.014	1.052	1.033	1.355	1.075	1.392
**Microtubule-associated protein RP/EB family member 1 OS=Mus musculus GN=Mapre1 PE=1 SV=3**	1.000	1.099	1.049	0.925	1.092	0.903
**Peroxiredoxin-2 OS=Mus musculus GN=Prdx2 PE=1 SV=3**	0.982	0.978	0.980	1.587	1.683	1.569
**Methylosome subunit pICln OS=Mus musculus GN=Clns1a PE=1 SV=1**	1.000	1.153	1.076	1.316	0.871	1.306
**Host cell factor 1 OS=Mus musculus GN=Hcfc1 PE=1 SV=2**	1.000	0.965	0.983	1.354	1.374	1.714
**Platelet-activating factor acetylhydrolase IB subunit gamma OS=Mus musculus GN=Pafah1b3 PE=1 SV=1**	1.000	0.903	0.951	0.778	0.778	0.927
**Platelet-activating factor acetylhydrolase IB subunit beta OS=Mus musculus GN=Pafah1b2 PE=1 SV=2**	1.334	1.353	1.343	1.363	1.342	1.236
**Prosaposin OS=Mus musculus GN=Psap PE=1 SV=2**	1.081	1.079	1.080	0.957	0.946	0.880
**Beta-2-syntrophin OS=Mus musculus GN=Sntb2 PE=1 SV=2**	1.000	0.862	0.931	0.764	0.737	0.911
**Collagen alpha-1(XI) chain OS=Mus musculus GN=Col11a1 PE=1 SV=2**	1.005	1.036	1.020	0.521	0.406	0.670

**Laminin subunit beta-2 OS=Mus musculus GN=Lamb2 PE=1 SV=2**	1.044	0.945	0.995	0.486	0.394	0.540
**Catenin alpha-2 OS=Mus musculus GN=Ctnna2 PE=1 SV=3**	1.000	1.503	1.252	1.040	1.427	1.370
**Heat shock 70 kDa protein 4 OS=Mus musculus GN=Hspa4 PE=1 SV=1**	1.044	1.078	1.061	1.596	1.834	1.491
**B-cell receptor-associated protein 31 OS=Mus musculus GN=Bcap31 PE=1 SV=4**	1.000	1.110	1.055	1.005	0.995	1.055
**Procollagen C-endopeptidase enhancer 1 OS=Mus musculus GN=Pcolce PE=1 SV=2**	1.122	1.078	1.100	1.045	1.040	1.010
**Keratin, type I cytoskeletal 15 OS=Mus musculus GN=Krt15 PE=1 SV=2**	1.000	0.724	0.862	0.209	0.308	0.185
**H(+)/Cl(-) exchange transporter 4 OS=Mus musculus GN=Clcn4 PE=2 SV=2**	1.000	1.313	1.157	1.079	0.814	1.794
**Mesothelin OS=Mus musculus GN=Msln PE=1 SV=1**	0.928	0.864	0.896	1.374	1.339	1.456
**Delta-like protein 1 OS=Mus musculus GN=Dll1 PE=1 SV=2**	1.000	0.856	0.928	0.956	0.764	1.496
**Extracellular matrix protein 1 OS=Mus musculus GN=Ecm1 PE=1 SV=2**	1.024	0.983	1.004	0.722	0.680	0.649
**RNA-binding protein EWS OS=Mus musculus GN=Ewsr1 PE=1 SV=2**	1.053	1.185	1.119	1.804	1.805	1.691
**Double-strand-break repair protein rad21 homolog OS=Mus musculus GN=Rad21 PE=1 SV=3**	1.000	0.873	0.936	0.533	0.535	0.568
**Fascin OS=Mus musculus GN=Fscn1 PE=1 SV=4**	1.066	1.076	1.071	1.011	1.123	1.013
**Fibrillin-1 OS=Mus musculus GN=Fbn1 PE=1 SV=1**	1.038	1.057	1.047	0.375	0.353	0.410
**Peptidyl-prolyl cis-trans isomerase FKBP10 OS=Mus musculus GN=Fkbp10 PE=1 SV=2**	0.957	1.000	0.979	0.827	0.819	0.922
**Insulin-like growth factor-binding protein 7 OS=Mus musculus GN=Igfbp7 PE=1 SV=3**	1.029	1.040	1.035	0.598	0.586	0.589
**Kinectin OS=Mus musculus GN=Ktn1 PE=1 SV=1**	1.040	1.102	1.071	0.949	1.055	1.200
**Rab GDP dissociation inhibitor beta OS=Mus musculus GN=Gdi2 PE=1 SV=1**	1.033	0.994	1.014	1.265	1.406	1.293
**Rho GDP-dissociation inhibitor 2 OS=Mus musculus GN=Arhgdib PE=1 SV=3**	1.000	1.052	1.026	0.676	0.696	0.729
**Probable ATP-dependent RNA helicase DDX5 OS=Mus musculus GN=Ddx5 PE=1 SV=2**	1.000	0.953	0.977	1.244	1.526	1.281
**Chromobox protein homolog 5 OS=Mus musculus GN=Cbx5 PE=1 SV=1**	1.000	0.906	0.953	2.039	2.510	1.616
**Heat shock protein 105 kDa OS=Mus musculus GN=Hsph1 PE=1 SV=2**	0.905	0.880	0.892	0.979	1.034	0.972
**Inter-alpha-trypsin inhibitor heavy chain H1 OS=Mus musculus GN=Itih1 PE=1 SV=2**	1.000	1.292	1.146	1.659	1.413	3.075
**Inter-alpha-trypsin inhibitor heavy chain H2 OS=Mus musculus GN=Itih2 PE=1 SV=1**	1.067	1.050	1.059	1.038	0.798	1.020
**Inter-alpha-trypsin inhibitor heavy chain H3 OS=Mus musculus GN=Itih3 PE=1 SV=3**	0.995	1.014	1.004	0.950	0.799	1.228
**Interleukin-1 receptor accessory protein OS=Mus musculus GN=Il1rap PE=1 SV=1**	1.000	0.957	0.979	0.938	0.622	1.093
**D-3-phosphoglycerate dehydrogenase OS=Mus musculus GN=Phgdh PE=1 SV=3**	0.966	1.004	0.985	1.336	1.346	1.523
**Kinesin-1 heavy chain OS=Mus musculus GN=Kif5b PE=1 SV=3**	1.035	1.082	1.059	1.244	1.143	1.246
**Keratin, type I cytoskeletal 14 OS=Mus musculus GN=Krt14 PE=1 SV=2**	1.000	0.820	0.910	0.402	0.423	0.546
**LIM and SH3 domain protein 1 OS=Mus musculus GN=Lasp1 PE=1 SV=1**	1.101	1.014	1.057	0.937	0.840	0.843
**Lipopolysaccharide-binding protein OS=Mus musculus GN=Lbp PE=1 SV=2**	0.998	0.963	0.980	0.788	0.561	0.951
**Latent-transforming growth factor beta-binding protein 3 OS=Mus musculus GN=Ltbp3 PE=1 SV=3**	1.000	1.047	1.024	1.040	1.006	0.918
**Alpha-2-macroglobulin OS=Mus musculus**	1.000	1.049	1.025	1.121	1.044	1.258

GN=A2m PE=1 SV=3						
Myosin-10 OS=Mus musculus GN=Myh10 PE=1 SV=2	1.000	0.958	0.979	0.952	0.846	0.936
Nucleophosmin OS=Mus musculus GN=Npm1 PE=1 SV=1	1.028	1.034	1.031	1.407	1.625	1.290
Poly(rC)-binding protein 2 OS=Mus musculus GN=Pcbp2 PE=1 SV=1	0.891	0.781	0.836	1.150	1.222	1.020
Mimecan OS=Mus musculus GN=Ogn PE=1 SV=1	0.920	0.925	0.922	0.721	0.540	0.715
Periostin OS=Mus musculus GN=Postn PE=1 SV=2	1.063	1.237	1.150	0.228	0.184	0.271
Astrocytic phosphoprotein PEA-15 OS=Mus musculus GN=Pea15 PE=1 SV=1	1.000	0.930	0.965	0.883	0.859	0.760
Versican core protein OS=Mus musculus GN=Vcan PE=1 SV=2	1.072	1.081	1.077	0.884	0.868	0.950
Protein kinase C iota type OS=Mus musculus GN=Prkci PE=1 SV=3	1.000	0.693	0.846	0.964	1.253	0.895
Protein phosphatase 1 regulatory subunit 14B OS=Mus musculus GN=Ppp1r14b PE=1 SV=2	1.000	0.852	0.926	1.336	0.832	1.007
Serine/arginine-rich splicing factor 2 OS=Mus musculus GN=Srsf2 PE=1 SV=4	1.000	1.054	1.027	1.108	1.145	1.035
Dystroglycan OS=Mus musculus GN=Dag1 PE=1 SV=4	0.953	0.961	0.957	0.921	0.806	0.946
Dihydropyrimidinase-related protein 3 OS=Mus musculus GN=Dpysl3 PE=1 SV=1	1.010	1.022	1.016	1.335	1.161	1.451
U1 small nuclear ribonucleoprotein A OS=Mus musculus GN=Snrpa PE=1 SV=3	0.933	0.898	0.915	0.880	0.668	0.925
U1 small nuclear ribonucleoprotein C OS=Mus musculus GN=Snrpc PE=1 SV=1	1.000	0.901	0.951	1.008	0.808	1.352
Spectrin beta chain, non-erythrocytic 1 OS=Mus musculus GN=Sptbn1 PE=1 SV=2	0.954	1.013	0.983	0.788	0.811	0.764
Transcription intermediary factor 1-beta OS=Mus musculus GN=Trim28 PE=1 SV=3	1.003	1.004	1.003	1.028	1.087	1.010
Transferrin receptor protein 1 OS=Mus musculus GN=Tfrc PE=1 SV=1	1.000	0.849	0.924	0.472	0.540	0.974
Follistatin-related protein 1 OS=Mus musculus GN=Fstl1 PE=1 SV=2	0.973	1.033	1.003	0.509	0.572	0.527
Transcription elongation factor SPT6 OS=Mus musculus GN=Supt6h PE=1 SV=2	1.000	0.815	0.908	0.643	0.784	0.687
Zinc finger protein ZPR1 OS=Mus musculus GN=Zpr1 PE=1 SV=1	1.000	0.911	0.955	1.349	1.546	1.013
Tumor protein D52 OS=Mus musculus GN=Tpd52 PE=1 SV=2	1.002	1.007	1.004	1.093	1.101	1.056
Sorbin and SH3 domain-containing protein 1 OS=Mus musculus GN=Sorbs1 PE=1 SV=2	1.000	0.781	0.890	0.564	0.603	0.515
Drebrin-like protein OS=Mus musculus GN=Dbnl PE=1 SV=2	1.040	0.979	1.010	1.320	1.208	1.080
Endophilin-A2 OS=Mus musculus GN=Sh3gl1 PE=1 SV=1	1.000	0.869	0.935	0.706	0.603	0.592
Osteoclast-stimulating factor 1 OS=Mus musculus GN=Ostf1 PE=1 SV=2	1.000	1.043	1.021	1.091	0.738	1.136
Cystatin-B OS=Mus musculus GN=Cstb PE=1 SV=1	0.998	1.028	1.013	1.520	1.685	1.401
Protein NDRG1 OS=Mus musculus GN=Ndrg1 PE=1 SV=1	0.929	0.936	0.932	1.179	0.909	1.124
Peptidyl-prolyl cis-trans isomerase FKBP3 OS=Mus musculus GN=Fkbp3 PE=1 SV=2	1.120	1.081	1.100	1.332	1.625	1.180
Synaptic vesicle membrane protein VAT-1 homolog OS=Mus musculus GN=Vat1 PE=1 SV=3	1.029	1.067	1.048	1.085	1.074	1.219
Zyxin OS=Mus musculus GN=Zyx PE=1 SV=2	1.095	1.242	1.169	1.588	1.384	1.588
Calcineurin subunit B type 1 OS=Mus musculus GN=Ppp3r1 PE=1 SV=3	0.928	0.872	0.900	0.931	0.877	1.137
Mitogen-activated protein kinase 3 OS=Mus musculus GN=Mapk3 PE=1 SV=5	1.000	0.928	0.964	1.042	1.358	1.247

**Serum deprivation-response protein OS=Mus musculus GN=Sdpr PE=1 SV=3**	1.000	0.807	0.904	0.591	0.686	1.054
**Adapter molecule crk OS=Mus musculus GN=Crk PE=1 SV=1**	1.030	0.965	0.997	0.898	0.977	1.104
**RNA-binding protein Raly OS=Mus musculus GN=Raly PE=1 SV=3**	1.000	1.841	1.420	1.616	2.209	1.295
**Adipocyte enhancer-binding protein 1 OS=Mus musculus GN=Aebp1 PE=1 SV=1**	0.965	0.987	0.976	1.500	1.424	1.382
**Sepiapterin reductase OS=Mus musculus GN=Spr PE=1 SV=1**	0.940	0.917	0.928	1.226	1.075	1.320
**Transcription factor BTF3 OS=Mus musculus GN=Btf3 PE=1 SV=3**	1.000	1.008	1.004	0.814	0.814	0.931
**N(4)-(beta-N-acetylglucosaminyl)-L-asparaginase OS=Mus musculus GN=Aga PE=1 SV=1**	1.030	1.164	1.097	0.948	0.903	0.817
**Splicing factor 1 OS=Mus musculus GN=Sf1 PE=1 SV=6**	1.129	1.117	1.123	1.412	1.427	1.344
**Sequestosome-1 OS=Mus musculus GN=Sqstm1 PE=1 SV=1**	1.000	1.034	1.017	0.901	1.029	1.156
**Ubiquitin-like protein ISG15 OS=Mus musculus GN=Isg15 PE=1 SV=4**	1.032	0.958	0.995	1.188	0.957	1.017
**10 kDa heat shock protein, mitochondrial OS=Mus musculus GN=Hspe1 PE=1 SV=2**	1.127	1.136	1.131	0.695	1.232	0.750
**Sorbitol dehydrogenase OS=Mus musculus GN=Sord PE=1 SV=3**	0.920	0.732	0.826	0.704	0.691	0.644
**Carbonic anhydrase 4 OS=Mus musculus GN=Ca4 PE=1 SV=1**	1.000	0.908	0.954	0.897	0.351	1.205
**C-type mannose receptor 2 OS=Mus musculus GN=Mrc2 PE=1 SV=3**	0.997	0.988	0.992	0.702	0.520	0.483
**DNA topoisomerase 2-beta OS=Mus musculus GN=Top2b PE=1 SV=2**	1.000	0.812	0.906	1.343	1.329	1.038
**Tripeptidyl-peptidase 2 OS=Mus musculus GN=Tpp2 PE=1 SV=3**	1.048	1.044	1.046	1.322	1.185	1.188
**NAD(P)H dehydrogenase [quinone] 1 OS=Mus musculus GN=Nqo1 PE=1 SV=3**	1.000	0.926	0.963	1.213	1.448	1.344
**Spermidine synthase OS=Mus musculus GN=Srm PE=1 SV=1**	1.000	0.923	0.961	1.317	1.248	1.072
**Endothelial protein C receptor OS=Mus musculus GN=Procr PE=1 SV=3**	1.000	1.004	1.002	0.718	0.620	0.973
**Vinculin OS=Mus musculus GN=Vcl PE=1 SV=4**	1.083	1.182	1.132	1.448	1.415	1.403
**Trifunctional purine biosynthetic protein adenosine-3 OS=Mus musculus GN=Gart PE=1 SV=3**	1.000	1.018	1.009	1.380	1.457	1.392
**Clathrin heavy chain 1 OS=Mus musculus GN=Cltc PE=1 SV=3**	0.985	1.039	1.012	0.917	1.102	0.914
**Methionine--tRNA ligase, cytoplasmic OS=Mus musculus GN=Mars PE=1 SV=1**	1.000	1.080	1.040	1.569	1.152	1.237
**Dynein heavy chain 17, axonemal OS=Mus musculus GN=Dnah17 PE=1 SV=2**	1.000	0.695	0.847	1.094	0.590	0.765
**Myoferlin OS=Mus musculus GN=Myof PE=1 SV=2**	0.908	0.875	0.891	1.277	1.239	1.356
**Sister chromatid cohesion protein PDS5 homolog A OS=Mus musculus GN=Pds5a PE=1 SV=3**	1.000	0.719	0.860	0.654	0.888	0.447
**Switch-associated protein 70 OS=Mus musculus GN=Swap70 PE=1 SV=2**	1.000	0.899	0.950	0.898	0.815	0.743
**Cell division cycle 5-like protein OS=Mus musculus GN=Cdc5l PE=1 SV=2**	1.000	0.986	0.993	0.884	0.929	0.911
**Cingulin-like protein 1 OS=Mus musculus GN=Cgnl1 PE=1 SV=2**	1.000	0.723	0.861	0.394	0.889	0.680
**Nucleolar protein 58 OS=Mus musculus GN=Nop58 PE=1 SV=1**	1.000	1.012	1.006	0.355	0.563	0.695
**Alpha-2-macroglobulin-P OS=Mus musculus GN=A2mp PE=2 SV=2**	0.993	1.030	1.012	1.183	1.087	1.344

**Keratin, type I cytoskeletal 42 OS=Mus musculus GN=Krt42 PE=1 SV=1**	0.911	0.839	0.875	0.413	0.540	0.369
**Tropomyosin alpha-4 chain OS=Mus musculus GN=Tpm4 PE=1 SV=3**	1.007	1.045	1.026	1.260	1.345	1.173
**Undifferentiated embryonic cell transcription factor 1 OS=Mus musculus GN=Utf1 PE=1 SV=2**	1.000	0.818	0.909	1.656	0.777	0.980
**Cleavage and polyadenylation specificity factor subunit 6 OS=Mus musculus GN=Cpsf6 PE=1 SV=1**	1.000	0.776	0.888	1.059	0.805	0.851
**Keratin, type II cytoskeletal 73 OS=Mus musculus GN=Krt73 PE=1 SV=1**	0.976	0.787	0.882	0.292	0.378	0.243
**DnaJ homolog subfamily C member 8 OS=Mus musculus GN=Dnajc8 PE=1 SV=2**	1.000	1.092	1.046	1.653	1.195	1.049
**Eukaryotic translation initiation factor 4 gamma 1 OS=Mus musculus GN=Eif4g1 PE=1 SV=1**	1.115	1.015	1.065	1.194	1.143	1.256
**RNA-binding protein 26 OS=Mus musculus GN=Rbm26 PE=1 SV=2**	1.000	0.870	0.935	0.889	0.945	1.220
**Xaa-Pro aminopeptidase 1 OS=Mus musculus GN=Xpnpep1 PE=1 SV=1**	1.099	1.231	1.165	1.246	1.415	1.382
**Serine/threonine-protein phosphatase 2A 55 kDa regulatory subunit B alpha isoform OS=Mus musculus GN=Ppp2r2a PE=1 SV=1**	1.000	1.117	1.059	1.378	1.176	1.321
**Serine/threonine-protein phosphatase 4 regulatory subunit 3A OS=Mus musculus GN=Ppp4r3a PE=1 SV=1**	1.000	0.631	0.815	0.322	0.246	0.664
**U5 small nuclear ribonucleoprotein 200 kDa helicase OS=Mus musculus GN=Snrnp200 PE=1 SV=1**	1.000	1.010	1.005	1.065	0.892	1.106
**ATP-binding cassette sub-family F member 1 OS=Mus musculus GN=Abcf1 PE=1 SV=1**	1.000	0.976	0.988	0.975	0.631	1.438
**Phosphatidylinositol 3,4,5-trisphosphate 5-phosphatase 2 OS=Mus musculus GN=Inppl1 PE=1 SV=1**	1.000	0.897	0.948	0.644	0.764	1.296
**UDP-glucose:glycoprotein glucosyltransferase 1 OS=Mus musculus GN=Uggt1 PE=1 SV=4**	1.004	0.902	0.953	0.943	0.579	0.690
**Exportin-1 OS=Mus musculus GN=Xpo1 PE=1 SV=1**	1.059	0.962	1.010	1.154	1.264	1.127
**Nestin OS=Mus musculus GN=Nes PE=1 SV=1**	1.000	1.029	1.015	0.769	0.768	0.947
**PEST proteolytic signal-containing nuclear protein OS=Mus musculus GN=Pcnp PE=1 SV=1**	1.000	0.906	0.953	1.397	1.644	1.450
**Sorting nexin-6 OS=Mus musculus GN=Snx6 PE=1 SV=2**	1.000	0.831	0.915	1.030	0.928	1.157
**Kinesin-like protein KIF11 OS=Mus musculus GN=Kif11 PE=1 SV=1**	1.000	0.948	0.974	0.864	0.976	0.887
**FK506-binding protein 15 OS=Mus musculus GN=Fkbp15 PE=1 SV=2**	1.000	0.921	0.961	0.687	0.480	0.624
**Serine/threonine-protein kinase OSR1 OS=Mus musculus GN=Oxsr1 PE=1 SV=1**	1.000	0.991	0.996	0.785	1.013	1.016
**AT-hook DNA-binding motif-containing protein 1 OS=Mus musculus GN=Ahdc1 PE=1 SV=1**	1.000	0.897	0.948	1.353	0.902	0.819
**Tyrosine-protein phosphatase non-receptor type 23 OS=Mus musculus GN=Ptpn23 PE=1 SV=2**	1.000	0.939	0.969	0.765	0.770	1.192
**Leucine-rich PPR motif-containing protein, mitochondrial OS=Mus musculus GN=Lrpprc PE=1 SV=2**	1.000	0.838	0.919	0.706	0.598	0.726
**Polypeptide N-acetylgalactosaminyltransferase 2 OS=Mus musculus GN=Galnt2 PE=1 SV=1**	1.000	0.742	0.871	0.476	0.440	0.501
**SWI/SNF complex subunit SMARCC2 OS=Mus musculus GN=Smarcc2 PE=1 SV=2**	1.000	0.940	0.970	0.859	1.072	1.036
**Histone-lysine N-methyltransferase 2D OS=Mus musculus GN=Kmt2d PE=1 SV=2**	1.000	1.457	1.228	2.049	1.796	1.064
**Serine/arginine-rich splicing factor 1 OS=Mus musculus GN=Srsf1 PE=1 SV=3**	0.985	0.974	0.979	1.082	1.129	1.036

**Platelet-derived growth factor receptor-like protein OS=Mus musculus GN=Pdgfrl PE=2 SV=1**	1.095	0.979	1.037	1.332	1.269	1.278
**UPF0704 protein C6orf165 homolog OS=Mus musculus PE=2 SV=2**	1.000	0.721	0.861	0.219	0.282	0.201
**Tankyrase-1 OS=Mus musculus GN=Tnks PE=1 SV=1**	1.000	1.030	1.015	1.254	1.638	1.360
**N-alpha-acetyltransferase 50 OS=Mus musculus GN=Naa50 PE=1 SV=1**	1.000	1.042	1.021	1.490	1.388	1.028
**Hematological and neurological expressed 1-like protein OS=Mus musculus GN=Hn1l PE=1 SV=1**	1.000	1.188	1.094	1.952	2.715	2.763
**WASH complex subunit FAM21 OS=Mus musculus GN=Fam21 PE=1 SV=1**	1.000	0.847	0.924	1.115	0.823	1.295
**NudC domain-containing protein 1 OS=Mus musculus GN=Nudcd1 PE=1 SV=2**	1.000	1.001	1.001	1.288	1.082	1.597
**Guanine nucleotide-binding protein G(s) subunit alpha isoforms XLas OS=Mus musculus GN=Gnas PE=1 SV=1**	1.000	0.900	0.950	0.778	0.827	0.700
**N-acetylglucosamine-1-phosphotransferase subunit gamma OS=Mus musculus GN=Gnptg PE=1 SV=1**	1.000	0.971	0.985	0.826	0.540	0.910
**Protein Daple OS=Mus musculus GN=Ccdc88c PE=1 SV=1**	0.019	26.351	13.185	28.499	25.090	51.909
**Centrosomal protein of 162 kDa OS=Mus musculus GN=Cep162 PE=1 SV=2**	0.970	0.961	0.965	1.378	0.921	1.538
**Cullin-associated NEDD8-dissociated protein 1 OS=Mus musculus GN=Cand1 PE=1 SV=2**	1.129	1.050	1.089	1.346	1.473	1.303
**U6 snRNA-associated Sm-like protein LSm8 OS=Mus musculus GN=Lsm8 PE=1 SV=3**	1.071	0.901	0.986	1.021	1.347	1.304
**40S ribosomal protein S27 OS=Mus musculus GN=Rps27 PE=1 SV=3**	1.000	0.876	0.938	1.539	1.255	1.345
**60S ribosomal protein L10 OS=Mus musculus GN=Rpl10 PE=1 SV=3**	1.000	1.760	1.380	0.963	1.685	1.861
**Eukaryotic translation initiation factor 2 subunit 1 OS=Mus musculus GN=Eif2s1 PE=1 SV=3**	1.023	1.021	1.022	1.311	1.490	1.403
**Ubiquitin-conjugating enzyme E2 R2 OS=Mus musculus GN=Ube2r2 PE=1 SV=1**	1.000	1.271	1.136	4.924	4.393	6.968
**Serine/threonine-protein phosphatase 2A 65 kDa regulatory subunit A alpha isoform OS=Mus musculus GN=Ppp2r1a PE=1 SV=3**	0.859	0.908	0.884	1.436	1.605	1.283
**Multivesicular body subunit 12A OS=Mus musculus GN=Mvb12a PE=1 SV=1**	1.000	1.343	1.172	1.302	1.162	1.114
**Ubiquitin domain-containing protein UBFD1 OS=Mus musculus GN=Ubfd1 PE=1 SV=2**	1.000	0.984	0.992	1.238	1.474	1.656
**Staphylococcal nuclease domain-containing protein 1 OS=Mus musculus GN=Snd1 PE=1 SV=1**	1.016	0.961	0.989	1.066	1.124	1.132
**Nucleosome assembly protein 1-like 4 OS=Mus musculus GN=Nap1l4 PE=1 SV=1**	0.962	0.935	0.949	1.537	1.722	1.272
**Phosphatidylinositol-binding clathrin assembly protein OS=Mus musculus GN=Picalm PE=1 SV=1**	1.000	0.868	0.934	0.699	0.582	0.767
**Heterogeneous nuclear ribonucleoprotein Q OS=Mus musculus GN=Syncrip PE=1 SV=2**	1.084	1.013	1.049	1.270	1.368	1.270
**Tubulin beta-2A chain OS=Mus musculus GN=Tubb2a PE=1 SV=1**	1.000	1.101	1.051	0.701	0.774	0.832
**Lysosomal Pro-X carboxypeptidase OS=Mus musculus GN=Prcp PE=1 SV=2**	1.045	1.280	1.162	1.971	1.277	1.991
**E3 ubiquitin-protein ligase HUWE1 OS=Mus musculus GN=Huwe1 PE=1 SV=5**	1.000	1.017	1.009	0.743	0.710	1.011
**Putative RNA-binding protein Luc7-like 2 OS=Mus musculus GN=Luc7l2 PE=1 SV=1**	1.000	0.846	0.923	0.573	0.559	0.556
**Protein DEK OS=Mus musculus GN=Dek PE=1 SV=1**	1.000	0.878	0.939	1.542	1.204	1.335

**Integrator complex subunit 3 OS=Mus musculus GN=Ints3 PE=1 SV=2**	1.000	0.843	0.921	1.095	0.831	0.791
**Protein PRRC2B OS=Mus musculus GN=Prrc2b PE=1 SV=1**	1.000	0.866	0.933	0.701	1.339	1.650
**Alpha-actinin-1 OS=Mus musculus GN=Actn1 PE=1 SV=1**	1.054	1.174	1.114	1.466	1.644	1.428
**Ubiquitin thioesterase OTUB1 OS=Mus musculus GN=Otub1 PE=1 SV=2**	0.997	0.923	0.960	1.168	1.493	1.343
**Leucine-rich repeat-containing protein 75A OS=Mus musculus GN=Lrrc75a PE=1 SV=1**	1.000	1.014	1.007	0.726	0.831	0.605
**ADAMTS-like protein 2 OS=Mus musculus GN=Adamtsl2 PE=2 SV=1**	1.000	1.096	1.048	0.970	0.604	1.053
**Phosphoglucomutase-2 OS=Mus musculus GN=Pgm2 PE=1 SV=1**	1.000	1.721	1.361	1.125	1.074	1.162
**Interferon-induced very large GTPase 1 OS=Mus musculus GN=Gvin1 PE=1 SV=1**	1.000	0.981	0.991	0.576	0.485	0.812
**Protein LAP2 OS=Mus musculus GN=Erbb2ip PE=1 SV=3**	1.000	0.784	0.892	0.830	0.574	0.682
**Histone lysine demethylase PHF8 OS=Mus musculus GN=Phf8 PE=1 SV=2**	1.000	1.160	1.080	1.378	1.263	2.423
**Nischarin OS=Mus musculus GN=Nisch PE=1 SV=2**	1.000	0.947	0.973	1.471	1.124	1.213
**Vacuolar protein sorting-associated protein 13B OS=Mus musculus GN=Vps13b PE=1 SV=2**	1.000	0.791	0.896	1.784	0.745	1.068
**Septin-9 OS=Mus musculus GN=Sept9 PE=1 SV=1**	1.000	0.806	0.903	0.644	0.774	1.338
**N-alpha-acetyltransferase 15, NatA auxiliary subunit OS=Mus musculus GN=Naa15 PE=1 SV=1**	1.000	0.851	0.926	1.246	1.097	1.792
**DNA-directed RNA polymerases I, II, and III subunit RPABC1 OS=Mus musculus GN=Polr2e PE=1 SV=1**	1.000	0.816	0.908	0.798	1.236	1.141
**Steroid receptor RNA activator 1 OS=Mus musculus GN=Sra1 PE=1 SV=3**	1.000	1.128	1.064	1.511	1.072	1.309
**2'-deoxynucleoside 5'-phosphate N-hydrolase 1 OS=Mus musculus GN=Dnph1 PE=1 SV=2**	1.000	2.097	1.549	2.762	1.210	1.748
**Transmembrane protein 202 OS=Mus musculus GN=Tmem202 PE=2 SV=1**	1.000	1.127	1.064	0.978	0.878	1.479
**Protein LYRIC OS=Mus musculus GN=Mtdh PE=1 SV=1**	1.000	0.882	0.941	1.023	0.920	0.893
**Collagen alpha-1(XIV) chain OS=Mus musculus GN=Col14a1 PE=1 SV=2**	1.000	0.839	0.920	0.269	0.172	0.319
**Serine/threonine-protein kinase VRK1 OS=Mus musculus GN=Vrk1 PE=1 SV=2**	1.000	1.144	1.072	0.816	0.964	0.741
**Ubiquitin-associated protein 2-like OS=Mus musculus GN=Ubap2l PE=1 SV=1**	1.006	0.956	0.981	1.326	1.537	1.354
**Filamin-B OS=Mus musculus GN=Flnb PE=1 SV=3**	0.963	1.052	1.007	1.348	1.402	1.373
**Nuclear ubiquitous casein and cyclin-dependent kinase substrate 1 OS=Mus musculus GN=Nucks1 PE=1 SV=1**	1.000	1.098	1.049	1.589	1.436	1.466
**Zinc phosphodiesterase ELAC protein 2 OS=Mus musculus GN=Elac2 PE=1 SV=1**	1.000	0.907	0.953	1.077	0.949	0.793
**Tenascin OS=Mus musculus GN=Tnc PE=1 SV=1**	1.000	1.059	1.029	0.974	1.220	0.959
**E3 ubiquitin-protein ligase LRSAM1 OS=Mus musculus GN=Lrsam1 PE=1 SV=1**	1.000	1.052	1.026	0.710	0.613	1.272
**Protein Simiate OS=Mus musculus GN=Fam206a PE=1 SV=2**	1.000	1.306	1.153	1.659	1.342	1.364
**ATP-dependent RNA helicase DDX42 OS=Mus musculus GN=Ddx42 PE=1 SV=3**	1.000	0.913	0.956	0.671	0.817	0.987
**Glutamate-rich WD repeat-containing protein 1 OS=Mus musculus GN=Grwd1 PE=1 SV=2**	1.000	0.858	0.929	0.575	0.778	0.893
**Neuronal cell adhesion molecule OS=Mus musculus GN=Nrcam PE=1 SV=2**	1.000	0.876	0.938	0.625	0.988	0.602
**Cysteine protease ATG4C OS=Mus musculus**	1.000	1.081	1.041	1.529	1.055	1.383

**GN=Atg4c PE=1 SV=2**						
**Disks large homolog 1 OS=Mus musculus GN=Dlg1 PE=1 SV=1**	1.000	1.142	1.071	1.068	1.246	1.592
**WD repeat-containing protein 82 OS=Mus musculus GN=Wdr82 PE=1 SV=1**	1.030	0.872	0.951	1.327	1.556	1.124
**N-acetylglucosamine-6-sulfatase OS=Mus musculus GN=Gns PE=1 SV=1**	0.960	0.943	0.951	0.552	0.468	0.585
**Elongation factor Tu, mitochondrial OS=Mus musculus GN=Tufm PE=1 SV=1**	1.000	0.822	0.911	0.245	0.226	0.381
**Lipoma-preferred partner homolog OS=Mus musculus GN=Lpp PE=1 SV=1**	1.000	1.103	1.051	1.236	1.313	1.269
**Transportin-1 OS=Mus musculus GN=Tnpo1 PE=1 SV=2**	0.994	0.921	0.957	1.067	1.087	0.990
**Beta-actin-like protein 2 OS=Mus musculus GN=Actbl2 PE=1 SV=1**	1.000	1.093	1.046	1.196	1.206	1.861
**Heterogeneous nuclear ribonucleoprotein A3 OS=Mus musculus GN=Hnrnpa3 PE=1 SV=1**	1.079	1.130	1.104	1.754	2.104	1.534
**Calcium-activated chloride channel regulator 2 OS=Mus musculus GN=Clca2 PE=2 SV=1**	1.000	0.917	0.958	0.785	0.595	1.217
**26S proteasome non-ATPase regulatory subunit 11 OS=Mus musculus GN=Psmd11 PE=1 SV=3**	1.038	0.991	1.015	1.035	1.216	1.097
**Eukaryotic translation initiation factor 4B OS=Mus musculus GN=Eif4b PE=1 SV=1**	0.895	1.142	1.019	1.627	1.729	1.623
**WD repeat-containing protein 92 OS=Mus musculus GN=Wdr92 PE=1 SV=1**	1.000	0.786	0.893	1.014	0.614	0.866
**Alanine--tRNA ligase, cytoplasmic OS=Mus musculus GN=Aars PE=1 SV=1**	1.037	1.054	1.045	1.870	1.663	1.793
**Alpha-ketoglutarate-dependent dioxygenase FTO OS=Mus musculus GN=Fto PE=1 SV=1**	1.000	0.838	0.919	0.537	0.738	0.931
**Keratin, type II cytoskeletal 75 OS=Mus musculus GN=Krt75 PE=1 SV=1**	1.000	0.880	0.940	0.409	0.685	0.634
**TIP41-like protein OS=Mus musculus GN=Tiprl PE=1 SV=1**	1.000	1.073	1.037	0.633	0.963	0.700
**Selenide, water dikinase 1 OS=Mus musculus GN=Sephs1 PE=1 SV=1**	0.791	1.119	0.955	1.637	1.627	1.489
**Reticulocalbin-3 OS=Mus musculus GN=Rcn3 PE=1 SV=1**	1.015	0.971	0.993	0.891	0.996	1.032
**Probable D-tyrosyl-tRNA(Tyr) deacylase 2 OS=Mus musculus GN=Dtd2 PE=1 SV=1**	1.000	1.066	1.033	0.874	0.481	0.743
**Nardilysin OS=Mus musculus GN=Nrd1 PE=1 SV=1**	1.000	0.824	0.912	1.309	1.200	1.116
**Engulfment and cell motility protein 2 OS=Mus musculus GN=Elmo2 PE=1 SV=1**	1.000	1.128	1.064	1.022	0.820	1.182
**Neutral alpha-glucosidase AB OS=Mus musculus GN=Ganab PE=1 SV=1**	1.000	0.900	0.950	0.805	0.919	0.826
**Cleavage stimulation factor subunit 2 OS=Mus musculus GN=Cstf2 PE=1 SV=2**	1.000	0.881	0.940	1.030	0.992	0.929
**Nuclear pore complex protein Nup93 OS=Mus musculus GN=Nup93 PE=1 SV=1**	0.971	1.014	0.993	0.746	1.071	0.998
**Small glutamine-rich tetratricopeptide repeat-containing protein alpha OS=Mus musculus GN=Sgta PE=1 SV=2**	1.000	0.918	0.959	1.094	1.016	1.178
**Eukaryotic translation initiation factor 2A OS=Mus musculus GN=Eif2a PE=1 SV=2**	0.969	0.996	0.982	1.185	1.132	1.291
**26S proteasome non-ATPase regulatory subunit 5 OS=Mus musculus GN=Psmd5 PE=1 SV=4**	0.942	0.992	0.967	1.264	1.244	1.361
**Olfactomedin-like protein 3 OS=Mus musculus GN=Olfml3 PE=2 SV=2**	1.058	0.986	1.022	0.808	0.804	0.832
**Activator of 90 kDa heat shock protein ATPase homolog 1 OS=Mus musculus GN=Ahsa1 PE=1 SV=2**	1.130	1.238	1.184	1.712	1.765	2.021
**Protein RCC2 OS=Mus musculus GN=Rcc2 PE=1**	1.032	1.048	1.040	0.937	1.107	1.040

**SV=1**						
**Importin-5 OS=Mus musculus GN=Ipo5 PE=1 SV=3**	0.959	0.930	0.945	1.483	1.900	1.457
**Inactive tyrosine-protein kinase 7 OS=Mus musculus GN=Ptk7 PE=1 SV=1**	1.000	1.090	1.045	0.999	0.961	1.186
**Serine/threonine-protein kinase SMG1 OS=Mus musculus GN=Smg1 PE=1 SV=3**	1.000	1.012	1.006	0.870	0.837	0.815
**Early endosome antigen 1 OS=Mus musculus GN=Eea1 PE=1 SV=2**	0.971	0.968	0.969	1.039	1.113	1.276
**Serine/arginine-rich splicing factor 7 OS=Mus musculus GN=Srsf7 PE=1 SV=1**	1.000	0.824	0.912	0.532	0.597	0.525
**Sodium/hydrogen exchanger 7 OS=Mus musculus GN=Slc9a7 PE=1 SV=1**	1.000	1.174	1.087	1.734	0.864	0.977
**Heat shock 70 kDa protein 13 OS=Mus musculus GN=Hspa13 PE=1 SV=1**	1.004	1.099	1.051	0.960	0.851	0.974
**Leucine--tRNA ligase, cytoplasmic OS=Mus musculus GN=Lars PE=1 SV=2**	0.979	1.027	1.003	1.090	0.943	1.164
**Eukaryotic translation initiation factor 1A, X-chromosomal OS=Mus musculus GN=Eif1ax PE=2 SV=3**	1.014	0.953	0.984	1.268	1.740	1.344
**Cytoskeleton-associated protein 4 OS=Mus musculus GN=Ckap4 PE=1 SV=2**	1.055	1.079	1.067	0.701	0.576	0.670
**Spondin-2 OS=Mus musculus GN=Spon2 PE=2 SV=2**	1.034	1.261	1.148	0.335	0.289	0.336
**Sulfhydryl oxidase 1 OS=Mus musculus GN=Qsox1 PE=1 SV=1**	0.978	1.015	0.996	1.034	0.918	1.075
**Asparagine--tRNA ligase, cytoplasmic OS=Mus musculus GN=Nars PE=1 SV=2**	1.000	0.993	0.996	1.548	1.418	1.312
**Methionine aminopeptidase 1 OS=Mus musculus GN=Metap1 PE=1 SV=1**	1.000	0.674	0.837	0.300	0.388	0.319
**Reticulocalbin-2 OS=Mus musculus GN=Rcn2 PE=1 SV=1**	1.126	1.014	1.070	1.110	1.158	1.088
**MOB kinase activator 1B OS=Mus musculus GN=Mob1b PE=1 SV=3**	1.000	0.730	0.865	0.879	0.848	1.440
**EGF-containing fibulin-like extracellular matrix protein 1 OS=Mus musculus GN=Efemp1 PE=1 SV=1**	1.000	1.028	1.014	0.566	0.293	0.451
**LIX1-like protein OS=Mus musculus GN=Lix1l PE=2 SV=2**	1.000	0.957	0.979	1.073	0.691	1.135
**HEAT repeat-containing protein 3 OS=Mus musculus GN=Heatr3 PE=1 SV=1**	1.000	0.994	0.997	0.818	0.994	0.896
**Paralemmin-2 OS=Mus musculus GN=Palm2 PE=1 SV=1**	1.000	1.178	1.089	0.786	0.623	0.550
**Sec1 family domain-containing protein 1 OS=Mus musculus GN=Scfd1 PE=1 SV=1**	1.000	1.002	1.001	0.888	0.968	0.994
**Filamin-A OS=Mus musculus GN=Flna PE=1 SV=5**	1.160	1.286	1.223	1.487	1.599	1.492
**Cleavage and polyadenylation specificity factor subunit 7 OS=Mus musculus GN=Cpsf7 PE=1 SV=2**	1.000	2.011	1.505	3.905	8.975	5.187
**Exosome complex component MTR3 OS=Mus musculus GN=Exosc6 PE=1 SV=1**	1.000	1.029	1.014	0.736	1.386	0.889
**Isoleucine--tRNA ligase, cytoplasmic OS=Mus musculus GN=Iars PE=1 SV=2**	0.994	0.929	0.961	0.832	0.834	0.823
**COP9 signalosome complex subunit 7b OS=Mus musculus GN=Cops7b PE=1 SV=1**	1.000	0.983	0.991	0.721	0.562	0.757
**Dihydropteridine reductase OS=Mus musculus GN=Qdpr PE=1 SV=2**	1.000	1.114	1.057	1.919	1.878	1.668
**Axonemal dynein light intermediate polypeptide 1 OS=Mus musculus GN=Dnali1 PE=1 SV=1**	1.000	0.892	0.946	0.712	0.969	1.157
**Protein phosphatase methylesterase 1 OS=Mus musculus GN=Ppme1 PE=1 SV=5**	1.000	1.151	1.076	1.600	1.645	1.193
**ADP-ribose pyrophosphatase, mitochondrial OS=Mus musculus GN=Nudt9 PE=1 SV=1**	1.000	1.163	1.082	2.225	3.376	2.767

**Metalloreductase STEAP2 OS=Mus musculus GN=Steap2 PE=1 SV=1**	1.000	0.915	0.957	1.355	0.728	1.535
**3-ketoacyl-CoA thiolase, mitochondrial OS=Mus musculus GN=Acaa2 PE=1 SV=3**	1.000	0.819	0.909	0.803	0.766	0.663
**La-related protein 4 OS=Mus musculus GN=Larp4 PE=1 SV=2**	1.000	1.203	1.102	0.585	0.417	1.071
**Eukaryotic peptide chain release factor subunit 1 OS=Mus musculus GN=Etf1 PE=1 SV=4**	1.147	1.156	1.151	1.434	1.343	1.408
**Probable G-protein coupled receptor 21 OS=Mus musculus GN=Gpr21 PE=2 SV=1**	1.000	0.986	0.993	1.181	0.911	1.253
**Histone acetyltransferase type B catalytic subunit OS=Mus musculus GN=Hat1 PE=1 SV=1**	1.000	1.066	1.033	1.926	2.128	2.829
**TBC1 domain family member 17 OS=Mus musculus GN=Tbc1d17 PE=1 SV=2**	1.000	11.629	6.315	3.562	2.967	5.069
**Vacuolar protein sorting-associated protein 26B OS=Mus musculus GN=Vps26b PE=1 SV=1**	1.000	1.084	1.042	0.842	0.928	0.978
**Isoaspartyl peptidase/L-asparaginase OS=Mus musculus GN=Asrgl1 PE=1 SV=1**	1.000	0.725	0.862	0.616	0.519	1.233
**Prolyl endopeptidase-like OS=Mus musculus GN=Prepl PE=1 SV=1**	1.000	0.870	0.935	1.428	0.923	1.294
**Carbamoyl-phosphate synthase [ammonia], mitochondrial OS=Mus musculus GN=Cps1 PE=1 SV=2**	0.972	0.871	0.921	0.184	0.110	0.154
**Thimet oligopeptidase OS=Mus musculus GN=Thop1 PE=1 SV=1**	1.023	0.986	1.004	1.159	1.263	1.152
**Septin-11 OS=Mus musculus GN=Sept11 PE=1 SV=4**	1.007	1.042	1.024	0.920	0.911	0.929
**Kinetochore-associated protein 1 OS=Mus musculus GN=Kntc1 PE=1 SV=2**	1.000	1.041	1.021	0.990	0.993	0.892
**Sterile alpha motif domain-containing protein 3 OS=Mus musculus GN=Samd3 PE=2 SV=3**	1.024	0.952	0.988	1.416	0.748	1.285
**mRNA export factor OS=Mus musculus GN=Rae1 PE=1 SV=1**	1.000	0.890	0.945	0.663	0.553	0.505
**Ubiquitin-like modifier-activating enzyme 6 OS=Mus musculus GN=Uba6 PE=1 SV=1**	1.000	0.980	0.990	0.879	1.011	0.888
**NEDD8-activating enzyme E1 catalytic subunit OS=Mus musculus GN=Uba3 PE=1 SV=2**	1.045	1.036	1.041	1.071	1.132	0.992
**Protein ELFN1 OS=Mus musculus GN=Elfn1 PE=2 SV=1**	1.000	0.962	0.981	1.583	1.496	1.296
**Pre-rRNA-processing protein TSR2 homolog OS=Mus musculus GN=Tsr2 PE=1 SV=1**	1.000	0.848	0.924	0.934	0.839	1.069
**Liprin-beta-1 OS=Mus musculus GN=Ppfibp1 PE=1 SV=3**	1.000	0.961	0.981	0.691	0.612	0.735
**A disintegrin and metalloproteinase with thrombospondin motifs 2 OS=Mus musculus GN=Adamts2 PE=1 SV=2**	1.000	1.057	1.028	0.715	0.684	0.656
**Acetyl-CoA acetyltransferase, cytosolic OS=Mus musculus GN=Acat2 PE=1 SV=2**	1.018	0.795	0.906	1.055	1.016	0.970
**WD repeat-containing protein 37 OS=Mus musculus GN=Wdr37 PE=1 SV=1**	1.000	1.216	1.108	1.405	0.898	1.184
**Tsukushin OS=Mus musculus GN=Tsku PE=2 SV=2**	1.000	1.099	1.049	0.696	0.854	0.865
**Polyadenylate-binding protein 2 OS=Mus musculus GN=Pabpn1 PE=1 SV=3**	0.987	0.925	0.956	1.150	1.318	1.256
**Thioredoxin-like protein 1 OS=Mus musculus GN=Txnl1 PE=1 SV=3**	1.026	0.911	0.969	1.361	1.496	1.263
**tRNA (adenine(58)-N(1))-methyltransferase non-catalytic subunit TRM6 OS=Mus musculus GN=Trmt6 PE=1 SV=1**	1.068	2.305	1.686	4.314	4.422	3.262
**Complement C1s-B subcomponent OS=Mus musculus GN=C1sb PE=2 SV=1**	1.000	1.283	1.142	1.560	1.619	1.887
**Complement C1s-A subcomponent OS=Mus**	0.975	1.048	1.012	1.086	0.878	1.019

**musculus GN=C1sa PE=2 SV=2**						
**Complement C1r-A subcomponent OS=Mus musculus GN=C1ra PE=1 SV=1**	0.957	1.059	1.008	0.994	0.934	1.021
**Structural maintenance of chromosomes protein 4 OS=Mus musculus GN=Smc4 PE=1 SV=1**	1.000	1.088	1.044	0.670	1.101	0.677
**Protein phosphatase 1F OS=Mus musculus GN=Ppm1f PE=1 SV=1**	1.000	1.087	1.043	1.311	1.786	1.231
**Bifunctional glutamate/proline--tRNA ligase OS=Mus musculus GN=Eprs PE=1 SV=4**	1.000	0.971	0.985	1.195	1.034	1.311
**Cell division cycle and apoptosis regulator protein 1 OS=Mus musculus GN=Ccar1 PE=1 SV=1**	1.000	0.993	0.997	1.198	1.334	1.446
**Phosphoglycolate phosphatase OS=Mus musculus GN=Pgp PE=1 SV=1**	1.000	0.819	0.909	0.898	1.064	1.003
**Epsin-2 OS=Mus musculus GN=Epn2 PE=1 SV=1**	1.000	0.725	0.862	0.361	0.597	0.043
**PDZ and LIM domain protein 5 OS=Mus musculus GN=Pdlim5 PE=1 SV=4**	1.080	1.055	1.068	1.255	1.405	1.233
**Glycogen phosphorylase, brain form OS=Mus musculus GN=Pygb PE=1 SV=3**	1.028	1.243	1.135	1.398	1.344	1.317
**Fermitin family homolog 2 OS=Mus musculus GN=Fermt2 PE=1 SV=1**	0.852	0.993	0.923	1.052	1.218	1.116
**Coatomer subunit alpha OS=Mus musculus GN=Copa PE=1 SV=2**	0.970	0.950	0.960	0.994	0.998	1.029
**Biotinidase OS=Mus musculus GN=Btd PE=1 SV=2**	1.000	0.766	0.883	0.452	0.679	0.568
**Protein arginine N-methyltransferase 5 OS=Mus musculus GN=Prmt5 PE=1 SV=3**	1.000	0.789	0.894	1.025	0.755	0.749
**Protein argonaute-2 OS=Mus musculus GN=Ago2 PE=1 SV=3**	1.000	0.859	0.929	0.726	0.645	0.893
**Hydroxymethylglutaryl-CoA synthase, cytoplasmic OS=Mus musculus GN=Hmgcs1 PE=1 SV=1**	1.000	0.883	0.942	1.301	1.201	1.117
**Parafibromin OS=Mus musculus GN=Cdc73 PE=1 SV=1**	1.000	0.912	0.956	1.301	0.988	0.614
**Eukaryotic translation initiation factor 3 subunit B OS=Mus musculus GN=Eif3b PE=1 SV=1**	0.836	0.993	0.915	1.336	1.345	1.314
**Translation machinery-associated protein 7 OS=Mus musculus GN=Tma7 PE=3 SV=1**	1.000	1.490	1.245	5.524	2.859	3.104
**GDP-mannose 4,6 dehydratase OS=Mus musculus GN=Gmds PE=1 SV=1**	1.000	1.032	1.016	1.115	1.221	0.987
**Protein unc-13 homolog C OS=Mus musculus GN=Unc13c PE=1 SV=3**	1.000	1.100	1.050	0.934	1.045	0.860
**Aldose 1-epimerase OS=Mus musculus GN=Galm PE=1 SV=1**	0.840	0.886	0.863	1.498	1.815	1.994
**Pyridoxal kinase OS=Mus musculus GN=Pdxk PE=1 SV=1**	1.000	0.922	0.961	1.342	0.921	0.743
**CCA tRNA nucleotidyltransferase 1, mitochondrial OS=Mus musculus GN=Trnt1 PE=1 SV=1**	1.000	1.070	1.035	1.452	1.426	1.320
**Dynamin-1-like protein OS=Mus musculus GN=Dnm1l PE=1 SV=2**	1.000	0.931	0.966	1.025	0.885	1.047
**Pleckstrin homology-like domain family B member 2 OS=Mus musculus GN=Phldb2 PE=1 SV=2**	1.000	0.982	0.991	0.725	0.488	0.611
**Serine/threonine-protein kinase Nek9 OS=Mus musculus GN=Nek9 PE=1 SV=2**	1.000	0.923	0.961	0.743	1.145	1.046
**Placenta-specific protein 9 OS=Mus musculus GN=Plac9 PE=2 SV=1**	1.000	0.920	0.960	0.198	0.125	0.219
**Actin-binding protein anillin OS=Mus musculus GN=Anln PE=1 SV=2**	1.000	0.930	0.965	0.712	1.040	1.011
**Beta-mannosidase OS=Mus musculus GN=Manba PE=1 SV=1**	1.000	0.793	0.896	0.825	0.380	0.653
**Zinc finger protein 668 OS=Mus musculus GN=Znf668 PE=2 SV=1**	1.000	1.376	1.188	2.052	0.988	1.318
**Matrin-3 OS=Mus musculus GN=Matr3 PE=1 SV=1**	1.000	1.004	1.002	1.072	1.504	1.053

**DNA/RNA-binding protein KIN17 OS=Mus musculus GN=Kin PE=1 SV=1**	1.000	0.828	0.914	0.808	0.651	0.890
**7SK snRNA methylphosphate capping enzyme OS=Mus musculus GN=Mepce PE=1 SV=2**	1.000	3.771	2.386	9.639	10.589	9.942
**Protein LZIC OS=Mus musculus GN=Lzic PE=1 SV=1**	1.036	0.863	0.949	0.751	1.253	0.883
**EMILIN-2 OS=Mus musculus GN=Emilin2 PE=1 SV=1**	1.000	0.942	0.971	0.872	0.726	0.984
**Latent-transforming growth factor beta-binding protein 4 OS=Mus musculus GN=Ltbp4 PE=1 SV=2**	0.929	0.834	0.882	0.526	0.471	0.488
**COMM domain-containing protein 1 OS=Mus musculus GN=Commd1 PE=1 SV=2**	1.000	0.757	0.879	0.462	0.602	0.446
**Collectin-12 OS=Mus musculus GN=Colec12 PE=1 SV=1**	1.000	0.995	0.997	1.144	1.006	1.167
**NAD(P)H-hydrate epimerase OS=Mus musculus GN=Apoa1bp PE=1 SV=1**	0.992	1.047	1.019	1.074	1.181	0.975
**Splicing factor 3A subunit 1 OS=Mus musculus GN=Sf3a1 PE=1 SV=1**	1.000	0.949	0.975	1.248	1.242	1.271
**Out at first protein homolog OS=Mus musculus GN=Oaf PE=2 SV=1**	1.022	1.147	1.084	3.045	2.540	2.339
**DNA-binding death effector domain-containing protein 2 OS=Mus musculus GN=Dedd2 PE=2 SV=1**	1.000	0.930	0.965	0.876	0.853	1.160
**Eukaryotic translation initiation factor 3 subunit L OS=Mus musculus GN=Eif3l PE=1 SV=1**	1.052	1.090	1.071	1.248	1.197	1.289
**Splicing factor 3B subunit 4 OS=Mus musculus GN=Sf3b4 PE=1 SV=1**	0.899	0.967	0.933	1.262	1.201	1.360
**Aminoacyl tRNA synthase complex-interacting multifunctional protein 2 OS=Mus musculus GN=Aimp2 PE=1 SV=2**	1.000	1.027	1.014	1.159	0.872	0.862
**Bleomycin hydrolase OS=Mus musculus GN=Blmh PE=1 SV=1**	1.000	0.909	0.955	1.162	0.999	1.003
**Eukaryotic peptide chain release factor GTP-binding subunit ERF3A OS=Mus musculus GN=Gspt1 PE=1 SV=2**	1.000	0.894	0.947	1.158	1.242	1.245
**Heterogeneous nuclear ribonucleoprotein L OS=Mus musculus GN=Hnrnpl PE=1 SV=2**	1.089	1.225	1.157	1.749	1.965	2.156
**General transcription factor IIF subunit 2 OS=Mus musculus GN=Gtf2f2 PE=1 SV=1**	1.000	0.893	0.946	1.255	1.132	1.008
**Sulfatase-modifying factor 1 OS=Mus musculus GN=Sumf1 PE=1 SV=2**	1.000	0.974	0.987	1.014	0.769	1.214
**Epiplakin OS=Mus musculus GN=Eppk1 PE=1 SV=2**	1.000	0.927	0.964	1.255	0.905	1.565
**Acylamino-acid-releasing enzyme OS=Mus musculus GN=Apeh PE=1 SV=3**	1.000	1.050	1.025	0.803	0.657	0.677
**ERO1-like protein alpha OS=Mus musculus GN=Ero1a PE=1 SV=2**	1.000	0.906	0.953	0.546	0.563	0.772
**Dedicator of cytokinesis protein 7 OS=Mus musculus GN=Dock7 PE=1 SV=3**	1.000	1.434	1.217	1.233	1.563	1.779
**Eukaryotic translation initiation factor 3 subunit C OS=Mus musculus GN=Eif3c PE=1 SV=1**	1.064	1.175	1.119	1.501	1.381	1.516
**Niban-like protein 1 OS=Mus musculus GN=Fam129b PE=1 SV=2**	1.000	1.203	1.102	1.519	1.681	1.540
**Putative hydroxypyruvate isomerase OS=Mus musculus GN=Hyi PE=1 SV=2**	1.000	0.906	0.953	0.750	0.683	0.757
**Angiopoietin-related protein 7 OS=Mus musculus GN=Angptl7 PE=2 SV=1**	1.000	1.009	1.004	1.411	1.006	1.563
**Spartin OS=Mus musculus GN=Spg20 PE=1 SV=1**	1.000	1.123	1.062	1.014	0.782	0.819
**Coiled-coil domain-containing protein 80 OS=Mus musculus GN=Ccdc80 PE=1 SV=2**	1.050	0.979	1.014	0.642	0.511	0.619
**Tripartite motif-containing protein 29 OS=Mus musculus GN=Trim29 PE=1 SV=1**	1.000	0.913	0.956	0.633	0.598	0.639
**Cell surface glycoprotein MUC18 OS=Mus**	1.000	0.995	0.997	0.994	0.821	1.017

musculus GN=Mcam PE=1 SV=1						
Ubiquilin-1 OS=Mus musculus GN=Ubqln1 PE=1 SV=1	0.959	0.958	0.959	1.106	1.034	0.932
Paraspeckle component 1 OS=Mus musculus GN=Pspc1 PE=1 SV=1	1.000	1.057	1.029	1.225	1.195	1.347
COMM domain-containing protein 5 OS=Mus musculus GN=Commd5 PE=1 SV=1	1.000	1.183	1.092	2.481	2.636	2.386
Coiled-coil domain-containing protein 58 OS=Mus musculus GN=Ccdc58 PE=1 SV=1	1.000	0.781	0.891	0.556	0.658	0.796
CD109 antigen OS=Mus musculus GN=Cd109 PE=1 SV=1	1.000	0.857	0.929	0.731	0.713	0.809
Ataxin-7 OS=Mus musculus GN=Atxn7 PE=1 SV=2	1.000	1.056	1.028	1.235	0.840	0.946
SH3 domain-containing kinase-binding protein 1 OS=Mus musculus GN=Sh3kbp1 PE=1 SV=1	1.076	1.084	1.080	1.381	1.793	1.049
Beta-centractin OS=Mus musculus GN=Actr1b PE=1 SV=1	1.000	0.995	0.998	1.256	1.155	1.327
COP9 signalosome complex subunit 8 OS=Mus musculus GN=Cops8 PE=1 SV=1	1.072	1.013	1.042	1.243	1.175	1.148
NEDD8-activating enzyme E1 regulatory subunit OS=Mus musculus GN=Nae1 PE=1 SV=1	1.000	1.074	1.037	1.323	1.203	1.423
Solute carrier family 22 member 6 OS=Mus musculus GN=Slc22a6 PE=1 SV=1	1.000	1.168	1.084	0.866	0.833	6.185
U6 snRNA-associated Sm-like protein LSm1 OS=Mus musculus GN=Lsm1 PE=1 SV=1	1.000	0.909	0.955	0.824	0.533	0.615
Soluble calcium-activated nucleotidase 1 OS=Mus musculus GN=Cant1 PE=2 SV=1	1.000	0.872	0.936	0.694	0.671	0.700
Cystathionine gamma-lyase OS=Mus musculus GN=Cth PE=1 SV=1	0.907	1.041	0.974	1.714	1.231	1.491
Aminopeptidase B OS=Mus musculus GN=Rnpep PE=1 SV=2	1.000	1.514	1.257	1.779	1.475	1.970
Huntingtin-interacting protein 1 OS=Mus musculus GN=Hip1 PE=1 SV=2	1.000	1.156	1.078	1.294	0.776	1.211
Myosin-9 OS=Mus musculus GN=Myh9 PE=1 SV=4	0.914	0.950	0.932	0.816	0.995	0.812
Vigilin OS=Mus musculus GN=Hdlbp PE=1 SV=1	1.000	0.769	0.885	0.573	0.404	0.600
26S proteasome non-ATPase regulatory subunit 2 OS=Mus musculus GN=Psmd2 PE=1 SV=1	1.071	1.034	1.053	1.061	1.043	1.053
ATP-dependent RNA helicase DDX39A OS=Mus musculus GN=Ddx39a PE=1 SV=1	1.062	1.115	1.088	1.318	1.407	1.439
Meteorin-like protein OS=Mus musculus GN=Metrnl PE=1 SV=1	1.000	0.969	0.984	0.873	0.589	0.882
Keratin, type II cytoskeletal 79 OS=Mus musculus GN=Krt79 PE=1 SV=2	1.000	0.758	0.879	0.158	0.273	0.205
LIM and cysteine-rich domains protein 1 OS=Mus musculus GN=Lmcd1 PE=1 SV=1	1.000	0.931	0.965	0.947	0.820	0.972
EPM2A-interacting protein 1 OS=Mus musculus GN=Epm2aip1 PE=1 SV=1	1.000	1.194	1.097	1.016	0.817	0.948
Heterogeneous nuclear ribonucleoprotein U OS=Mus musculus GN=Hnrnpu PE=1 SV=1	0.982	1.005	0.993	1.353	1.332	1.334
RNA-binding protein 39 OS=Mus musculus GN=Rbm39 PE=1 SV=2	0.042	5.877	2.960	2.144	9.724	10.959
Ras-related protein Rab-40C OS=Mus musculus GN=Rab40c PE=1 SV=1	1.000	0.987	0.993	1.283	0.888	1.537
Filamin-C OS=Mus musculus GN=Flnc PE=1 SV=3	1.054	1.092	1.073	1.104	1.250	1.050
Splicing factor, proline- and glutamine-rich OS=Mus musculus GN=Sfpq PE=1 SV=1	1.111	1.255	1.183	2.387	3.087	2.297
Anamorsin OS=Mus musculus GN=Ciapin1 PE=1 SV=1	1.000	1.398	1.199	0.999	1.011	1.180
Cytosolic acyl coenzyme A thioester hydrolase OS=Mus musculus GN=Acot7 PE=1 SV=2	1.000	1.117	1.059	2.000	2.964	2.252
Ras-related protein Rab-14 OS=Mus musculus GN=Rab14 PE=1 SV=3	1.000	1.027	1.013	1.207	1.612	1.032

**Isochorismatase domain-containing protein 1 OS=Mus musculus GN=Isoc1 PE=1 SV=1**	1.000	0.985	0.992	1.317	1.124	1.304
**Ester hydrolase C11orf54 homolog OS=Mus musculus PE=1 SV=1**	1.000	0.832	0.916	0.831	0.940	1.050
**ATP-citrate synthase OS=Mus musculus GN=Acly PE=1 SV=1**	1.078	1.094	1.086	1.214	1.389	1.369
**Sorting nexin-9 OS=Mus musculus GN=Snx9 PE=1 SV=1**	1.000	0.953	0.976	1.117	0.933	1.300
**Ribonuclease inhibitor OS=Mus musculus GN=Rnh1 PE=1 SV=1**	0.950	0.962	0.956	1.266	1.132	1.129
**Polyglutamine-binding protein 1 OS=Mus musculus GN=Pqbp1 PE=1 SV=1**	1.000	1.153	1.076	1.610	2.521	3.015
**Basic leucine zipper and W2 domain-containing protein 2 OS=Mus musculus GN=Bzw2 PE=1 SV=1**	1.000	1.369	1.185	2.067	3.052	2.464
**RNA binding motif protein, X-linked-like-1 OS=Mus musculus GN=Rbmxl1 PE=2 SV=1**	1.000	0.778	0.889	1.515	2.016	1.833
**ATP-dependent RNA helicase DDX1 OS=Mus musculus GN=Ddx1 PE=1 SV=1**	0.995	0.997	0.996	1.001	1.316	1.255
**Protein BRICK1 OS=Mus musculus GN=Brk1 PE=1 SV=1**	1.000	1.291	1.145	2.269	2.826	3.827
**Protein FAM3C OS=Mus musculus GN=Fam3c PE=1 SV=1**	1.000	1.219	1.109	1.242	1.598	0.943
**SH3 domain-binding glutamic acid-rich-like protein 3 OS=Mus musculus GN=Sh3bgrl3 PE=1 SV=1**	1.000	1.048	1.024	1.065	0.821	0.683
**Ubiquitin-associated protein 2 OS=Mus musculus GN=Ubap2 PE=1 SV=1**	1.000	0.911	0.956	1.441	1.166	1.027
**Ubiquitin-like protein 7 OS=Mus musculus GN=Ubl7 PE=1 SV=2**	1.000	1.004	1.002	1.049	0.976	1.878
**Thioredoxin domain-containing protein 5 OS=Mus musculus GN=Txndc5 PE=1 SV=2**	1.042	1.133	1.087	1.216	1.248	1.261
**Far upstream element-binding protein 1 OS=Mus musculus GN=Fubp1 PE=1 SV=1**	1.000	0.979	0.990	1.289	1.548	1.169
**Eukaryotic translation initiation factor 3 subunit H OS=Mus musculus GN=Eif3h PE=1 SV=1**	0.896	0.895	0.896	1.097	1.494	1.280
**Glycine cleavage system H protein, mitochondrial OS=Mus musculus GN=Gcsh PE=1 SV=2**	1.000	0.874	0.937	0.763	1.377	0.858
**DnaJ homolog subfamily C member 9 OS=Mus musculus GN=Dnajc9 PE=1 SV=2**	1.000	0.978	0.989	0.938	1.070	1.122
**Serine protease inhibitor A3N OS=Mus musculus GN=Serpina3n PE=1 SV=1**	0.966	0.963	0.965	0.594	0.552	0.643
**Tyrosine--tRNA ligase, cytoplasmic OS=Mus musculus GN=Yars PE=1 SV=3**	1.000	0.967	0.983	1.032	1.284	1.182
**Neurocalcin-delta OS=Mus musculus GN=Ncald PE=1 SV=4**	1.000	0.777	0.888	0.432	0.610	0.428
**Golgi membrane protein 1 OS=Mus musculus GN=Golm1 PE=1 SV=2**	1.000	0.880	0.940	0.897	0.727	0.630
**Brain acid soluble protein 1 OS=Mus musculus GN=Basp1 PE=1 SV=3**	0.932	1.020	0.976	1.603	1.949	1.405
**Importin-9 OS=Mus musculus GN=Ipo9 PE=1 SV=3**	1.000	0.820	0.910	0.730	0.550	0.441
**Atlastin-3 OS=Mus musculus GN=Atl3 PE=1 SV=1**	1.000	0.834	0.917	0.599	0.520	0.993
**Twinfilin-1 OS=Mus musculus GN=Twf1 PE=1 SV=2**	0.961	1.020	0.990	1.531	1.139	1.373
**Methyltransferase-like protein 13 OS=Mus musculus GN=Mettl13 PE=1 SV=1**	1.000	0.816	0.908	0.966	1.517	1.215
**DnaJ homolog subfamily C member 3 OS=Mus musculus GN=Dnajc3 PE=1 SV=1**	0.929	0.911	0.920	0.938	0.758	0.903
**Glyoxylate reductase/hydroxypyruvate reductase OS=Mus musculus GN=Grhpr PE=1 SV=1**	1.000	0.991	0.995	0.488	0.650	0.811
**UTP--glucose-1-phosphate uridylyltransferase OS=Mus musculus GN=Ugp2 PE=1 SV=3**	0.977	0.918	0.947	1.251	1.124	1.171

**Prolow-density lipoprotein receptor-related protein 1 OS=Mus musculus GN=Lrp1 PE=1 SV=1**	1.000	1.004	1.002	0.787	0.768	1.012
**Retinoid-inducible serine carboxypeptidase OS=Mus musculus GN=Scpep1 PE=1 SV=2**	1.029	0.977	1.003	1.154	0.957	1.109
**FACT complex subunit SPT16 OS=Mus musculus GN=Supt16h PE=1 SV=2**	0.948	0.905	0.926	1.284	1.232	1.345
**Farnesyl pyrophosphate synthase OS=Mus musculus GN=Fdps PE=1 SV=1**	1.151	0.999	1.075	1.122	1.356	1.310
**Protein bicaudal D homolog 2 OS=Mus musculus GN=Bicd2 PE=1 SV=1**	1.000	0.924	0.962	0.688	0.754	1.133
**TAR DNA-binding protein 43 OS=Mus musculus GN=Tardbp PE=1 SV=1**	0.876	1.042	0.959	1.687	1.745	1.509
**Heterogeneous nuclear ribonucleoprotein L-like OS=Mus musculus GN=Hnrnpll PE=1 SV=3**	1.000	1.117	1.058	1.383	1.151	1.148
**Kelch domain-containing protein 4 OS=Mus musculus GN=Klhdc4 PE=2 SV=2**	1.000	1.174	1.087	1.185	1.634	1.575
**Exosome complex component RRP41 OS=Mus musculus GN=Exosc4 PE=1 SV=3**	1.000	1.332	1.166	0.820	0.939	1.528
**Splicing factor 3B subunit 3 OS=Mus musculus GN=Sf3b3 PE=1 SV=1**	1.080	1.012	1.046	1.113	1.112	1.162
**Protein FAM49B OS=Mus musculus GN=Fam49b PE=1 SV=1**	1.000	0.940	0.970	1.043	0.774	0.886
**Aspartate--tRNA ligase, cytoplasmic OS=Mus musculus GN=Dars PE=1 SV=2**	1.000	1.008	1.004	1.813	1.174	1.816
**Tubulin beta-6 chain OS=Mus musculus GN=Tubb6 PE=1 SV=1**	1.083	1.067	1.075	1.251	1.370	1.315
**CAP-Gly domain-containing linker protein 1 OS=Mus musculus GN=Clip1 PE=1 SV=1**	1.000	0.597	0.798	0.872	0.922	0.781
**Probable 28S rRNA (cytosine-C(5))-methyltransferase OS=Mus musculus GN=Nop2 PE=1 SV=1**	1.000	1.052	1.026	1.002	1.116	0.998
**Protein disulfide-isomerase A6 OS=Mus musculus GN=Pdia6 PE=1 SV=3**	1.049	1.096	1.073	1.057	1.006	1.096
**Keratin, type II cytoskeletal 5 OS=Mus musculus GN=Krt5 PE=1 SV=1**	1.000	1.053	1.027	0.355	0.644	0.510
**Mannose-6-phosphate isomerase OS=Mus musculus GN=Mpi PE=1 SV=1**	1.000	1.299	1.149	1.653	1.617	1.556
**Zinc finger transcription factor Trps1 OS=Mus musculus GN=Trps1 PE=1 SV=1**	1.000	1.734	1.367	0.699	1.022	1.717
**Transaldolase OS=Mus musculus GN=Taldo1 PE=1 SV=2**	1.045	0.988	1.017	1.667	2.085	1.453
**Heterogeneous nuclear ribonucleoprotein A/B OS=Mus musculus GN=Hnrnpab PE=1 SV=1**	0.988	0.974	0.981	1.828	2.812	1.979
**THUMP domain-containing protein 1 OS=Mus musculus GN=Thumpd1 PE=1 SV=1**	1.000	0.909	0.954	1.712	1.707	1.271
**Nuclear receptor-binding protein OS=Mus musculus GN=Nrbp1 PE=1 SV=1**	1.000	0.962	0.981	0.956	1.387	1.125
**Diphosphomevalonate decarboxylase OS=Mus musculus GN=Mvd PE=1 SV=2**	0.817	0.865	0.841	1.174	1.118	1.302
**PC4 and SFRS1-interacting protein OS=Mus musculus GN=Psip1 PE=1 SV=1**	1.084	1.063	1.073	1.652	1.634	1.401
**26S proteasome non-ATPase regulatory subunit 6 OS=Mus musculus GN=Psmd6 PE=1 SV=1**	0.944	0.968	0.956	1.004	1.249	1.089
**Ras-related protein Rap-1b OS=Mus musculus GN=Rap1b PE=1 SV=2**	1.000	1.109	1.055	1.150	1.607	1.240
**Tubulointerstitial nephritis antigen-like OS=Mus musculus GN=Tinagl1 PE=1 SV=1**	1.000	1.558	1.279	0.573	0.670	0.912
**Aminoacylase-1 OS=Mus musculus GN=Acy1 PE=1 SV=1**	1.000	0.893	0.946	0.717	0.689	1.175
**Golgi reassembly-stacking protein 2 OS=Mus musculus GN=Gorasp2 PE=1 SV=3**	0.945	1.172	1.058	1.211	1.088	1.747

**Eukaryotic translation initiation factor 3 subunit M OS=Mus musculus GN=Eif3m PE=1 SV=1**	1.007	1.003	1.005	1.039	1.011	1.139
**Actin-related protein 3 OS=Mus musculus GN=Actr3 PE=1 SV=3**	0.966	1.018	0.992	1.120	1.070	1.140
**EMILIN-1 OS=Mus musculus GN=Emilin1 PE=1 SV=1**	0.977	0.969	0.973	0.723	0.629	0.812
**Non-POU domain-containing octamer-binding protein OS=Mus musculus GN=Nono PE=1 SV=3**	0.971	0.774	0.872	0.829	0.864	1.006
**Plastin-3 OS=Mus musculus GN=Pls3 PE=1 SV=3**	1.024	1.144	1.084	1.574	1.694	1.418
**Phosphoserine aminotransferase OS=Mus musculus GN=Psat1 PE=1 SV=1**	1.012	0.991	1.001	1.404	1.280	1.417
**von Willebrand factor A domain-containing protein 5A OS=Mus musculus GN=Vwa5a PE=1 SV=2**	1.000	1.161	1.080	1.211	1.078	1.248
**RNA-binding protein 10 OS=Mus musculus GN=Rbm10 PE=1 SV=1**	1.000	0.915	0.957	1.222	0.906	1.215
**Aconitate hydratase, mitochondrial OS=Mus musculus GN=Aco2 PE=1 SV=1**	1.000	0.911	0.956	0.872	1.279	0.850
**Dynactin subunit 2 OS=Mus musculus GN=Dctn2 PE=1 SV=3**	0.965	0.999	0.982	1.164	1.090	1.009
**Dipeptidyl peptidase 3 OS=Mus musculus GN=Dpp3 PE=1 SV=2**	1.080	1.117	1.098	0.961	0.949	1.076
**Clathrin interactor 1 OS=Mus musculus GN=Clint1 PE=1 SV=2**	1.011	1.233	1.122	3.697	3.017	2.950
**Pre-mRNA-processing factor 19 OS=Mus musculus GN=Prpf19 PE=1 SV=1**	1.000	1.004	1.002	1.491	1.680	1.449
**Nicotinamide phosphoribosyltransferase OS=Mus musculus GN=Nampt PE=1 SV=1**	0.869	1.013	0.941	1.749	2.170	1.759
**Eukaryotic translation initiation factor 2 subunit 2 OS=Mus musculus GN=Eif2s2 PE=1 SV=1**	0.981	0.996	0.989	1.508	1.748	1.489
**Hsc70-interacting protein OS=Mus musculus GN=St13 PE=1 SV=1**	0.994	1.037	1.016	2.104	2.730	2.312
**Methionine adenosyltransferase 2 subunit beta OS=Mus musculus GN=Mat2b PE=1 SV=1**	1.000	0.710	0.855	1.300	0.624	0.922
**Electron transfer flavoprotein subunit alpha, mitochondrial OS=Mus musculus GN=Etfa PE=1 SV=2**	1.000	1.000	1.000	1.000	1.000	15.740
**N(G),N(G)-dimethylarginine dimethylaminohydrolase 2 OS=Mus musculus GN=Ddah2 PE=1 SV=1**	1.000	0.992	0.996	1.120	1.072	1.276
**Translation initiation factor eIF-2B subunit beta OS=Mus musculus GN=Eif2b2 PE=1 SV=1**	1.000	1.587	1.294	2.900	3.473	3.965
**Tissue alpha-L-fucosidase OS=Mus musculus GN=Fuca1 PE=1 SV=1**	1.000	1.601	1.300	1.757	1.143	1.735
**Deoxyhypusine hydroxylase OS=Mus musculus GN=Dohh PE=1 SV=2**	1.000	0.811	0.905	0.705	1.069	1.131
**GrpE protein homolog 1, mitochondrial OS=Mus musculus GN=Grpel1 PE=1 SV=1**	1.041	0.892	0.966	0.627	0.939	0.735
**Phosphoserine phosphatase OS=Mus musculus GN=Psph PE=1 SV=1**	1.000	0.990	0.995	1.196	1.182	1.025
**Protein deglycase DJ-1 OS=Mus musculus GN=Park7 PE=1 SV=1**	1.058	1.031	1.044	1.292	1.217	1.268
**Multiple myeloma tumor-associated protein 2 homolog OS=Mus musculus GN=Mmtag2 PE=2 SV=1**	1.000	0.964	0.982	1.271	0.901	1.518
**Cytoplasmic protein NCK1 OS=Mus musculus GN=Nck1 PE=1 SV=1**	1.000	3.675	2.337	16.973	15.121	12.875
**Mammalian ependymin-related protein 1 OS=Mus musculus GN=Epdr1 PE=1 SV=1**	1.000	1.165	1.082	0.906	0.940	1.165
**Nuclear autoantigenic sperm protein OS=Mus musculus GN=Nasp PE=1 SV=2**	1.000	0.888	0.944	0.848	1.239	0.999

**Lysine--tRNA ligase OS=Mus musculus GN=Kars PE=1 SV=1**	1.075	1.077	1.076	1.677	1.499	1.526
**Serrate RNA effector molecule homolog OS=Mus musculus GN=Srrt PE=1 SV=1**	0.883	0.991	0.937	1.106	0.754	1.083
**Tudor domain-containing protein 1 OS=Mus musculus GN=Tdrd1 PE=1 SV=2**	1.000	0.858	0.929	1.058	1.746	1.232
**Splicing factor 3B subunit 1 OS=Mus musculus GN=Sf3b1 PE=1 SV=1**	1.000	0.988	0.994	1.115	0.914	1.304
**Reticulon-4 OS=Mus musculus GN=Rtn4 PE=1 SV=2**	1.000	1.010	1.005	0.932	1.077	1.089
**Transmembrane glycoprotein NMB OS=Mus musculus GN=Gpnmb PE=1 SV=2**	1.000	1.082	1.041	0.766	0.875	0.848
**Opioid growth factor receptor OS=Mus musculus GN=Ogfr PE=1 SV=1**	1.000	1.091	1.046	1.385	1.252	1.365
**Ribosome-binding protein 1 OS=Mus musculus GN=Rrbp1 PE=1 SV=2**	0.899	0.897	0.898	0.759	0.694	0.864
**Rho GDP-dissociation inhibitor 1 OS=Mus musculus GN=Arhgdia PE=1 SV=3**	1.119	1.117	1.118	1.535	1.619	1.373
**Pre-mRNA-processing-splicing factor 8 OS=Mus musculus GN=Prpf8 PE=1 SV=2**	1.000	1.080	1.040	1.305	1.037	0.659
**60S ribosomal protein L17 OS=Mus musculus GN=Rpl17 PE=1 SV=3**	1.000	1.028	1.014	1.668	1.860	1.469
**Lactoylglutathione lyase OS=Mus musculus GN=Glo1 PE=1 SV=3**	1.067	1.086	1.077	2.242	2.939	2.106
**Glyoxalase domain-containing protein 4 OS=Mus musculus GN=Glod4 PE=1 SV=1**	1.000	0.993	0.997	1.147	1.137	1.127
**Actin-related protein 2/3 complex subunit 5 OS=Mus musculus GN=Arpc5 PE=1 SV=3**	0.920	0.845	0.882	1.058	0.847	0.923
**Cytosol aminopeptidase OS=Mus musculus GN=Lap3 PE=1 SV=3**	0.965	1.015	0.990	2.030	2.245	1.989
**Ribonuclease T2 OS=Mus musculus GN=Rnaset2 PE=1 SV=1**	0.916	0.888	0.902	0.917	0.694	1.065
**Myosin regulatory light polypeptide 9 OS=Mus musculus GN=Myl9 PE=1 SV=3**	1.011	0.975	0.993	1.107	1.509	0.953
**Neudesin OS=Mus musculus GN=Nenf PE=1 SV=1**	1.000	1.006	1.003	1.003	0.675	0.882
**6-phosphogluconolactonase OS=Mus musculus GN=Pgls PE=1 SV=1**	1.249	1.334	1.292	2.864	2.601	2.927
**S-methyl-5'-thioadenosine phosphorylase OS=Mus musculus GN=Mtap PE=1 SV=1**	1.081	0.922	1.001	1.023	1.037	1.252
**Thioredoxin domain-containing protein 9 OS=Mus musculus GN=Txndc9 PE=1 SV=1**	1.000	0.991	0.996	1.275	1.592	1.466
**Protein CutA OS=Mus musculus GN=Cuta PE=1 SV=3**	1.000	0.768	0.884	0.560	0.543	0.623
**UPF0568 protein C14orf166 homolog OS=Mus musculus PE=1 SV=1**	0.933	0.924	0.928	1.366	1.361	1.404
**Cleavage and polyadenylation specificity factor subunit 5 OS=Mus musculus GN=Nudt21 PE=1 SV=1**	1.000	0.944	0.972	2.715	2.067	2.602
**Glia maturation factor beta OS=Mus musculus GN=Gmfb PE=1 SV=3**	1.145	0.953	1.049	1.172	1.529	1.019
**Coactosin-like protein OS=Mus musculus GN=Cotl1 PE=1 SV=3**	1.000	0.950	0.975	0.577	0.886	0.839
**Density-regulated protein OS=Mus musculus GN=Denr PE=1 SV=1**	1.062	0.982	1.022	1.392	1.748	1.298
**RWD domain-containing protein 1 OS=Mus musculus GN=Rwdd1 PE=1 SV=1**	1.000	0.717	0.858	0.588	0.984	0.882
**Thioredoxin domain-containing protein 17 OS=Mus musculus GN=Txndc17 PE=1 SV=1**	0.911	0.859	0.885	1.046	1.127	0.993
**Glutaredoxin-3 OS=Mus musculus GN=Glrx3 PE=1 SV=1**	1.014	0.958	0.986	1.464	1.365	1.316
**40S ribosomal protein S21 OS=Mus musculus**	0.976	1.004	0.990	1.444	1.528	1.358

GN=Rps21 PE=1 SV=1						
Methylthioribose-1-phosphate isomerase OS=Mus musculus GN=Mri1 PE=1 SV=1	1.000	0.944	0.972	0.768	0.800	1.159
Thioredoxin domain-containing protein 12 OS=Mus musculus GN=Txndc12 PE=1 SV=1	1.000	0.841	0.920	0.671	0.784	0.658
Microtubule-associated proteins 1A/1B light chain 3B OS=Mus musculus GN=Map1lc3b PE=1 SV=3	1.000	0.882	0.941	1.716	2.482	2.187
14-3-3 protein beta/alpha OS=Mus musculus GN=Ywhab PE=1 SV=3	1.079	1.092	1.086	1.182	1.287	1.356
Synaptobrevin homolog YKT6 OS=Mus musculus GN=Ykt6 PE=1 SV=1	1.000	1.484	1.242	1.493	1.085	1.736
Heat shock factor-binding protein 1 OS=Mus musculus GN=Hsbp1 PE=1 SV=1	1.000	0.833	0.916	1.027	1.226	1.084
26S proteasome non-ATPase regulatory subunit 9 OS=Mus musculus GN=Psmd9 PE=1 SV=1	1.000	1.106	1.053	1.332	1.450	1.803
Peptidyl-prolyl cis-trans isomerase D OS=Mus musculus GN=Ppid PE=1 SV=3	1.000	0.953	0.976	1.485	1.746	1.450
Acyl carrier protein, mitochondrial OS=Mus musculus GN=Ndufab1 PE=1 SV=1	1.000	0.978	0.989	0.371	0.466	0.014
WD repeat domain phosphoinositide-interacting protein 3 OS=Mus musculus GN=Wdr45b PE=1 SV=2	1.000	0.806	0.903	1.061	1.015	1.934
Huntingtin-interacting protein K OS=Mus musculus GN=Hypk PE=1 SV=2	1.000	1.001	1.000	0.996	1.646	0.922
V-type proton ATPase subunit G 1 OS=Mus musculus GN=Atp6v1g1 PE=1 SV=3	1.000	1.131	1.066	0.928	0.983	1.416
H/ACA ribonucleoprotein complex subunit 2 OS=Mus musculus GN=Nhp2 PE=1 SV=1	1.004	0.984	0.994	0.802	0.809	1.096
SNW domain-containing protein 1 OS=Mus musculus GN=Snw1 PE=1 SV=3	1.000	1.087	1.044	0.817	0.678	0.638
Ran-binding protein 3 OS=Mus musculus GN=Ranbp3 PE=1 SV=2	1.000	0.940	0.970	0.654	0.661	0.724
Structural maintenance of chromosomes protein 1A OS=Mus musculus GN=Smc1a PE=1 SV=4	1.000	1.065	1.033	1.276	1.012	1.169
Actin-related protein 2/3 complex subunit 2 OS=Mus musculus GN=Arpc2 PE=1 SV=3	1.000	1.061	1.030	1.074	1.183	1.371
Structural maintenance of chromosomes protein 3 OS=Mus musculus GN=Smc3 PE=1 SV=2	1.000	0.862	0.931	0.669	0.573	0.720
Ribonucleoprotein PTB-binding 1 OS=Mus musculus GN=Raver1 PE=1 SV=2	1.000	0.910	0.955	0.386	0.415	0.541
Bifunctional purine biosynthesis protein PURH OS=Mus musculus GN=Atic PE=1 SV=2	0.987	1.001	0.994	1.430	1.549	1.540
Sorting nexin-2 OS=Mus musculus GN=Snx2 PE=1 SV=2	1.000	0.827	0.913	1.774	1.549	2.069
Beta-catenin-like protein 1 OS=Mus musculus GN=Ctnnbl1 PE=1 SV=1	1.000	0.979	0.989	0.916	1.309	1.273
N(G),N(G)-dimethylarginine dimethylaminohydrolase 1 OS=Mus musculus GN=Ddah1 PE=1 SV=3	0.875	0.814	0.844	0.902	1.068	1.038
Peptidyl-prolyl cis-trans isomerase NIMA-interacting 4 OS=Mus musculus GN=Pin4 PE=1 SV=1	0.956	0.938	0.947	1.930	2.307	2.158
RNA-binding protein 8A OS=Mus musculus GN=Rbm8a PE=1 SV=3	1.008	0.944	0.976	1.388	1.431	1.148
Zinc finger CCHC domain-containing protein 10 OS=Mus musculus GN=Zcchc10 PE=2 SV=1	1.000	1.034	1.017	1.438	0.906	1.605
26S proteasome non-ATPase regulatory subunit 8 OS=Mus musculus GN=Psmd8 PE=1 SV=2	1.000	0.952	0.976	1.187	0.943	1.518
Protein LBH OS=Mus musculus GN=Lbh PE=1 SV=1	1.000	0.887	0.944	1.135	0.744	0.836
Mesencephalic astrocyte-derived neurotrophic factor OS=Mus musculus GN=Manf PE=1 SV=1	1.006	1.064	1.035	1.065	1.046	1.033

**Calcyclin-binding protein OS=Mus musculus GN=Cacybp PE=1 SV=1**	1.029	1.056	1.042	1.641	2.046	1.696
**Interleukin enhancer-binding factor 2 OS=Mus musculus GN=Ilf2 PE=1 SV=1**	0.885	0.884	0.884	1.404	1.490	1.405
**Probable tRNA(His) guanylyltransferase OS=Mus musculus GN=Thg1l PE=1 SV=1**	1.000	2.161	1.581	1.782	0.798	1.068
**Plasminogen activator inhibitor 1 RNA-binding protein OS=Mus musculus GN=Serbp1 PE=1 SV=2**	1.111	1.051	1.081	1.753	2.126	1.594
**Cartilage-associated protein OS=Mus musculus GN=Crtap PE=1 SV=3**	1.000	0.784	0.892	0.536	0.549	0.524
**Putative RNA-binding protein Luc7-like 1 OS=Mus musculus GN=Luc7l PE=1 SV=2**	1.000	1.113	1.056	0.758	0.669	0.794
**Renin receptor OS=Mus musculus GN=Atp6ap2 PE=1 SV=2**	1.000	0.955	0.978	0.945	1.060	1.071
**Phosphoacetylglucosamine mutase OS=Mus musculus GN=Pgm3 PE=1 SV=1**	1.001	0.969	0.985	0.923	1.218	1.089
**Tumor protein D54 OS=Mus musculus GN=Tpd52l2 PE=1 SV=1**	1.073	1.010	1.042	1.141	0.971	1.347
**COP9 signalosome complex subunit 7a OS=Mus musculus GN=Cops7a PE=1 SV=2**	1.000	0.908	0.954	0.961	0.989	1.059
**Obg-like ATPase 1 OS=Mus musculus GN=Ola1 PE=1 SV=1**	1.000	1.134	1.067	1.666	1.314	1.578
**NSFL1 cofactor p47 OS=Mus musculus GN=Nsfl1c PE=1 SV=1**	0.895	0.913	0.904	0.952	0.939	0.902
**Glycine--tRNA ligase OS=Mus musculus GN=Gars PE=1 SV=1**	1.000	0.946	0.973	1.182	1.275	1.230
**Vasorin OS=Mus musculus GN=Vasn PE=2 SV=2**	1.000	0.738	0.869	0.302	0.403	0.422
**Superkiller viralicidic activity 2-like 2 OS=Mus musculus GN=Skiv2l2 PE=1 SV=1**	1.000	0.949	0.974	1.067	0.956	1.162
**Citrate synthase, mitochondrial OS=Mus musculus GN=Cs PE=1 SV=1**	1.000	1.023	1.011	1.198	1.177	1.201
**40S ribosomal protein S19 OS=Mus musculus GN=Rps19 PE=1 SV=3**	1.000	0.854	0.927	1.051	1.650	1.128
**Ubiquitin-conjugating enzyme E2 variant 1 OS=Mus musculus GN=Ube2v1 PE=1 SV=1**	1.026	1.063	1.045	1.365	1.529	1.380
**Protein PBDC1 OS=Mus musculus GN=Pbdc1 PE=1 SV=1**	1.000	0.959	0.980	0.740	1.584	0.726
**Heterogeneous nuclear ribonucleoprotein M OS=Mus musculus GN=Hnrnpm PE=1 SV=3**	1.000	0.960	0.980	0.426	0.679	0.938
**Phosphoglucomutase-1 OS=Mus musculus GN=Pgm1 PE=1 SV=4**	0.887	0.982	0.935	1.214	1.308	1.175
**Arginine--tRNA ligase, cytoplasmic OS=Mus musculus GN=Rars PE=1 SV=2**	0.959	0.961	0.960	1.146	1.234	1.249
**Parathymosin OS=Mus musculus GN=Ptms PE=1 SV=3**	1.114	1.080	1.097	1.129	1.218	1.309
**Succinyl-CoA:3-ketoacid coenzyme A transferase 1, mitochondrial OS=Mus musculus GN=Oxct1 PE=1 SV=1**	1.000	1.368	1.184	1.475	0.737	0.824
**Phosphoribosyl pyrophosphate synthase-associated protein 1 OS=Mus musculus GN=Prpsap1 PE=1 SV=1**	1.000	1.300	1.150	1.126	1.402	2.284
**Dynein light chain 2, cytoplasmic OS=Mus musculus GN=Dynll2 PE=1 SV=1**	1.011	1.056	1.034	2.349	2.873	2.231
**Threonine--tRNA ligase, cytoplasmic OS=Mus musculus GN=Tars PE=1 SV=2**	1.056	1.037	1.046	1.201	1.431	1.284
**Protein LSM12 homolog OS=Mus musculus GN=Lsm12 PE=1 SV=1**	1.000	0.906	0.953	0.796	0.818	0.857
**NHP2-like protein 1 OS=Mus musculus GN=Snu13 PE=1 SV=4**	1.000	0.706	0.853	0.654	0.789	0.555
**Peptidyl-prolyl cis-trans isomerase-like 1 OS=Mus musculus GN=Ppil1 PE=1 SV=1**	1.000	1.027	1.014	1.137	0.962	1.019

**Leukocyte elastase inhibitor A OS=Mus musculus GN=Serpinb1a PE=1 SV=1**	1.000	1.123	1.062	0.952	0.800	1.044
**ES1 protein homolog, mitochondrial OS=Mus musculus GN=D10Jhu81e PE=1 SV=1**	1.000	1.029	1.015	1.746	1.075	1.752
**Cytosolic non-specific dipeptidase OS=Mus musculus GN=Cndp2 PE=1 SV=1**	1.025	1.146	1.085	1.351	1.382	1.325
**Tubulin-folding cofactor B OS=Mus musculus GN=Tbcb PE=1 SV=2**	1.000	0.888	0.944	0.860	0.760	1.095
**Proline-rich AKT1 substrate 1 OS=Mus musculus GN=Akt1s1 PE=1 SV=1**	1.000	0.885	0.943	0.534	0.970	0.720
**Golgi to ER traffic protein 4 homolog OS=Mus musculus GN=Get4 PE=1 SV=2**	1.000	0.828	0.914	0.842	0.816	0.720
**Protein SEC13 homolog OS=Mus musculus GN=Sec13 PE=1 SV=3**	1.000	1.114	1.057	0.675	0.763	0.679
**Cysteine and histidine-rich domain-containing protein 1 OS=Mus musculus GN=Chordc1 PE=1 SV=1**	0.985	0.996	0.990	1.184	1.282	1.323
**Endoplasmic reticulum resident protein 44 OS=Mus musculus GN=Erp44 PE=1 SV=1**	1.045	0.888	0.966	0.626	0.583	0.626
**Pre-mRNA-splicing factor SPF27 OS=Mus musculus GN=Bcas2 PE=1 SV=1**	1.000	1.089	1.044	1.948	1.167	2.059
**Polypeptide N-acetylgalactosaminyltransferase 15 OS=Mus musculus GN=Galnt15 PE=2 SV=1**	1.000	1.256	1.128	1.581	0.798	1.389
**Ubiquitin-related modifier 1 OS=Mus musculus GN=Urm1 PE=1 SV=1**	1.000	0.936	0.968	0.947	0.801	1.117
**Low molecular weight phosphotyrosine protein phosphatase OS=Mus musculus GN=Acp1 PE=1 SV=3**	1.060	1.448	1.254	1.837	1.822	1.748
**Splicing factor 3A subunit 3 OS=Mus musculus GN=Sf3a3 PE=1 SV=2**	1.000	0.884	0.942	1.005	1.035	1.108
**Cullin-5 OS=Mus musculus GN=Cul5 PE=1 SV=3**	1.000	1.085	1.043	1.230	1.210	1.430
**Protein transport protein Sec23B OS=Mus musculus GN=Sec23b PE=1 SV=1**	1.023	1.556	1.290	1.675	1.522	1.240
**Carbonic anhydrase 13 OS=Mus musculus GN=Ca13 PE=1 SV=1**	1.000	0.928	0.964	3.899	4.682	2.342
**Dr1-associated corepressor OS=Mus musculus GN=Drap1 PE=1 SV=3**	1.000	1.244	1.122	1.149	0.900	1.517
**Polyadenylate-binding protein-interacting protein 2 OS=Mus musculus GN=Paip2 PE=1 SV=1**	1.000	0.743	0.871	0.615	0.572	0.680
**Oxidative stress-responsive serine-rich protein 1 OS=Mus musculus GN=Oser1 PE=2 SV=1**	1.000	1.167	1.083	1.054	1.058	0.548
**Exosome complex component RRP43 OS=Mus musculus GN=Exosc8 PE=1 SV=1**	1.000	0.961	0.981	1.230	1.058	1.286
**Ribose-phosphate pyrophosphokinase 1 OS=Mus musculus GN=Prps1 PE=1 SV=4**	1.000	0.961	0.981	0.864	0.702	0.894
**Protein shisa-5 OS=Mus musculus GN=Shisa5 PE=1 SV=1**	1.000	0.996	0.998	0.860	0.683	0.825
**DNA-directed RNA polymerase II subunit RPB4 OS=Mus musculus GN=Polr2d PE=1 SV=2**	1.000	1.247	1.124	1.209	1.314	1.867
**LysM and putative peptidoglycan-binding domain-containing protein 2 OS=Mus musculus GN=Lysmd2 PE=1 SV=2**	1.000	0.772	0.886	0.440	0.722	0.450
**Nucleolar protein 7 OS=Mus musculus GN=Nol7 PE=1 SV=1**	1.000	0.959	0.980	2.070	1.972	1.936
**Inorganic pyrophosphatase OS=Mus musculus GN=Ppa1 PE=1 SV=1**	1.015	1.008	1.011	1.250	1.805	1.400
**60S ribosomal protein L37 OS=Mus musculus GN=Rpl37 PE=3 SV=3**	1.000	1.000	1.000	1.000	1.680	1.974
**DnaJ homolog subfamily B member 4 OS=Mus musculus GN=Dnajb4 PE=1 SV=1**	1.000	0.939	0.970	0.980	0.915	0.883
**DnaJ homolog subfamily C member 4 OS=Mus**	1.000	1.211	1.105	1.156	0.981	1.305

musculus GN=Dnajc4 PE=2 SV=2						
Inosine triphosphate pyrophosphatase OS=Mus musculus GN=Itpa PE=1 SV=2	1.131	1.008	1.070	1.288	1.293	1.258
Charged multivesicular body protein 4b OS=Mus musculus GN=Chmp4b PE=1 SV=2	1.000	0.796	0.898	0.520	0.524	0.794
Elongation factor 1-gamma OS=Mus musculus GN=Eef1g PE=1 SV=3	1.009	1.055	1.032	1.393	1.671	1.481
Oligoribonuclease, mitochondrial OS=Mus musculus GN=Rexo2 PE=1 SV=2	0.966	0.963	0.965	0.947	0.965	1.020
BolA-like protein 1 OS=Mus musculus GN=Bola1 PE=1 SV=1	0.963	1.077	1.020	0.747	0.664	0.824
Sorting nexin-5 OS=Mus musculus GN=Snx5 PE=1 SV=1	1.000	1.723	1.362	2.073	1.755	1.661
26S proteasome non-ATPase regulatory subunit 12 OS=Mus musculus GN=Psmd12 PE=1 SV=4	1.094	1.071	1.083	1.212	1.410	1.138
Coiled-coil domain-containing protein 124 OS=Mus musculus GN=Ccdc124 PE=1 SV=1	1.008	1.158	1.083	2.486	2.693	2.863
EF-hand domain-containing protein D2 OS=Mus musculus GN=Efhd2 PE=1 SV=1	1.000	1.073	1.036	1.160	1.445	1.022
Magnesium-dependent phosphatase 1 OS=Mus musculus GN=Mdp1 PE=1 SV=1	0.808	0.755	0.781	1.158	0.917	0.813
m7GpppX diphosphatase OS=Mus musculus GN=Dcps PE=1 SV=1	1.000	0.924	0.962	1.266	1.408	1.320
Guanine nucleotide-binding protein G(I)/G(S)/G(O) subunit gamma-12 OS=Mus musculus GN=Gng12 PE=1 SV=3	1.000	0.809	0.904	0.496	0.688	0.471
U4/U6 small nuclear ribonucleoprotein Prp4 OS=Mus musculus GN=Prpf4 PE=1 SV=1	1.000	0.960	0.980	1.025	1.149	0.805
Calponin-3 OS=Mus musculus GN=Cnn3 PE=1 SV=1	1.000	1.056	1.028	1.236	1.030	1.242
39S ribosomal protein L12, mitochondrial OS=Mus musculus GN=Mrpl12 PE=1 SV=2	1.000	1.017	1.008	1.559	1.016	1.561
cAMP-dependent protein kinase type I-alpha regulatory subunit OS=Mus musculus GN=Prkar1a PE=1 SV=3	1.000	1.276	1.138	1.134	1.154	0.956
Alpha-aminoadipic semialdehyde dehydrogenase OS=Mus musculus GN=Aldh7a1 PE=1 SV=4	1.000	0.803	0.902	0.703	0.488	0.756
AP-2 complex subunit beta OS=Mus musculus GN=Ap2b1 PE=1 SV=1	1.055	1.000	1.027	1.045	1.053	1.243
Perilipin-3 OS=Mus musculus GN=Plin3 PE=1 SV=1	1.016	1.073	1.044	0.955	0.874	0.972
Tax1-binding protein 3 OS=Mus musculus GN=Tax1bp3 PE=1 SV=1	1.000	0.856	0.928	0.629	0.490	0.777
Phosphoglycerate mutase 1 OS=Mus musculus GN=Pgam1 PE=1 SV=3	1.080	1.085	1.083	2.116	2.726	1.920
UMP-CMP kinase OS=Mus musculus GN=Cmpk1 PE=1 SV=1	0.996	1.066	1.031	0.958	1.246	1.225
Protein-associating with the carboxyl-terminal domain of ezrin OS=Mus musculus GN=Scyl3 PE=1 SV=3	1.000	1.366	1.183	1.337	2.028	1.382
5'-3' exoribonuclease 2 OS=Mus musculus GN=Xrn2 PE=1 SV=1	1.020	1.804	1.412	2.409	1.536	2.118
Protein phosphatase 1 regulatory subunit 12A OS=Mus musculus GN=Ppp1r12a PE=1 SV=2	1.000	0.998	0.999	0.962	0.914	1.043
Eukaryotic translation initiation factor 3 subunit K OS=Mus musculus GN=Eif3k PE=1 SV=1	1.057	1.019	1.038	0.796	0.863	0.885
Pyrroline-5-carboxylate reductase 3 OS=Mus musculus GN=Pycrl PE=1 SV=2	1.000	0.862	0.931	2.280	1.215	1.697
Mitochondrial import receptor subunit TOM20 homolog OS=Mus musculus GN=Tomm20 PE=1 SV=1	1.000	1.065	1.032	0.821	0.998	0.921
6-phosphogluconate dehydrogenase, decarboxylating OS=Mus musculus GN=Pgd PE=1	1.030	0.981	1.005	1.247	1.263	1.222

SV=3						
Multifunctional methyltransferase subunit TRM112-like protein OS=Mus musculus GN=Trmt112 PE=1 SV=1	1.000	0.914	0.957	1.211	0.870	1.355
Eukaryotic translation initiation factor 3 subunit F OS=Mus musculus GN=Eif3f PE=1 SV=2	1.086	0.960	1.023	0.921	0.946	0.823
Multifunctional protein ADE2 OS=Mus musculus GN=Paics PE=1 SV=4	1.038	0.927	0.983	1.000	1.160	1.065
NADH-cytochrome b5 reductase 3 OS=Mus musculus GN=Cyb5r3 PE=1 SV=3	1.000	0.689	0.844	0.542	0.564	0.417
Cysteine-rich protein 2 OS=Mus musculus GN=Crip2 PE=1 SV=1	1.000	1.257	1.128	0.675	0.569	0.677
Electron transfer flavoprotein subunit beta OS=Mus musculus GN=Etfb PE=1 SV=3	1.000	0.752	0.876	0.417	0.430	0.588
Retinoic acid receptor responder protein 2 OS=Mus musculus GN=Rarres2 PE=1 SV=1	1.000	1.052	1.026	1.048	1.264	1.171
D-tyrosyl-tRNA(Tyr) deacylase 1 OS=Mus musculus GN=Dtd1 PE=1 SV=2	1.000	0.843	0.922	1.641	0.945	0.885
Twisted gastrulation protein homolog 1 OS=Mus musculus GN=Twsg1 PE=1 SV=1	1.000	0.845	0.923	0.581	0.621	0.846
Ankycorbin OS=Mus musculus GN=Rai14 PE=1 SV=1	1.000	0.896	0.948	0.710	0.929	1.074
Alpha-parvin OS=Mus musculus GN=Parva PE=1 SV=1	1.000	0.888	0.944	1.150	0.982	0.994
Calsyntenin-1 OS=Mus musculus GN=Clstn1 PE=1 SV=1	1.013	0.961	0.987	0.641	0.634	0.712
Importin-7 OS=Mus musculus GN=Ipo7 PE=1 SV=2	1.058	1.029	1.044	1.088	1.217	1.240
Regulator of nonsense transcripts 1 OS=Mus musculus GN=Upf1 PE=1 SV=2	1.000	0.927	0.964	1.250	1.127	0.969
Follistatin-related protein 3 OS=Mus musculus GN=Fstl3 PE=1 SV=1	1.000	1.036	1.018	0.771	0.523	0.732
Vacuolar protein sorting-associated protein 35 OS=Mus musculus GN=Vps35 PE=1 SV=1	1.001	1.101	1.051	1.255	1.245	1.521
Major vault protein OS=Mus musculus GN=Mvp PE=1 SV=4	1.000	1.104	1.052	0.706	0.688	0.888
Protein SET OS=Mus musculus GN=Set PE=1 SV=1	1.045	0.924	0.985	1.959	2.334	1.892
Allograft inflammatory factor 1-like OS=Mus musculus GN=Aif1l PE=1 SV=1	1.053	0.908	0.980	0.845	1.119	0.991
Syntaxin-12 OS=Mus musculus GN=Stx12 PE=1 SV=1	1.000	1.200	1.100	1.305	0.697	0.805
Brain-specific serine protease 4 OS=Mus musculus GN=Prss22 PE=2 SV=1	1.000	0.513	0.756	0.728	1.154	1.144
Cysteine--tRNA ligase, cytoplasmic OS=Mus musculus GN=Cars PE=1 SV=2	0.865	0.930	0.898	1.183	1.144	1.218
LDLR chaperone MESD OS=Mus musculus GN=Mesdc2 PE=1 SV=1	1.000	1.180	1.090	1.328	1.352	1.268
LIM domain and actin-binding protein 1 OS=Mus musculus GN=Lima1 PE=1 SV=3	1.003	1.005	1.004	1.374	1.515	1.496
Striatin-3 OS=Mus musculus GN=Strn3 PE=1 SV=1	1.000	0.819	0.910	0.915	0.898	1.117
Exportin-2 OS=Mus musculus GN=Cse1l PE=1 SV=1	1.035	0.959	0.997	1.342	1.481	1.207
15 kDa selenoprotein OS=Mus musculus GN=Sep15 PE=1 SV=3	1.000	0.800	0.900	0.963	0.996	0.814
E3 SUMO-protein ligase RanBP2 OS=Mus musculus GN=Ranbp2 PE=1 SV=2	0.049	3.002	1.525	12.646	9.754	13.326
Exostosin-like 2 OS=Mus musculus GN=Extl2 PE=1 SV=1	1.000	1.153	1.076	0.843	0.914	0.743
H/ACA ribonucleoprotein complex subunit 4 OS=Mus musculus GN=Dkc1 PE=1 SV=4	1.120	0.944	1.032	1.053	1.342	0.980
Palladin OS=Mus musculus GN=Palld PE=1 SV=2	1.000	1.034	1.017	1.349	1.374	1.501
Matrix metalloproteinase-19 OS=Mus musculus GN=Mmp19 PE=2 SV=1	1.000	1.024	1.012	0.904	1.192	1.417

**Tropomodulin-3 OS=Mus musculus GN=Tmod3 PE=1 SV=1**	1.037	1.308	1.173	1.507	1.470	1.740
**Insulin-degrading enzyme OS=Mus musculus GN=Ide PE=1 SV=1**	1.000	1.045	1.023	1.091	1.209	1.092
**Ragulator complex protein LAMTOR2 OS=Mus musculus GN=Lamtor2 PE=1 SV=1**	1.000	1.909	1.454	0.639	2.969	4.472
**Cytoplasmic dynein 1 heavy chain 1 OS=Mus musculus GN=Dync1h1 PE=1 SV=2**	1.094	1.041	1.067	1.010	0.919	1.148
**Diphosphoinositol polyphosphate phosphohydrolase 1 OS=Mus musculus GN=Nudt3 PE=1 SV=1**	1.000	0.889	0.945	0.949	0.822	0.923
**Ribosyldihydronicotinamide dehydrogenase [quinone] OS=Mus musculus GN=Nqo2 PE=1 SV=3**	1.000	0.823	0.912	1.157	0.823	0.995
**Alpha-actinin-2 OS=Mus musculus GN=Actn2 PE=1 SV=2**	1.000	1.056	1.028	0.946	0.751	1.001
**Protein arginine N-methyltransferase 1 OS=Mus musculus GN=Prmt1 PE=1 SV=1**	1.000	1.017	1.008	1.300	1.706	1.199
**Coatomer subunit beta OS=Mus musculus GN=Copb1 PE=1 SV=1**	0.945	1.024	0.984	1.165	1.499	1.185
**Alcohol dehydrogenase [NADP(+)] OS=Mus musculus GN=Akr1a1 PE=1 SV=3**	1.086	1.099	1.092	1.310	1.562	1.356
**Nucleolar RNA helicase 2 OS=Mus musculus GN=Ddx21 PE=1 SV=3**	1.000	0.867	0.933	1.115	1.835	1.188
**Transcription and mRNA export factor ENY2 OS=Mus musculus GN=Eny2 PE=1 SV=1**	1.031	0.828	0.930	0.769	0.987	0.792
**RILP-like protein 1 OS=Mus musculus GN=Rilpl1 PE=1 SV=1**	1.000	1.113	1.057	1.351	0.761	1.121
**Bifunctional lysine-specific demethylase and histidyl-hydroxylase NO66 OS=Mus musculus GN=No66 PE=1 SV=2**	1.000	1.043	1.021	1.011	1.223	0.979
**Ribonuclease 4 OS=Mus musculus GN=Rnase4 PE=1 SV=1**	1.124	1.052	1.088	1.214	0.876	1.027
**Phosphorylated adapter RNA export protein OS=Mus musculus GN=Phax PE=1 SV=1**	1.000	0.828	0.914	0.907	0.705	1.346
**SH3 domain-binding glutamic acid-rich-like protein OS=Mus musculus GN=Sh3bgrl PE=1 SV=1**	0.915	0.969	0.942	1.130	1.458	1.339
**Endophilin-B1 OS=Mus musculus GN=Sh3glb1 PE=1 SV=1**	1.000	0.773	0.887	0.323	0.312	0.280
**UPF0160 protein MYG1, mitochondrial OS=Mus musculus GN=Myg1 PE=1 SV=1**	1.000	1.151	1.076	1.322	1.926	1.705
**Ubiquitin carboxyl-terminal hydrolase isozyme L3 OS=Mus musculus GN=Uchl3 PE=1 SV=2**	0.972	1.130	1.051	3.560	3.783	3.106
**Ras GTPase-activating-like protein IQGAP1 OS=Mus musculus GN=Iqgap1 PE=1 SV=2**	1.081	1.105	1.093	1.282	1.239	1.274
**DNA polymerase epsilon subunit 3 OS=Mus musculus GN=Pole3 PE=1 SV=1**	1.000	0.737	0.869	0.446	0.426	0.848
**Hypoxia up-regulated protein 1 OS=Mus musculus GN=Hyou1 PE=1 SV=1**	1.074	1.060	1.067	0.981	0.866	1.120
**High mobility group nucleosome-binding domain-containing protein 5 OS=Mus musculus GN=Hmgn5 PE=1 SV=2**	1.000	0.984	0.992	0.606	0.479	0.820
**MAGUK p55 subfamily member 6 OS=Mus musculus GN=Mpp6 PE=1 SV=1**	1.000	0.970	0.985	0.900	1.182	1.414
**Sacsin OS=Mus musculus GN=Sacs PE=1 SV=2**	1.000	0.937	0.968	0.359	0.322	0.036
**BAG family molecular chaperone regulator 3 OS=Mus musculus GN=Bag3 PE=1 SV=2**	1.291	0.951	1.121	0.944	1.183	1.151
**Cullin-3 OS=Mus musculus GN=Cul3 PE=1 SV=1**	1.000	1.051	1.025	0.739	0.843	0.807
**Actin-related protein 2/3 complex subunit 3 OS=Mus musculus GN=Arpc3 PE=1 SV=3**	1.000	0.894	0.947	1.045	0.932	0.855
**Ubiquitin carboxyl-terminal hydrolase 14 OS=Mus musculus GN=Usp14 PE=1 SV=3**	0.963	0.914	0.938	1.163	1.277	1.246

**BUB3-interacting and GLEBS motif-containing protein ZNF207 OS=Mus musculus GN=Znf207 PE=1 SV=1**	0.933	0.854	0.894	0.999	0.897	1.043
**Endothelial differentiation-related factor 1 OS=Mus musculus GN=Edf1 PE=1 SV=1**	1.000	1.124	1.062	1.505	1.483	1.866
**Thioredoxin reductase 1, cytoplasmic OS=Mus musculus GN=Txnrd1 PE=1 SV=3**	0.937	1.000	0.968	1.397	1.412	1.282
**Glutaredoxin-1 OS=Mus musculus GN=Glrx PE=1 SV=3**	1.000	1.140	1.070	1.523	1.601	1.585
**Transforming protein RhoA OS=Mus musculus GN=Rhoa PE=1 SV=1**	1.030	1.044	1.037	1.092	1.018	1.058
**Proteasome subunit alpha type-6 OS=Mus musculus GN=Psma6 PE=1 SV=1**	1.090	0.991	1.041	1.183	1.603	1.190
**Dickkopf-related protein 3 OS=Mus musculus GN=Dkk3 PE=2 SV=1**	0.908	0.887	0.898	0.475	0.476	0.562
**Prolyl endopeptidase OS=Mus musculus GN=Prep PE=1 SV=1**	1.017	0.984	1.000	1.221	1.366	1.166
**Peptidyl-prolyl cis-trans isomerase NIMA-interacting 1 OS=Mus musculus GN=Pin1 PE=1 SV=1**	1.000	0.856	0.928	1.207	1.484	1.097
**Protein-tyrosine kinase 2-beta OS=Mus musculus GN=Ptk2b PE=1 SV=2**	1.000	0.945	0.973	1.191	0.747	1.260
**Alpha-N-acetylgalactosaminidase OS=Mus musculus GN=Naga PE=1 SV=2**	1.000	1.145	1.073	1.764	0.998	1.289
**Short transient receptor potential channel 5 OS=Mus musculus GN=Trpc5 PE=1 SV=2**	1.000	1.818	1.409	1.520	0.977	1.933
**U6 snRNA-associated Sm-like protein LSm4 OS=Mus musculus GN=Lsm4 PE=1 SV=1**	1.000	0.957	0.978	1.274	1.624	1.698
**b(0,+)-type amino acid transporter 1 OS=Mus musculus GN=Slc7a9 PE=1 SV=1**	1.000	0.955	0.977	0.833	0.590	0.586
**LIM domain-containing protein 1 OS=Mus musculus GN=Limd1 PE=1 SV=2**	1.000	0.716	0.858	0.576	0.780	0.383
**Plectin OS=Mus musculus GN=Plec PE=1 SV=3**	0.988	1.032	1.010	1.249	1.259	1.288
**Drebrin OS=Mus musculus GN=Dbn1 PE=1 SV=4**	1.000	0.997	0.999	1.077	0.906	1.171
**Protein canopy homolog 2 OS=Mus musculus GN=Cnpy2 PE=1 SV=1**	1.052	1.078	1.065	1.121	1.019	1.039
**Microtubule-actin cross-linking factor 1 OS=Mus musculus GN=Macf1 PE=1 SV=2**	0.940	1.014	0.977	1.155	0.930	1.003
**N-alpha-acetyltransferase 10 OS=Mus musculus GN=Naa10 PE=1 SV=1**	1.000	1.044	1.022	1.345	1.296	1.147
**Chloride intracellular channel protein 4 OS=Mus musculus GN=Clic4 PE=1 SV=3**	1.036	1.004	1.020	1.181	1.322	1.310
**DnaJ homolog subfamily A member 2 OS=Mus musculus GN=Dnaja2 PE=1 SV=1**	0.951	0.791	0.871	0.583	0.714	1.017
**Toll-interacting protein OS=Mus musculus GN=Tollip PE=1 SV=1**	1.000	0.745	0.873	0.445	0.399	0.873
**Vascular non-inflammatory molecule 3 OS=Mus musculus GN=Vnn3 PE=1 SV=3**	1.000	1.146	1.073	1.534	0.942	1.692
**Vacuolar protein sorting-associated protein 29 OS=Mus musculus GN=Vps29 PE=1 SV=1**	1.000	1.130	1.065	1.628	1.662	1.752
**Actin-related protein 10 OS=Mus musculus GN=Actr10 PE=1 SV=2**	1.000	1.015	1.008	0.895	1.216	1.138
**Eukaryotic translation initiation factor 3 subunit I OS=Mus musculus GN=Eif3i PE=1 SV=1**	1.000	1.010	1.005	1.370	1.317	1.455
**Coatomer subunit gamma-1 OS=Mus musculus GN=Copg1 PE=1 SV=1**	1.143	1.121	1.132	1.096	1.341	1.283
**Glypican-1 OS=Mus musculus GN=Gpc1 PE=1 SV=1**	1.000	1.153	1.076	0.927	0.759	1.134
**Ubiquilin-2 OS=Mus musculus GN=Ubqln2 PE=1 SV=2**	1.000	0.952	0.976	1.169	0.865	1.259
**Afadin OS=Mus musculus GN=Mllt4 PE=1 SV=3**	1.000	0.849	0.925	0.708	0.831	0.917
**Unconventional myosin-XV OS=Mus musculus**	1.000	1.010	1.005	1.460	0.967	1.174

GN=Myo15a PE=1 SV=2						
Cathepsin F OS=Mus musculus GN=Ctsf PE=1 SV=1	1.000	1.216	1.108	0.888	0.454	0.336
Four and a half LIM domains protein 3 OS=Mus musculus GN=Fhl3 PE=1 SV=2	1.000	1.323	1.161	2.548	2.840	4.259
Glycogenin-1 OS=Mus musculus GN=Gyg1 PE=1 SV=3	1.000	0.800	0.900	1.095	0.762	0.568
Glypican-6 OS=Mus musculus GN=Gpc6 PE=1 SV=1	1.000	1.030	1.015	0.802	0.622	0.553
Procollagen-lysine,2-oxoglutarate 5-dioxygenase 2 OS=Mus musculus GN=Plod2 PE=1 SV=2	1.000	0.818	0.909	0.956	0.517	0.595
Procollagen-lysine,2-oxoglutarate 5-dioxygenase 3 OS=Mus musculus GN=Plod3 PE=1 SV=1	1.000	0.946	0.973	0.709	0.756	0.819
Procollagen-lysine,2-oxoglutarate 5-dioxygenase 1 OS=Mus musculus GN=Plod1 PE=1 SV=1	0.944	0.914	0.929	0.873	0.741	0.749
Cartilage oligomeric matrix protein OS=Mus musculus GN=Comp PE=1 SV=2	1.000	0.956	0.978	1.249	0.732	1.387
S-formylglutathione hydrolase OS=Mus musculus GN=Esd PE=1 SV=1	0.758	0.985	0.871	1.300	1.556	1.312
Small acidic protein OS=Mus musculus GN=Smap PE=1 SV=1	1.004	0.967	0.985	1.534	1.870	1.338
Destrin OS=Mus musculus GN=Dstn PE=1 SV=3	1.047	1.020	1.033	1.394	1.660	1.493
Prostaglandin E synthase 3 OS=Mus musculus GN=Ptges3 PE=1 SV=1	0.896	0.858	0.877	0.960	1.100	0.923
Serine protease HTRA1 OS=Mus musculus GN=Htra1 PE=1 SV=2	1.029	0.889	0.959	1.142	0.758	0.938
Pendrin OS=Mus musculus GN=Slc26a4 PE=1 SV=1	1.000	0.993	0.996	1.225	1.187	1.138
Slit homolog 2 protein OS=Mus musculus GN=Slit2 PE=2 SV=2	1.000	0.937	0.969	0.731	0.700	0.974
Pre-mRNA-processing factor 40 homolog A OS=Mus musculus GN=Prpf40a PE=1 SV=1	1.000	1.020	1.010	1.348	1.035	1.434
Ectonucleotide pyrophosphatase/phosphodiesterase family member 2 OS=Mus musculus GN=Enpp2 PE=1 SV=3	1.069	1.052	1.060	1.466	1.375	1.313
Proteasome subunit alpha type-4 OS=Mus musculus GN=Psma4 PE=1 SV=1	1.102	1.142	1.122	1.909	2.004	1.593
Proteasome subunit beta type-3 OS=Mus musculus GN=Psmb3 PE=1 SV=1	1.074	1.055	1.065	1.521	1.772	1.381
Proteasome subunit beta type-2 OS=Mus musculus GN=Psmb2 PE=1 SV=1	0.988	0.988	0.988	1.240	1.480	1.314
Proteasome subunit alpha type-1 OS=Mus musculus GN=Psma1 PE=1 SV=1	0.931	1.119	1.025	2.234	2.459	2.164
Transmembrane protein 176B OS=Mus musculus GN=Tmem176b PE=1 SV=1	1.000	1.046	1.023	0.642	1.692	1.522
V-type proton ATPase subunit S1 OS=Mus musculus GN=Atp6ap1 PE=1 SV=1	0.952	0.925	0.938	0.979	0.744	0.975
SUMO-activating enzyme subunit 1 OS=Mus musculus GN=Sae1 PE=1 SV=1	0.962	0.897	0.929	1.313	1.089	1.280
Insulin receptor-related protein OS=Mus musculus GN=Insrr PE=1 SV=2	1.000	1.012	1.006	1.242	1.611	1.429
RuvB-like 2 OS=Mus musculus GN=Ruvbl2 PE=1 SV=3	1.000	0.952	0.976	1.095	1.049	1.070
Adenylate kinase 2, mitochondrial OS=Mus musculus GN=Ak2 PE=1 SV=5	1.006	1.060	1.033	1.786	1.613	1.591
A-kinase anchor protein 12 OS=Mus musculus GN=Akap12 PE=1 SV=1	1.000	1.079	1.040	0.542	0.409	0.502
Cadherin-13 OS=Mus musculus GN=Cdh13 PE=1 SV=2	0.967	0.866	0.917	1.105	1.069	1.166
S-phase kinase-associated protein 1 OS=Mus musculus GN=Skp1 PE=1 SV=3	1.017	0.928	0.972	1.317	1.356	1.266
Cullin-1 OS=Mus musculus GN=Cul1 PE=1 SV=1	1.000	1.003	1.001	1.053	0.966	1.210

**Prefoldin subunit 5 OS=Mus musculus GN=Pfdn5 PE=1 SV=1**	0.762	0.848	0.805	1.439	1.304	1.326
**Programmed cell death 6-interacting protein OS=Mus musculus GN=Pdcd6ip PE=1 SV=3**	0.956	0.940	0.948	1.288	1.194	1.244
**Phenylalanine--tRNA ligase beta subunit OS=Mus musculus GN=Farsb PE=1 SV=2**	1.000	1.043	1.022	1.595	2.959	3.126
**STIP1 homology and U box-containing protein 1 OS=Mus musculus GN=Stub1 PE=1 SV=1**	1.000	0.872	0.936	0.873	0.964	0.777
**Eukaryotic translation initiation factor 4H OS=Mus musculus GN=Eif4h PE=1 SV=3**	1.000	1.022	1.011	0.806	0.925	0.765
**Coronin-1B OS=Mus musculus GN=Coro1b PE=1 SV=1**	1.000	0.931	0.965	0.832	0.725	0.968
**Coronin-1C OS=Mus musculus GN=Coro1c PE=1 SV=2**	1.238	1.091	1.164	1.077	1.125	1.042
**Ubiquitin carboxyl-terminal hydrolase isozyme L5 OS=Mus musculus GN=Uchl5 PE=1 SV=2**	1.000	0.733	0.867	0.607	0.515	0.788
**Cathepsin Z OS=Mus musculus GN=Ctsz PE=1 SV=1**	1.000	1.014	1.007	0.751	0.655	0.750
**Protein FAM50A OS=Mus musculus GN=Fam50a PE=1 SV=1**	0.954	0.902	0.928	1.092	1.305	1.370
**Actin-related protein 2/3 complex subunit 1B OS=Mus musculus GN=Arpc1b PE=1 SV=4**	1.000	0.963	0.981	1.293	1.215	1.777
**Acid ceramidase OS=Mus musculus GN=Asah1 PE=1 SV=1**	1.000	1.073	1.037	0.854	0.735	0.960
**Vesicle-associated membrane protein-associated protein A OS=Mus musculus GN=Vapa PE=1 SV=2**	0.969	0.945	0.957	2.578	2.847	2.723
**Sorting nexin-1 OS=Mus musculus GN=Snx1 PE=1 SV=1**	1.000	0.989	0.995	1.150	1.009	1.282
**Band 4.1-like protein 3 OS=Mus musculus GN=Epb41l3 PE=1 SV=1**	1.000	1.011	1.006	1.097	1.131	1.149
**Mitochondrial import inner membrane translocase subunit Tim8 A OS=Mus musculus GN=Timm8a1 PE=1 SV=1**	1.000	0.970	0.985	1.757	1.391	1.555
**Mitotic checkpoint protein BUB3 OS=Mus musculus GN=Bub3 PE=1 SV=2**	0.987	1.041	1.014	1.342	1.289	1.582
**Transgelin-2 OS=Mus musculus GN=Tagln2 PE=1 SV=4**	1.020	1.092	1.056	1.239	1.258	1.240
**Slit homolog 3 protein OS=Mus musculus GN=Slit3 PE=2 SV=2**	1.000	0.993	0.996	1.911	0.703	0.956
**Chloride anion exchanger OS=Mus musculus GN=Slc26a3 PE=1 SV=1**	1.000	0.737	0.869	0.711	0.511	0.629
**Protein kinase C and casein kinase substrate in neurons protein 2 OS=Mus musculus GN=Pacsin2 PE=1 SV=1**	0.998	1.362	1.180	1.666	1.849	2.097
**Forkhead box protein O4 OS=Mus musculus GN=Foxo4 PE=1 SV=1**	1.000	1.119	1.059	1.021	0.761	1.115
**Fibulin-5 OS=Mus musculus GN=Fbln5 PE=1 SV=1**	0.946	0.956	0.951	0.564	0.501	0.643
**26S proteasome non-ATPase regulatory subunit 13 OS=Mus musculus GN=Psmd13 PE=1 SV=1**	0.966	1.044	1.005	1.206	1.008	1.159
**Carboxypeptidase Q OS=Mus musculus GN=Cpq PE=1 SV=1**	0.992	0.933	0.963	0.781	0.667	0.882
**EGF-containing fibulin-like extracellular matrix protein 2 OS=Mus musculus GN=Efemp2 PE=2 SV=1**	0.928	0.875	0.902	0.594	0.586	0.686
**EH domain-containing protein 1 OS=Mus musculus GN=Ehd1 PE=1 SV=1**	0.944	1.052	0.998	1.219	1.404	1.258
**Methylthioribulose-1-phosphate dehydratase OS=Mus musculus GN=Apip PE=1 SV=1**	1.000	0.782	0.891	1.209	1.021	0.959
**CX3C chemokine receptor 1 OS=Mus musculus GN=Cx3cr1 PE=1 SV=1**	1.000	0.789	0.894	0.213	0.177	0.236
**Guanylate-binding protein 1 OS=Mus musculus GN=Gbp2 PE=1 SV=1**	0.962	0.934	0.948	0.690	0.640	0.892

**Epididymal secretory protein E1 OS=Mus musculus GN=Npc2 PE=1 SV=1**	0.952	1.026	0.989	0.932	0.878	0.940
**Eukaryotic translation initiation factor 2 subunit 3, X-linked OS=Mus musculus GN=Eif2s3x PE=1 SV=2**	1.016	1.134	1.075	2.170	2.390	2.336
**Paralemmin-1 OS=Mus musculus GN=Palm PE=1 SV=1**	1.000	0.818	0.909	0.619	0.487	0.736
**Twinfilin-2 OS=Mus musculus GN=Twf2 PE=1 SV=1**	1.000	1.090	1.045	1.123	1.289	2.207
**Apoptosis-inducing factor 1, mitochondrial OS=Mus musculus GN=Aifm1 PE=1 SV=1**	1.000	0.918	0.959	0.859	0.598	0.893
**Delta-1-pyrroline-5-carboxylate synthase OS=Mus musculus GN=Aldh18a1 PE=1 SV=2**	1.000	1.223	1.111	1.562	1.064	1.821
**C-C motif chemokine 8 OS=Mus musculus GN=Ccl8 PE=3 SV=1**	1.000	1.006	1.003	2.008	2.813	1.760
**Heterogeneous nuclear ribonucleoprotein D-like OS=Mus musculus GN=Hnrnpdl PE=1 SV=1**	1.053	0.968	1.010	1.083	1.277	1.133
**Lysyl oxidase homolog 3 OS=Mus musculus GN=Loxl3 PE=2 SV=2**	1.000	1.013	1.007	0.807	0.934	0.950
**Mitotic spindle assembly checkpoint protein MAD2A OS=Mus musculus GN=Mad2l1 PE=1 SV=2**	1.000	0.835	0.917	0.713	0.664	0.746
**Eukaryotic translation initiation factor 3 subunit G OS=Mus musculus GN=Eif3g PE=1 SV=2**	1.053	1.060	1.057	1.474	1.484	1.579
**SUMO-activating enzyme subunit 2 OS=Mus musculus GN=Uba2 PE=1 SV=1**	1.104	1.063	1.084	1.202	1.269	1.348
**V-type proton ATPase subunit C 1 OS=Mus musculus GN=Atp6v1c1 PE=1 SV=4**	1.000	1.050	1.025	1.167	1.487	1.783
**E3 ubiquitin-protein ligase ARIH1 OS=Mus musculus GN=Arih1 PE=1 SV=3**	1.000	0.809	0.905	0.517	0.799	0.707
**Spliceosome RNA helicase Ddx39b OS=Mus musculus GN=Ddx39b PE=1 SV=1**	1.000	1.036	1.018	1.618	1.430	1.628
**Angiopoietin-related protein 4 OS=Mus musculus GN=Angptl4 PE=2 SV=1**	1.000	1.306	1.153	1.423	1.296	1.605
**Chloride intracellular channel protein 1 OS=Mus musculus GN=Clic1 PE=1 SV=3**	1.016	1.038	1.027	1.595	1.574	1.651
**Valine--tRNA ligase OS=Mus musculus GN=Vars PE=1 SV=1**	1.058	1.115	1.087	1.335	1.474	1.497
**Large proline-rich protein BAG6 OS=Mus musculus GN=Bag6 PE=1 SV=1**	0.966	0.967	0.966	1.506	1.521	1.484
**Thrombospondin-4 OS=Mus musculus GN=Thbs4 PE=1 SV=1**	1.000	0.931	0.965	0.959	0.861	0.769
**Interleukin enhancer-binding factor 3 OS=Mus musculus GN=Ilf3 PE=1 SV=2**	1.066	0.998	1.032	1.107	1.043	1.064
**General vesicular transport factor p115 OS=Mus musculus GN=Uso1 PE=1 SV=2**	1.000	0.991	0.996	1.205	1.424	1.446
**Serine-threonine kinase receptor-associated protein OS=Mus musculus GN=Strap PE=1 SV=2**	1.000	0.945	0.973	1.271	1.326	1.302
**Heterogeneous nuclear ribonucleoproteins C1/C2 OS=Mus musculus GN=Hnrnpc PE=1 SV=1**	0.992	0.909	0.950	1.108	1.532	1.195
**Peptidyl-prolyl cis-trans isomerase FKBP9 OS=Mus musculus GN=Fkbp9 PE=1 SV=1**	1.000	0.813	0.906	0.509	0.490	0.541
**Ubiquitin-conjugating enzyme E2 A OS=Mus musculus GN=Ube2a PE=1 SV=1**	1.000	1.699	1.350	1.652	1.197	0.627
**Methyl-CpG-binding domain protein 3 OS=Mus musculus GN=Mbd3 PE=1 SV=1**	1.000	1.477	1.238	1.120	1.142	3.064
**Keratin, type I cytoskeletal 16 OS=Mus musculus GN=Krt16 PE=1 SV=3**	1.030	0.953	0.991	0.707	0.740	0.958
**Multiple inositol polyphosphate phosphatase 1 OS=Mus musculus GN=Minpp1 PE=1 SV=3**	0.946	0.922	0.934	0.957	1.036	1.377
**Phosphomannomutase 2 OS=Mus musculus GN=Pmm2 PE=1 SV=1**	1.000	0.902	0.951	1.697	1.401	1.732

**Septin-5 OS=Mus musculus GN=Sept5 PE=1 SV=2**	1.000	1.339	1.170	0.797	1.308	1.310
**Proteasome subunit alpha type-7 OS=Mus musculus GN=Psma7 PE=1 SV=1**	1.140	1.174	1.157	1.621	1.759	1.674
**Proteasome subunit alpha type-5 OS=Mus musculus GN=Psma5 PE=1 SV=1**	1.027	0.977	1.002	1.301	1.575	1.317
**Aspartyl aminopeptidase OS=Mus musculus GN=Dnpep PE=1 SV=2**	26.614	39.613	33.114	52.184	50.089	41.040
**Heterogeneous nuclear ribonucleoprotein F OS=Mus musculus GN=Hnrnpf PE=1 SV=3**	1.043	1.142	1.092	1.386	1.547	1.343
**Proline synthase co-transcribed bacterial homolog protein OS=Mus musculus GN=Prosc PE=1 SV=1**	1.000	1.172	1.086	1.670	1.485	2.142
**U4/U6.U5 tri-snRNP-associated protein 1 OS=Mus musculus GN=Sart1 PE=1 SV=1**	0.918	1.139	1.028	1.373	1.203	1.306
**Suppressor of G2 allele of SKP1 homolog OS=Mus musculus GN=Sugt1 PE=2 SV=3**	1.026	0.944	0.985	1.194	1.436	1.418
